# Critically Evaluated Energy Levels and Spectral Lines of Singly Ionized Indium (In II)

**DOI:** 10.6028/jres.118.004

**Published:** 2013-01-14

**Authors:** A Kramida

**Affiliations:** National Institute of Standards and Technology, Gaithersburg, MD 20899

**Keywords:** atomic energy levels, hyperfine structure, ionization potentials, singly ionized indium, spectral lines, transition probabilities

## Abstract

A comprehensive list of the best measured wavelengths in the In II spectrum has been compiled. Uncertainties of the wavelength measurements have been analyzed, and existing inconsistencies have been resolved. An optimized set of fine-structure energy levels that fits all observed wavelengths has been derived. Uncertainties of the energy level values have been reduced by an order of magnitude. An improved value of the ionization limit of In II has been determined by fitting quantum-defect and polarization formulas for several series of levels. Intensities of lines observed by different authors have been analyzed and converted to a uniform scale. A set of recommended values of radiative transition rates has been critically compiled, and uncertainties of these rates have been estimated. The hyperfine structure interval in the 5s ^2^S ground state of In III has been determined from the measurements of the 5s*n*g and 5s*n*h series in In II.

## 1. Introduction

Singly ionized indium, isoelectronic with Cd I, has a ground state [Kr]4d^10^5s^2 1^S_0_. Its photon emission spectrum has been investigated by a number of researchers since the beginning of the 20^th^ century (see the current bibliography on this spectrum in the Atomic Energy Levels and Spectra Bibliographic Database [1] of the National Institute of Standards and Technology (NIST)). The most important of the early contributions is the work of Paschen and Campbell [2]. They presented a list of about 500 spectral lines of In II, 325 of which had partially resolved and analyzed hyperfine structure (hfs) patterns. From those observed spectral lines they derived a list of 180 fine-structure energy levels and derived the first ionization limit at 152195 cm^−1^. Paschen and Campbell observed the spectrum from 2078 Å to 9246 Å, but adopted a value of 43349 cm^−1^ for the 5s^2 1^S_0_ – 5s5p ^3^P°_1_ separation, as determined earlier by Lang and Sawyer [3] whose observations covered a wider range from 680 Å to 7277 Å. The energy levels given in the Atomic Energy Levels compilation (AEL) by Moore [4] were quoted from Paschen and Campbell [1]. The spectrum from 680 Å to 5122 Å was re-observed in 1969 by Bhatia [5] who identified many transitions previously unknown. Sansonetti and Martin [6] re-analyzed the fine-structure data of Paschen and Campbell together with Bhatia’s observations and found that the positions of all excited levels above and including the 5s6s ^3^S_1_ level, as well as the ionization limit, have to be shifted upwards by 4.37(30) cm^−1^ from the values given by Paschen and Campbell.

Interest in the In II spectrum greatly increased in the early 1980s, when it was realized that the strongly forbidden 5s^2 1^S_0_ – 5s5p ^3^P°_0_ transition can be exploited in single-ion traps to construct a high-precision atomic clock [7]. This transition, strictly forbidden in unperturbed atomic spectra, is induced by the hyperfine interaction. With the clock application in mind, Larkins and Hannaford [8] have measured the absolute frequencies of hyperfine components of several transitions in ^115^In^+^, from which they derived the corresponding energy levels and hfs interaction constants. In particular, the wave number of the above-mentioned clock transition was found to be 42275.986(7) cm^−1^. This result was confirmed two years later by Peik *et al.* [9]. In 2001, von Zanthier *et al.* [10] refined this wave number by many orders of magnitude. Their result, 42275.995245348(8) cm^−1^, agrees with both previous measurements.

In the same year 2001, Karlsson and Litzén [11] accurately measured wavelengths of 53 In II lines using a Fourier transform spectrometer. From these measurements, they derived values for 38 energy levels of In II, much improved compared to Moore’s AEL [4]. For the 5s5p ^3^P°_0_ level, Karlsson and Litzén obtained 42275.997 cm^−1^. They did not specify the uncertainty of this value, but mentioned that all their energy level values have uncertainties ranging from ±0.001 cm^−1^ to ±0.020 cm^−1^. Their result for the forbidden 5s^2 1^S_0_ – 5s5p ^3^P°_0_ transition is in good agreement with all previously mentioned data. The agreement is not so good for other transitions. In particular, for the four other transitions measured by Larkins and Hannaford [8], the wave numbers measured by Karlsson and Litzén disagree by amounts far exceeding the combined measurement uncertainties. For the energy levels above 90000 cm^−1^, Karlsson and Litzén arrived at conclusions similar to those of Sansonetti and Martin [6]. Namely, they concluded that all these levels and the ionization limit determined by Paschen and Campbell [2] should be shifted upwards by 5.16 cm^−1^. This shift value is greater than that determined by Sansonetti and Martin by 0.79 cm^−1^. Since the lines observed by Karlsson and Litzén represent a small subset of all lines observed by Paschen and Campbell and by Bhatia, this indicates that the level shifts are not the same for all levels. The individual level shifts should be determined by re-analyzing all observed data accounting for measurement uncertainties. It was noted by Smirnov [12] that, by combining the highly accurate levels values from Karlsson and Litzén [11] with wavenumbers measured by Paschen and Campbell [2], it is possible to determine many of the levels not included in [11] with uncertainties as small as ±0.01 cm^−1^.

Karlsson and Litzén [11] pointed out that the levels 5s5d ^1^D_2_ and 5p^2 1^D_2_ are strongly mixed, and their designations as given in Moore’s AEL [4] should be reversed. This revision was not reflected in Sansonetti and Martin [6].

Another problem not addressed previously is related to relative intensities of observed lines. In the selection of In II lines included in the NIST publications [13,14], the line intensities were determined by summing up the intensities of the hyperfine components, as given by Paschen and Campbell [2]. However, the latter authors used several different spectrographs, some with prisms and some with gratings, and, in the case of gratings, observed lines in different orders of diffraction. Intensities of lines registered by different methods were actually on different scales. According to my analysis, the sensitivity of different registration methods used by Paschen and Campbell varies greatly (often by orders of magnitude), and the corresponding intensity scales have different wavelength dependences. In addition to that, other observers (e.g., Lang and Sawyer [3], Bhatia [5], Karlsson and Litzén [11], and Wagatsuma [15]) report vastly different relative intensities, which are due to the different light sources and spectral registration equipment used. In order to make a comprehensive list of observed lines, the various intensity scales have to be analyzed, and the differences have to be removed to place all intensities on a uniform scale.

One problem with interpreting the In II spectrum is due to wide hfs patterns in most lines observed in the visible range. Indium has two naturally occurring isotopes, ^113^In and ^115^In, with abundances of 4.3 % and 95.7 %, respectively [6]. Both isotopes have the same nuclear spin of 9/2 and similar values of the nuclear magnetic moment, +5.5289 *μ*_N_ and +5.5408 *μ*_N_ [16]. Thus, the hyperfine structures are very similar for both isotopes. The lines from the rare isotope ^113^In are very hard to observe with samples of natural indium due to their low intensity. In this paper, most of the discussion refers to ^115^In. The hyperfine structure and isotope shifts will be addressed only insofar as they concern the accuracy of the derived center-of-gravity values of energy levels and observed wave numbers of fine-structure transitions.

The aims of the present study are: 1 analyze the measurement uncertainties of different studies, explain and remove inconsistencies, and build a comprehensive list of the best measured wavelengths; 2) from this line list, determine the optimized set of energy levels that fit all observed wavelengths; 3) determine an improved value of the ionization limit; 4) analyze line intensities observed by different authors and convert them to a uniform scale; 5) compile a list of recommended values of radiative transition rates. These objectives are addressed in the following sections.

## 2. Evaluation of Measured Wavelengths

The total list of observed lines compiled in Table 1 includes 680 lines selected from Karlsson and Litzén [11], Larkins and Hannaford [8], von Zanthier *et al.* [10], Paschen and Campbell [2], Bhatia [5], Lang and Sawyer [3], and Wagatsuma [15]. The first four of these sources provide data that are sufficiently accurate to determine all energy levels involved in other observations. Measurements of Bhatia [5], Lang and Sawyer [3], and Wagatsuma [15] were not included in the level optimization procedure because they were found to possess significant systematic and statistical errors and do not improve the level values.

In Table 1, the wavelengths below 2000 Å are given in vacuum, and above that in standard air. Conversion from vacuum to air and vice versa was made using the five-parameter formula from Peck and Reeder [28]. The uncertainties in the units of the least significant digit of the value are given in parentheses after the value. These uncertainties do not account for the uncertainties of the vacuum-air conversion formula. All uncertainties are meant to be on the level of one standard deviation. Where the systematic uncertainties are significant, they are given in addition to the statistical ones.

A detailed analysis of all observations follows.

### 2.1 Measurements of Karlsson and Litzén [11]

Karlsson and Litzén [11] recorded spectra of indium in the region (12500–55500) cm^−1^ (i.e., from 8000 Å to 1800 Å) using a vacuum ultraviolet (VUV) Fourier transform spectrometer. The light source was a hollow cathode discharge with the carrier gas consisting of neon at 170 Pa (1.3 Torr) or an argon–neon 1:1 mixture at 160 Pa (1.2 Torr). Small pieces of metallic indium were placed inside the water-cooled iron cathode.

The wavenumber scale was calibrated by means of Ar II lines [17] in the ultraviolet (UV) region, whereas Ne I and Ne II lines were used at longer wavelengths. The neon lines had been measured with high accuracy relative to Fe I and Fe II lines [18,19] during previous experiments using an iron cathode and neon as the carrier gas. Since the above-mentioned Fe I–II measurements were calibrated against Ar II wave numbers of Norlén [17], all In II wave numbers measured by Karlsson and Litzén [11] can be ultimately traced to the Ar II reference data of Norlén. It was recently found by Nave and Sansonetti [20] that the wavenumber scale used by Norlén has a calibration error, and all wave numbers from his paper have to be increased by 6.7 parts in 10^8^. I applied this correction factor to all In II wave numbers reported in Ref. [11].

To analyze the measurement uncertainties, one has to take into account the hfs of the observed lines, as well as statistical and systematic uncertainties. Karlsson and Litzén noted that most In II lines exhibit wide hfs extending over several reciprocal centimeters. One line at 2941.0375 Å corresponding to the 5s5p ^1^P°_1_ – 5s6s ^1^S_0_ transition was observed as a symmetric feature with no discernible hfs. This line was denoted as a category ‘a’ in Table 1 of Ref. [11] and was fitted with a Voigt profile. Some of the lines have well-resolved hfs with up to 16 distinct components. For these lines, denoted as a category ‘c’ in [11], Karlsson and Litzén modeled the hfs with an account for the linear and quadratic magnetic hfs and determined the center-of-gravity wave number from this fitting procedure. For lines where the hfs was noticeable but the fitting procedure did not give an unambiguous result due to a lack of resolution or a too low signal-to-noise ratio (*S/N*), the center of gravity of the observed hfs pattern was derived by fitting the observed feature using an empirical procedure. These lines were denoted as a category ‘b’ in Ref. [11].

In the presence of such a large hfs splitting, the positions of centers of gravity of observed features do not necessarily coincide with the differences between centers of gravity of the hfs components of the upper and lower levels, because they depend on the distribution of intensities within the hfs multiplets. This is true even if the population of the hfs sublevels follows the Boltzmann distribution (see the discussion of this effect in relation to the fine structure in the hydrogen spectrum [21]). In such a case, the energy levels derived from the centers of gravity of observed features are only approximate and depend on the observational conditions, which may distort the distribution of intensities and thus shift the centers of gravities of the features. Such approximate energy levels were called *distinctive energy levels* in [21]. In the case of the In II spectrum, where the hfs is not completely resolved in most lines, only such distinctive energy levels can be determined. They have an intrinsic uncertainty, which is a sizable fraction of the total breadth of the hfs. Thus, even if the centers of gravity of the observed hfs are determined precisely, we can expect them to deviate from the Ritz values due to the intrinsic inaccuracy of the distinctive energy levels.

We can also expect the lines from the category b of Karlsson and Litzén to have the largest uncertainties due to inaccuracies of the measurement of centers of gravity of the hfs.

To estimate the wave number uncertainties, as a zero approximation, I neglected the hfs considerations and assumed that the measurement uncertainties *δσ* are entirely due to statistical and systematic uncertainties (*δσ*_stat_ and *δσ*_syst_, respectively) in the wave number measurements of symmetric well-resolved features (see, for example, Kramida and Nave [22]:

(1) $$\delta \sigma \approx {\left(\delta \sigma_{\text{stat}}{}^{2}+\delta \sigma_{\text{syst}}{}^{2}\right)}^{1/2},$$

(2) $$\delta \sigma_{\text{stat}}\approx W/(2S/N),$$

where *W* is the full width at half-maximum. The *S/N* values are given in Table 1 of Karlsson and Litzén [11] in the column of line intensities. The values of *W* and *δσ*_syst_ can be estimated as 0.02 cm^−1^ and 0.003 cm^−1^, respectively, from the statement that the uncertainty of the wave numbers varies from ± 0.003 cm^−1^ for strong unblended lines to ± 0.02 cm^−1^ for weak, unresolved, or blended lines [11]. However, I found that setting

(3) $$W=0.03\;{\text{cm}}^{-1}$$

results in better statistical consistency of the energy levels with the measured wave numbers. The value of 0.003 cm^−1^ for the systematic uncertainty corresponds to the maximum value of the wavenumber scale correction described above. With these assumed wavenumber uncertainty values, using the level-optimization code LOPT [23], I have derived a set of optimized energy levels from the observed wave numbers given in Table 1 of Karlsson and Litzén [11]. The resulting values agreed with those given in Ref. [11] with an average deviation of 0.003 cm^−1^, the maximum deviation being 0.006 cm^−1^. This close agreement indicates that the assumed wavenumber uncertainties are close to those used by Karlsson and Litzén in their level-optimization procedure.

The final assignment of wavenumber uncertainties was made when all measured lines from different sets of measurements were brought together in a comprehensive line list and checked for internal consistency (see Sec. 3). At that stage, it was found that the lines from the categories a and b of Karlsson and Litzén [11] deviate from the Ritz values by much greater amounts than those of the category c, which is consistent with the expectations noted above. Therefore, the uncertainties of these categories of lines have been increased by a factor of 3.7 as compared to Eq. (3). Only then can the measurements of Karlsson and Litzén [11] be considered consistent with those of Paschen and Campbell [2] and with other observations.

### 2.2 Measurements of Larkins and Hannaford [8]

Larkins and Hannaford [8] measured the absolute frequencies of hyperfine components of the four lines at 1936 Å (5s5p ^3^P°_0_ – 5s6s ^3^S_1_), 1977 Å (5s5p ^3^P°_1_ – 5s6s ^3^S_1_), 2079 Å (5s5p ^3^P°_2_ – 5s6s ^3^S_1_), and 2306 Å (5s^2 1^S_0_ – 5s5p ^3^P°_1_) emitted by a hollow-cathode lamp by using a 2.5 m scanning monochromator. The monochromator was fitted with a 316 lines/mm échelle grating having a blaze angle of 65° and mounted in a Czerny-Turner configuration. The four In II lines listed above were measured in different orders of diffraction (29^th^ for the first two lines, 27^th^ for the third one, and 24^th^ for the fourth). The absolute frequency scale was calibrated separately for each of the four lines using different sets of Fe I and Fe II reference lines, for which the Ritz wavelengths from Nave *et al.* [19] and O’Brian *et al.* [24] were used. The orders of diffraction for the reference lines were different for different Fe lines and, most importantly, for different In II lines. Larkins and Hannaford assumed the calibration uncertainty to be equal to the quadrature sum of the quoted uncertainties in the energies of the upper and lower Fe levels of the reference lines [19,24]. However, as noted by Reader [25], this method of calibration that uses reference lines in an order of diffraction different from that of the measured line can cause a significant systematic error in a Czerny-Turner spectrometer. These systematic errors are the most probable cause of the discrepancies between the wave numbers measured by Larkins and Hannaford [8] and by Karlsson and Litzén [11] mentioned in the previous section. The suspected culprit of these systematic errors is the variation of the refraction index of air with pressure. Larkins and Hannaford made all measurements in air and used a formula for conversion from air wavelengths to vacuum wave numbers that does not account for the air pressure, temperature, and composition. They did not mention anything about the air conditions in their setting. If the grating is assumed to be ideal, the observed wavelength of any line in any order of diffraction is equal to the wavelength in the first order multiplied by the order number. However, the refraction index of air is different at different first-order wavelengths. Thus, if the reference and investigated lines are observed in different orders of diffraction, the variation of the refraction index of air has to be taken into account in the derivation of the measured wavelength. My numerical experiments showed that possible variations of the atmospheric air pressure between 101.2 kPa and 104.8 kPa in the experiment of Larkins and Hannaford [8] can explain all discrepancies between their measurements and those of Karlsson and Litzén [11] (the average air pressure at the site of measurements in Clayton, Australia, is 101.11 kPa).

It should be noted that the Fe I and Fe II wave numbers given in Refs. [19,24] were calibrated against the Ar II reference lines from Norlén [17] and thus have to be increased by 6.7 parts in 10^8^ (see the discussion in the previous section). The difference between the reference Fe wavenumbers as given by Larkins and Hannaford from the corrected ones given in the current version of the NIST Atomic Spectra Database [26] amounts to 0.004 cm^−1^, which is much smaller than the discrepancies in the measured In II wave numbers.

In the absence of reliable means to correct the calibration errors of Larkins and Hannaford [8], the measurements of Karlsson and Litzén [11] should be preferred, despite the fact that they [11] possess significantly greater statistical uncertainties. However, for one transition, 5s^2 1^S_0_ – 5s5p ^3^P_1_, the measurement of Larkins and Hannaford [8] can be re-calibrated using a recent independent high-precision measurement of an hfs component of this line. Namely, Wang *et al.* [27] measured the absolute frequency of the 5s^2 1^S_0_ (F=9/2) – 5s5p ^3^P_1_ (F=11/2) transition in a single ^115^In^+^ ion cooled in a radio-frequency trap by using a frequency comb referenced to a Cs atomic clock. Converted to wave number units, their result is 43351.622760(3) cm^−1^. The wave number of this transition, as measured by Larkins and Hannaford [8], is 43351.614(2) cm^−1^. The difference is 0.009 cm^−1^, which is almost twice as large as the systematic uncertainty assumed by Larkins and Hannaford. By utilizing the air wavelengths of the three hfs components of the 5s^2 1^S_0_ – 5s5p ^3^P_1_ transition, which are given by Larkins and Hannaford with relatively greater precision, I obtained 43350.5817(15) cm^−1^ for the corrected wave number of the centroid of this transition.

### 2.3 Measurements of Paschen and Campbell [2]

Paschen and Campbell [2] observed the In II spectrum excited in the negative glow of a hollow carbon cathode discharge filled with helium. The spectrum was dispersed in the region 2350 Å through 9217 Å using a 4 m concave grating spectrograph in various orders of diffraction (first through eighth) and recorded on photographic plates. Additional measurements of weaker lines were made with a higher-throughput single-prism spectrograph in the region 2078 Å through 6627 Å and a three-prism spectrograph in the region 3802 Å through 4229 Å. The wavelength scale was calibrated against known lines of helium, neon, and iron. Paschen and Campbell listed about 1400 measured wave numbers of lines belonging to about 500 transitions of In II. Many of the lines had completely resolved hfs patterns, but most of them were resolved only partially. For all lines, Paschen and Campbell gave an estimated center-of-gravity wavenumber (averaged over the hyperfine structure) without an uncertainty estimate. Thus, in order to obtain an optimized set of energy levels, it is necessary to estimate the uncertainty for each line.

The number of decimal places in the wave numbers listed by Paschen and Campbell can serve as a guide to their uncertainties. Most of the wave numbers of hfs components in the red region of the spectrum measured with the grating spectrograph were given with three digits after the decimal point. However, the center-of-gravity wave numbers were given with only two digits after the point, which indicates a reasonable loss of accuracy in the averaging. The nominal resolution of the grating spectrograph was *R* ≡ *λ*/Δ*λ* ≈ 82500 in the first order of diffraction [2], so one can expect the uncertainty of the wave numbers *σ* of individual hfs components to be greater than approximately 0.1 *σ/R* ≈ 0.013 cm^−1^ at the longest wavelength 9217 Å. The finite entrance slit width and uncertainties of reference lines should further increase the uncertainty. For far-ultraviolet lines measured with the prism spectrograph, the wave numbers are given with only one digit after the decimal point, indicating a much greater uncertainty.

As a zero approximation, I assumed that the measurement uncertainties of unblended lines are equal to 20 units of the last given decimal place and twice that for blended or perturbed lines. With these uncertainties, a set of optimized energy levels was obtained with the LOPT code [23]. The resulting level values agreed with those given by Paschen and Campbell [2] with a standard deviation of 0.04 cm^−1^. However, the too small residuals (i.e., deviations of observed wave numbers from the Ritz values) indicated that the wavenumber uncertainties should be decreased in order to achieve statistical consistency. The latter requirement means that the root of sum of squares (RSS) of the residuals should be approximately equal to degrees of freedom (DF) of the problem (in this case, DF = 320). With the initial estimate of uncertainties described above, RSS/DF = 0.12, which means that the assumed uncertainties were too large on average. Better estimates can be obtained by analyzing mean values of residuals (observed minus Ritz wave number) separately for each category of lines. Division of the lines into categories is naturally defined by the different observation methods (with the single-prism, three-prism, or grating spectrograph in different orders of diffraction) and by the character of the lines (well-resolved or blended). The finally adopted uncertainty values are listed in Table 1. They vary from 0.02 cm^−1^ for a few red lines to 1.0 cm^−1^ for the blended line at 2249.6 Å. With these final uncertainty values, the level optimization procedure yields RSS/DF = 1.15, which indicates a good statistical consistency. Agreement of the optimized levels with the original values from Paschen and Campbell remained the same (on average) after the uncertainty adjustments.

The line list of Paschen and Campbell [2] includes 28 forbidden transitions. One of them is the hyperfine-induced clock transition 5s^2 1^S_0_ – 5s5p ^3^P°_0_ at 2364.686 Å, and all the others are electric-quadrupole (E2) transitions between opposite-parity levels with the change in the angular momentum Δ*J* = 2. In the absence of external fields, these transitions are highly forbidden. However, an electric field present in the discharge mixes the hyperfine components of the levels having the same total angular momentum *F* but *J* values differing by one. For example, an external electric field mixes the *F* = 15/2 component of the 5s7f ^3^F°_4_ level with the *F* = 15/2 component of the 5s7f ^3^F°_3_ level, thus enabling the normally forbidden 5s6d ^3^D_2_ – 5s7f ^3^F°_4_ transition. For such an electric-field induced transition, only a certain part of the hfs components of both levels become allowed. Thus, its observed center of gravity will be shifted from the difference between the centers of gravity of the two levels. This may cause systematic shifts in the level values derived with the level optimization procedure. To avoid such shifts, for the purpose of level optimization I have decreased the weight of these forbidden transitions by a factor of four by increasing their uncertainties with a factor of two. However, the uncertainties given in Table 1 are given without these adjustment factors.

A small inconsistency was found in the tables of Paschen and Campbell [2]. Namely, the 5s17s ^3^S_1_ level was given in [2] as 149892.67 cm^−1^. This level takes part in three observed transitions, 5s6p ^3^P0,1,2 – 5s17s ^3^S_1_ at 2367.0 Å, 2377.1 Å, and 2410.8 Å, all measured with the single-prism spectrograph. The Ritz wave number given in the line list of [2] for the ^3^P_1_–^3^S_1_ transition agrees with the listed energy levels. However, the Ritz values given for the other two transitions imply a different value of the 5s17s ^3^S_1_ level, 149891.99 cm^−1^.

### 2.4 Measurements of Bhatia [5]

The thesis of Bhatia [5] was mainly focused on the spectra of doubly and triply ionized indium. However, it also includes new energy-level classifications for about a hundred of In II lines. For another several tens of lines, Bhatia’s measurements are the most accurate available. Emission spectra of indium were photographed over the spectral range from 340 Å to 9500 Å using a disruptive electrodeless discharge and a spark in helium. About 4000 lines of indium were measured, and 36 % of these lines were classified as belonging to the In I–V spectra. The spectrum from 340 Å to 2440 Å was obtained using a 3 m grazing incidence vacuum spectrograph with a 1200 lines/mm grating giving a reciprocal dispersion of 2.775 Å/mm. The grating was blazed for 1300 Å. In the region 2300 Å to 9500 Å, a prism spectrograph was used. Different types of photographic plates were used in three spectral ranges: 340 Å to 3000 Å, 3000 Å to 6500 Å, and 4500 Å to 9500 Å. For the wavelength scale calibration in the vacuum range, C, N, and O standards were used below 1760 Å and Si standards between 1930 Å and 2297 Å. For the prism spectra, iron and neon standards were used.

Bhatia noted that the prism measurements have uncertainties greater than ±0.05 Å. This corresponds to a wavenumber uncertainty of ±0.8 cm^−1^ at 2440 Å and ±0.05 cm^−1^ at 9600 Å. For the grating measurements, I estimated uncertainties by comparing the measured wavelengths with much more accurate Ritz values derived from the measurements described in the previous sections. In the region below 1900 Å, the statistical uncertainty of well-resolved lines is ±0.03 Å; between 1900 Å and 2440 Å, it is ±0.09 Å; and above 2440 Å, it is ±0.13 Å.

All newly classified or re-measured In II lines in Bhatia’s thesis correspond to transitions between known levels determined from other, more accurate measurements. Therefore, these lines were not included in the level optimization procedure. However, for the sake of completeness, I include them in the line list and give their original observed wavelengths. They were found to contain noticeable systematic shifts, which are responsible for the difference between the values of the correction to the energy levels of Paschen and Campbell derived by different authors [6,11]. These systematic shifts were estimated by fitting deviations of Bhatia’s original wavelengths from the Ritz values with cubic polynomials. This was done separately for the prism and grating measurements. For the grating measurements, systematic shifts vary from –0.02 Å at 2430 Å to +0.01 Å at 1900 Å, –0.01 Å at 1000 Å, and +0.005 Å at 680 Å. For the prism measurements, they vary from –0.44 Å at 5123 Å to +0.10 Å at 2460 Å.

In Table 1, the observed wavelengths selected from Bhatia [5] are given with two uncertainty values in parentheses after the value. The first uncertainty is a statistical one, and the second is a systematic one.

### 2.5 Measurements of Lang and Sawyer [3]

Lang and Sawyer [3] observed the In II spectrum emitted by a hollow cathode discharge in helium or in neon. As cathodes, they used a carbon tube with indium placed inside or a tungsten tube coated with indium. The spectrum between 500 Å and 8000 Å was photographed with a 1 m vacuum grating spectrograph and with two prism spectrographs. Lang and Sawyer did not specify their measurement uncertainties, but indicated that the higher terms deduced from the vacuum ultraviolet measurements should be accurate to less than 5 cm^−1^, which corresponds to ±0.05 Å at 1000 Å.

Lang and Sawyer listed 144 observed and classified lines of In II between 680 Å and 7183 Å. All their measurements above 3273 Å have been superseded by other observers. However, in the shorter wavelength region there remain 18 lines for which their observations are the only available ones. All of the levels associated with these lines are accurately established by other observations, so I did not use the lines from [3] in the level optimization procedure. Nevertheless, I include them in Table 1 with their original wavelengths. They were found to contain significant systematic shifts. These shifts were estimated by fitting deviations of the original wavelengths from the Ritz values with a cubic polynomial. The systematic shifts vary from –0.25 Å at 680 Å to –0.07 Å at 1586 Å and –0.14 Å at 3273 Å, while the average statistical uncertainty is ±0.11 Å below 2300 Å and ±0.09 Å above that.

Similar to Bhatia’s measurements, the observed wavelengths selected from Lang and Sawyer [3] are given in Table 1 with two uncertainties, statistical and systematic, given in parentheses after the value.

Five lines listed by Lang and Sawyer [3] at 1133.15 Å, 1625.36 Å, 1681.72 Å, 2446.02 Å, and 3274.11 Å do not match any combination between the known energy levels. For the line at 1133.15 Å, I found that Lang and Sawyer made an error in conversion from the correctly given wavenumber 87249 cm^−1^. The correct wavelength is 1146.14 Å. The line at 1625.36 was classified by Bhatia [5] as belonging to In III. For the line at 3274.11 Å, the wave number was misprinted in Lang and Sawyer as 30534, while the correct value is 30543 cm^−1^. Thus, the correct wavelength is 3273.13 Å. Paschen and Campbell [2] noted that this line was expected to occur in their spectra, but they could not detect it. The origins of the other two lines are unknown. Thus, they are omitted from Table 1.

### 2.6 Measurements of Wagatsuma [15]

Wagatsuma [15] observed emission spectra of several elements, including indium, from Grimm glow discharge plasmas with argon, neon, and mixtures of argon with helium and neon with helium as buffer gases. The main purpose was to investigate relative intensities of spectral lines in different buffer gases. The Grimm-type discharge features a hollow anode, and a plain sample is placed close to the anode opening. A Fastie-Ebert mounting spectrograph with a focal length of 3.4 m equipped with a photomultiplier detector was employed to measure the spectra in the wavelength region from 2300 Å to 8000 Å. The grating had 1200 lines/mm and was blazed for 3000 Å. Emission spectra in the shorter wavelength region, 1600 Å to 2450 Å, were recorded on a 2.0 m Eagle-mounting vacuum spectrometer with a 1200 lines/mm grating blazed at 1700 Å, equipped with a CaF_2_ window and a photomultiplier tube.

Wagatsuma reported intensity measurements for 43 lines of In II between 1571 Å and 6116 Å. He did not give his measured wavelengths; instead, he listed the Ritz wavelengths calculated from the energy levels given by Moore [4]. Most of the lines were observed and classified elsewhere. However, two lines were newly classified. These are the lines at 1657.43 Å (5s5p ^1^P°_1_ – 5s7s ^1^S_0_ and 3842.92 Å (5p^2 1^D_2_ – 5s4f ^3^F°_2_). Since no wavelength measurements are available, I give the Ritz wavelengths for these two lines in Table 1.

## 3. Optimized Energy Levels and the Ionization Limit

After the wavelength measurement uncertainties have been assessed, the level optimization is a straightforward procedure. For that I used the least-squares level optimization code LOPT [23]. As noted above, only the measurements of Karlsson and Litzén [11], Larkins and Hannaford [8], von Zanthier *et al.* [10], and Paschen and Campbell [2] were included in the level optimization procedure, which makes a total of 495 lines. Although high-precision measurements constitute only about 10 % of all included lines, they have a dramatic effect on the accuracy of the derived excitation energies, decreasing the uncertainties from a few reciprocal centimeters for the level list of Paschen and Campbell [2] to 0.2 cm^−1^ on average. More than half of the levels have uncertainties below 0.1 cm^−1^, and 75 of them are accurate to better than ±0.05 cm^−1^.

The average shift of the newly optimized levels above 63000 cm^−1^ from those given by Paschen and Campbell [2] is 4.88 cm^−1^ with a standard deviation of 0.18 cm^−1^. For two levels from the list of Paschen and Campbell, 5s9p ^3^P°_2_ and 5s11p ^3^P°_1_, there are no observed lines in their line list. Transitions from these levels were observed by Bhatia [5] (one from 5s9p ^3^P°_2_ and four from 5s11p ^3^P°_1_). Their measurement uncertainties are between 1.6 cm^−1^ and 1.9 cm^−1^, which is much greater than uncertainties in the measurements of Paschen and Campbell. I assumed that those lines were actually observed by Paschen and Campbell but omitted from their line list. Therefore, the values given for these levels in Table 2 are derived from those of Paschen and Campbell by adding the average shift mentioned above.

As can be seen in Table 2, there are several Rydberg series accurately measured up to high values of principal quantum number *n*, such as 5s*n*d ^3^D_3_ (*n* = 5–19), 5s*n*d ^3^D_2_ and ^3^D_1_ (*n* = 5–18), 5s*n*s ^3^S_1_ (*n* = 5–17), 5s*n*g (*n* = 5–14), and 5s*n*h (*n* = 8–14). These series are unperturbed and thus can be used to determine the ionization limit. I used computer codes written by Sansonetti [29] to fit Ritz-type quantum-defect formulas for the 5s*n*s and 5s*n*d series and the polarization formula for the 5s*n*g and 5s*n*h series. Thus, I have obtained five values for the ionization limit derived with different methods, one with the polarization formula and four with quantum-defect formulas. The weighted average of these five values is 152200.10 cm^−1^ with ±0.22 cm^−1^ adopted as a conservative estimate of uncertainty.

From the same or similar series formulas, several unobserved levels, such as 5s6h, 5s7h, 5s15d ^3^D_1_, 5s19d ^3^D_1_, 5s19d ^3^D_2_, 5s18s ^3^S_1_, 5s19s ^3^S_1_, and 5s*n*s ^1^S_0_ (*n* = 17–19), could be accurately determined. For two levels, 5s12p ^1^P°_1_ and 5s17d ^3^D_3_, the values derived from the series formulas are significantly more accurate than the measured ones. These interpolated or extrapolated values are included in Table 2.

The only two high-precision energy values in Table 2, i.e., those for the 5s5p ^3^P°_0_ and 5s5p ^3^P°_1_ levels, are pertinent to the isotope ^115^In, while the rest of the level values were determined from wavelengths of lines observed in a natural mixture of isotopes. The only available experimental measurement of the isotope shift (IS) for In^+^ was made by Wang *et al.* [27]. These authors have measured the IS between the energies of the 5s5p ^3^P°_1_ level in ^113^In^+^ and ^115^In^+^ to be 0.02321(6) cm^−1^. Since the abundance of ^113^In in natural samples is only 4.3 % [6], the value of the 5s5p ^3^P°_1_ level for the natural mixture of indium isotopes is lower than that of ^115^In^+^ by 0.0010 cm^−1^. Most of the levels given in Table 2 are ultimately based on 5s5p ^3^P°_1_. Therefore, they are systematically too high by this amount. Since the uncertainties of the level values are much greater, this shift has been neglected.

## 4. Theoretical Interpretation of the Energy Levels

To find eigenvector compositions of the levels and calculate transition probabilities, I used a parametric fitting with Cowan’s computer codes [30]. The following configurations were included in the calculations: 5s^2^, 5s*n*s (*n* = 6–19), 5s*n*d (*n* = 5–10), 5s*n*g (*n* = 5–10), and 5p^2^ for even parity, and 5s*n*p (*n* = 5–10), 5s*n*f (*n* = 4–10), and 5s*n*h (*n* = 6–10) for odd parity. In the even parity set, 79 known levels were fitted with 35 free parameters with an average deviation of 16 cm^−1^. In the odd parity set, 60 known levels were fitted with 25 free parameters with an average deviation of 13 cm^−1^. Percentage compositions of the levels included in Table 2 are from these calculations. The fitted parameters were used to calculate the transition probabilities.

The percentage compositions of the levels given in Table 2 result from the parametric fitting described above. In this table, the energies of the 5s*n*p ^3^P°_0_ levels with *n* = 8–10, which were not observed experimentally, are from the same parametric fitting. The value for 5s8p ^3^P°_0_, 135823(10) cm^−1^, is in fair agreement with the result of the parametric fitting by Biémont and Zeippen [35], 135833 cm^−1^.

## 5. Interpretation of the 5s*n*g and 5s*n*h Series

Paschen and Campbell [2] found that the 5s*n*g and 5s*n*h configurations are split into pairs of closely located level groups, which they designated as *n*–G, *n*+G, *n*–H, and *n*+H. The 5s*n*g series was observed for *n* = 5–14, and the 5s*n*h series for *n* = 8–14. The separations within the pairs (e.g., between *n*–G and *n*+G) are almost constant along the series and are equal to 3.56(3) cm^−1^ for the 5s*n*g (*n* = 7–14) series and 3.58(8) cm^−1^ for the 5s*n*h (*n* = 8–14). The separations within the 5s5g and 5s6g level pairs are slightly greater, 3.91(8) cm^−1^ and 3.71(10) cm^−1^, respectively. The almost constant separations for *n* ≥ 7 imply that their probable cause is the hyperfine splitting of the In III 5s core. Indeed, the predicted hfs interval in the ^115^In III 5s ground state is 3.56 cm^−1^ [31]. There are no measurements of this interval in In III. However, it was measured in the isoelectronic spectra of ^107^Ag I [32] and ^111^Cd II [33]. These measurements agree with the calculations of Beck and Datta [31] within 3 %, which suggests that their result for ^115^In III quoted above is similarly accurate. The close agreement between the theoretical hfs interval of ^115^In III 5s and the observed separations in the In II 5s*n*g and 5s*n*h series suggests that these observed separations represent the first measurement of the hfs interval in ^115^In III 5s. The weighted mean of this interval, averaged over the high members of both the 5s*n*g and 5s*n*h series (*n* ≥ 7) is 3.56(3) cm^−1^.

Thus, these two series represent the first observation of an unusual coupling between the fine-structure and hfs interactions in an atomic system where the hfs interaction dominates over the fine-structure effects such as the spin-orbit splitting and electrostatic exchange. According to my Cowan code calculations (see the previous section), the average fine-structure splittings caused by the spin-orbit splitting and electrostatic exchange are the largest for the 5s5g configuration and amount to about 2 cm^−1^. They rapidly decrease with increasing *n* along the 5s*n*g series and are smaller than 1 cm^−1^ for *n* ≥ 9. For all members of the 5s*n*h series, the fine-structure splittings are smaller than 0.0002 cm^−1^. These splittings are indeed much smaller than the hfs interval in ^115^In III 5s, which may explain the observed constant intervals within the pairs along the series, as well as the increased intervals in 5s5g and 5s6g. A similar behavior of the level intervals along the 1s*n*f, 1s*n*g, 1s*n*h, 1s*n*i, and 1s*n*k series was predicted by Morton *et al.* [34] for ^3^He I, where the hfs interval in the ^3^He II 1s core, –8666 MHz, is much greater (in absolute value) than the fine-structure splittings. The latter are smaller than 400 Mhz for all members of these series.

Because of this unusual coupling, no *LS* term designations can be assigned to the 5s*n*g and 5s*n*h series. Instead, I designate Paschen and Campbell’s *n*–G, *n*+G, *n*–H, and *n*+H levels as 5s_1/2,_*_F_*_=4_*n*g, 5s_1/2,_*_F_*_=5_*n*g, 5s_1/2,_*_F_*_=4_*n*h, and 5s_1/2,_*_F_*_=5_*n*h, respectively. Each of these levels is comprised of multiple sublevels arising from coupling of the angular momentum *F* of the core with the angular momentum *J*_2_ of the outer electron. For the lower members of the 5s*n*g series, the intervals between the sublevels are comparable to the interval between the *F* = 4 and *F* = 5 hyperfine components of the core, while for the higher members of this series, as well as for the entire 5s*n*h series, they are much smaller.

## 6. Relative Intensities of Observed Lines

As noted in the Introduction, relative intensities of lines observed with different light sources and with different registration equipment are vastly different. In order to give a consistent set of relative intensities, they must be converted to the same scale. To account for the different excitation conditions in various light sources, the observed line intensities can be approximated by local thermodynamic equilibrium (LTE) with certain excitation temperature pertinent to each light source, and then scaled to the same excitation temperature. In reality, the LTE approximation describes the observed intensities only qualitatively, with deviations in both directions up to an order of magnitude. However, this method results in much better qualitative agreement between relative intensities observed by different authors. Besides the different effective temperatures in the light sources, the observed intensities are strongly affected by different behaviors of spectral response functions of the registration equipment at different wavelengths. These variations can also be accounted for and removed. These procedures are described below. The general method relies on radiative transition rates *A_ki_* calculated with Cowan’s codes (see Sec. 4), and on the LTE relation between these transition rates and the observed intensities *I*_obs_:

(4) $$I_{\text{obs}}\propto (g_{k}A_{\mathrm{ki}}/\lambda )\exp(-E_{k}/kT_{\text{eff}}),$$

where *g*_k_ and *E_k_* are the statistical weight and energy of the upper level, *λ* is the central wavelength of the line, *k* is the Boltzmann constant, and *T*_eff_ is the effective temperature.

### 6.1 Line Intensities in the Spectra Observed by Karlsson and Litzén [11]

As noted in Sec. 2.1, the intensities given by Karlsson and Litzén [11] for their FTS measurements are roughly the signal-to-noise ratios. The effective temperature can be easily found from the Boltzmann plot, i.e., from the linear slope of *I*_obs_ *λ /* (*g_k_A_ki_*) versus the energy of the upper level *E_k_*. Intensities reported by Karlsson and Litzén [11] are well described by effective temperature of *T*_eff_ ≈ 1.1 eV. No wavelength dependence of the registration sensitivity was found for their observations.

### 6.2 Line Intensities in the Spectra Observed by Paschen and Campbell [2]

**Fig. 1:**
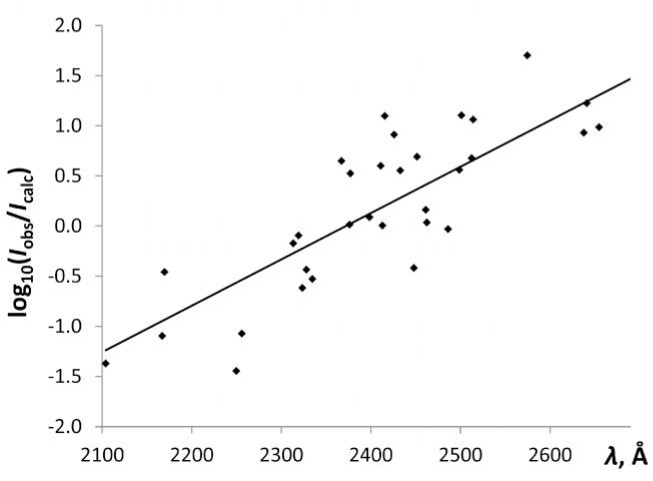
A logarithmic plot of the spectral sensitivity of single-prism observations of Paschen and Campbell [2] against wavelength. The solid line is a linear least-squares fit to the data points.

The analysis of line intensities reported by Paschen and Campbell [2] is complicated due to the large number of different registration methods used. The analysis is simplest for the observations made with the single-prism spectrograph. These observations span the wavelength region between 2100 Å and 6630 Å, but most of them are below 2700 Å. The intensities reported by Paschen and Campbell are visual estimates of blackening of photographic plates; hence, they are non-linear in response to exposure. This non-linearity should be removed together with the wavelength dependence of the sensitivity. This was done in an iterative procedure, which resulted in an effective temperature *T*_eff_ ≈ 3.4 eV. The spectral variation of the sensitivity can be visualized by plotting the logarithm of the ratio of calculated and observed intensities *I*_obs_/*I*_calc_, where *I*_calc_ is set to equal the right side of Eq. (4), against wavelength. This plot for the single-prism observations is presented in Fig. 1.

As seen from this figure, the sensitivity of the single-prism observations exponentially drops by three orders of magnitude when the wavelength decreases from 2700 Å to 2100 Å.

**Fig. 2:**
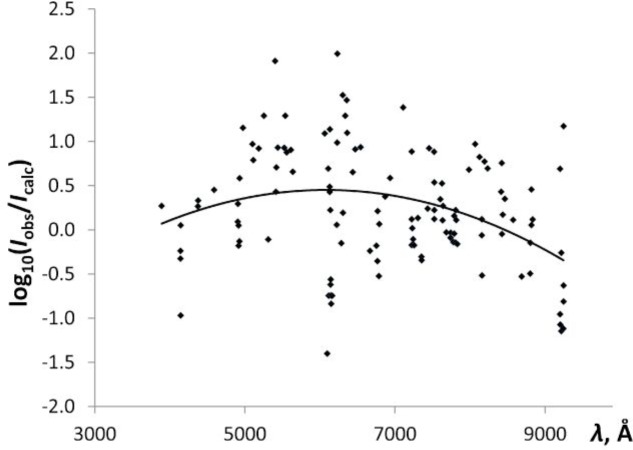
A logarithmic plot of the spectral sensitivity of grating observations of Paschen and Campbell [2] in the first order of diffraction against wavelength. The solid line is a quadratic least-squares fit to the data points.

Similar plots were built for observations made with the three-prism spectrograph and with the grating spectra in various orders of diffraction. As a sample, the plot of the spectral sensitivity of the grating observations in the first order of diffraction is given in Fig. 2.

**Fig. 3:**
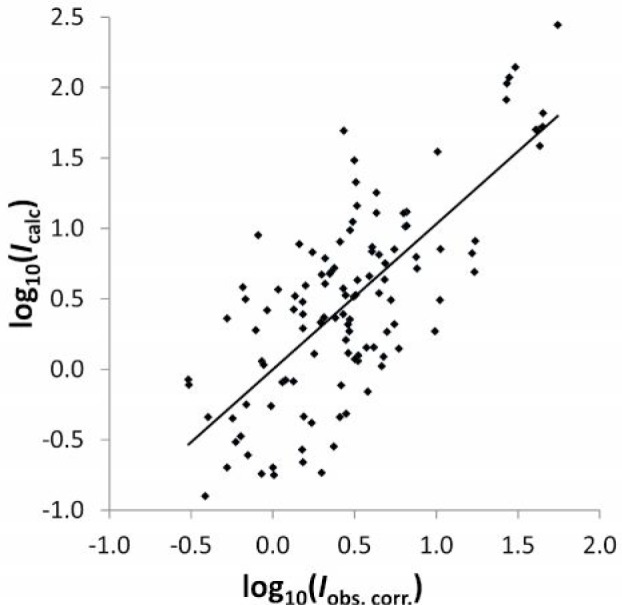
Verification of linearity of the corrected intensity scale for the observations of Paschen and Campbell [2]. The solid curve is a linear fit to the data points. Its slope is close to one, and it nearly crosses the (0, 0) point.

To verify that the non-linearity was correctly removed from the intensity values, the logarithm of the calculated intensities is plotted against the corrected observed intensities in Fig. 3. In this plot, the wavelength dependence was removed from all observations.

Removal of the wavelength dependence of sensitivity and non-linearity of the intensity scale has a dramatic effect on the values of relative intensities. For example, the ratio of relative intensities of the allowed 5s^2 1^S_0_ – 5s5p ^3^P°_1_ transition at 2306 Å and the highly forbidden hyperfine-induced 5s^2 1^S_0_ – 5s5p ^3^P°_0_ transition at 2365 Å, as given by Paschen and Campbell [2], is 3.3, while the ratio of the corrected intensities is 75.

### 6.3 Line Intensities in the Spectra Observed by Bhatia [5]

Analysis of intensities observed by Bhatia [5] presented the greatest difficulties for several reasons. First, as noted in Sec. 2.4, the great majority of the lines observed in this work belong to higher ionization stages. Out of the total 4000 lines, only about 300 could be ascribed to In II. Thus, blending with lines of other ionization stages can be expected to distort intensities of many In II lines. Second, a significant number of the lines were overexposed. Due to a strong non-linearity of the response of photographic plates at high exposures, intensities of strong lines are greatly underestimated. Therefore, when analyzing the intensities from Bhatia [5], overexposed lines and lines suspected to be perturbed by other ionization stages were excluded from consideration.

The analysis was made using an approach similar to the one described above. Namely, the intensities of the lines in different spectral regions were treated separately, assuming that the response of the registration equipment has a different dependence on wavelength in those regions.

The effective excitation temperature describing the line intensities observed by Bhatia was found to be about 8 eV. This is much higher than the temperature found for all other observations, which is consistent with the fact that most of the lines observed in Bhatia’s experiment belong to higher ionization stages.

### 6.4 Line Intensities in the Spectra Observed by Lang and Sawyer [3]

**Fig. 4:**
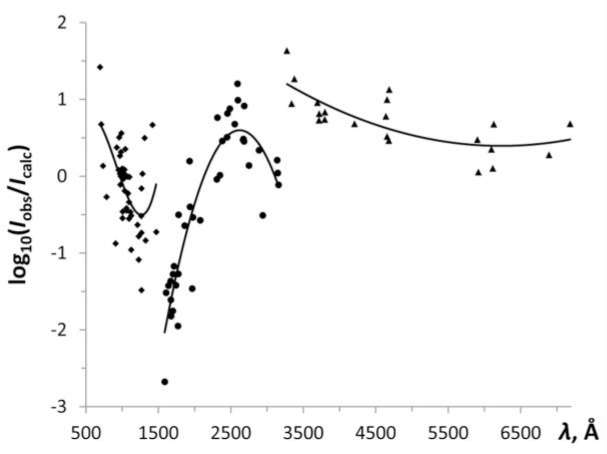
A logarithmic plot of the spectral sensitivity of experimental set-up of Lang and Sawyer [3] in three wavelength regions, 500 Å to 1500 Å, 1500 Å to 3200 Å, and 3200 Å to 7000 Å. The solid lines are polynomial least-squares fits to the data points (cubic for the first two regions, and quadratic for the last one).

As noted in Sec. 2.5, Lang and Sawyer [3] used three different spectrographs, a 1 m vacuum grating spectrograph and two prism spectrographs, to photograph the spectrum between 500 Å and 8000 Å. They did not specify the spectral regions in which each of these spectrographs was used. However, by making Boltzmann plots similar to those described in the previous section it was possible to determine the effective temperature of their light source, *T*_eff_ ≈ 2.5 eV, and divide the entire spectral range of their observations into three separate regions with distinctly different variation of spectral sensitivity. This division is illustrated in Fig. 4, which depicts the dependence of the logarithm of the ratio of observed and calculated intensities.

### 6.5 Line Intensities in the Spectra Observed by Wagatsuma [15]

By using a similar method as described above, I determined the effective excitation temperatures for the three sets of measurements presented by Wagatsuma. For the spectrum obtained with neon buffer gas, the effective temperature was *T*_eff_ ≈ 2.1 eV; for the Ar-He mixture, *T*_eff_ ≈ 2.0 eV; and for pure argon, *T*_eff_ ≈ 1.2 eV. Since the greatest number of lines was observed with neon buffer gas, I reduced all intensity measurements to *T*_eff_ = 2.1 eV and averaged all intensities observed in more than one set-up.

### 6.6 Reduction of Intensities to a Uniform Scale

After the intensities reported by different observers were corrected for variations of the response functions, as described above, they were reduced to a uniform scale by scaling them to the same effective excitation temperature T*. This temperature was chosen to be about 3 eV, since the most extensive line list given by Paschen and Campbell is described by a temperature close to that value (see Sec. 6.2). The scaling was made by multiplying the intensity values by a factor *f*_sc_exp[–*E_k_*(1/kT*–1/*kT*_eff_)], where the scaling coefficient *f*_sc_ was determined by adjusting the scale so that the mean ratio of intensities to those of Paschen and Campbell is equal to one. After that, intensities from multiple observations were averaged. The resulting values are reported in Table 1. About 70 % of lines have only one intensity measurement. Most of them are from Paschen and Campbell [2], while about 90 are from Bhatia [5], 22 from Lang and Sawyer [3], two from Wagatsuma [15], and one from Karlsson and Litzén [11]. For 181 lines, intensities have been determined by averaging two or more measurements. For 17 lines, relative intensities resulting from measurements of different authors differ by more than an order of magnitude. These highly volatile values, as well as five other intensities that may have been affected by blending with other lines, have been marked as unreliable in Table 1.

## 7. Transition Probabilities

Calculated or measured transition probabilities and radiative lifetimes of In II are given in more than 30 published articles. The complete list of these articles can be obtained from the NIST Atomic Transition Probability Bibliographic Database [36]. Here I will discuss only the articles that provide the most dependable data.

Becker *et al.* [37] measured the radiative lifetime of the metastable 5s5p ^3^P°_0_ level of a single laser-cooled ^115^In^+^ ion in a radiofrequency trap. The value they obtained, 0.195(8) s, corresponds to the probability of 5.13(21) s^−1^ for the hyperfine-induced transition to the ground state. These authors also measured the Landé factor of the 5s5p ^3^P°_0_ level, –0.000987(5). As well as the non-zero decay probability, the non-zero value of the Landé factor is caused by perturbations induced by the hyperfine interactions.

Ansbacher *et al.* [38] measured radiative lifetimes for 13 levels of In II using the beam-foil method with an account for cascade corrections. These measurements provided a base for the most accurate transition probability values of transitions originating from those levels. For two levels, 5s5d ^3^D_3_ and 5s4f ^3^F°_4_, there is only one branch of radiative decay to the 5s5p ^3^P°_2_ and 5s5d ^3^D_3_ levels, respectively, so the measured lifetimes directly yield the transition probabilities. For each of the other eight levels (5s6s ^1^S_0_, 5s6s ^3^S_1_, 5s5d ^1^D_2_, 5s5d ^3^D_1_, 5s5d ^3^D_2_, 5p^2 3^P_2_, 5p^2 1^D_2_, and 5p^2 1^S_0_) radiative decay is dominated by one transition. For these levels, the transition probability of the dominant decay branch could be accurately determined by subtracting from the total decay rate (i.e., inverse of the measured lifetime) the sum of the contributions of the other weak decay branches, which were accurately calculated by other authors. Uncertainties of thus determined transition probabilities vary between 3 % and 40 %.

Curtis *et al.* [39] made an isoelectronic comparison of measured and calculated radiative lifetimes of the 5s5p ^1^P°_1_ and 5s5p ^3^P°_1_ levels for Cd-like spectra of elements between Cd and Ho. Their semi-empirically corrected values for In II are probably accurate to within a few percent. They agree well with the best available measurements [38,40].

Martinez *et al.* [41] measured the branching fractions of transitions from the four 5s4f levels using an optically thin laser-produced plasma. From these data, they derived transition probabilities (*A*-values) for 10 transitions by normalizing them to the lifetimes measured by Ansbacher *et al.* [38] (for 5s4f ^1^F°_3_ and 5s4f ^3^F°_3,4_) and by Blagoev *et al.* [42] (for 5s4f ^3^F°_2_). For these *A*-values, they gave uncertainty values ranging from 10 % to 35 %. I have adopted their *A*-values that were normalized to the lifetimes measured by Ansbacher *et al.* Comparison of different measurements and accurate calculations shows that the lifetimes measured by Blagoev *et al.* [42] cannot be trusted. In most cases, they are too low by up to a factor of 2.

Jönsson and Andersson [43] made extensive relativistic multiconfiguration Dirac–Hartree–Fock calculations of oscillator strengths for electric-dipole transitions in In II. They gave weighted oscillator strengths (*gf*) calculated in both Babushkin and Coulomb gauges, and provided for each transition their calculated wave number. Comparison of the values in the two gauges provides a rough estimate of average uncertainty of their *gf* values, which is about 15 %. The given wave numbers allowed me to adjust their *gf* values by re-scaling them to the accurate Ritz wave numbers and convert to the *A*-values. Comparison of the resulting *A*-values with the accurate data discussed above shows that there is a strong dependence of the accuracy of the calculated *A*-values on the line strength *S*. It turned out that the *A*-values for *S* > 3, as calculated by Jönsson and Andersson, are accurate to within 8 %, while for weaker transitions the accuracy is much worse, 33 % on average. The agreement between the *gf* values in Babushkin and Coulomb gauges was used as an additional indicator of accuracy. I have decreased the assumed accuracy for transitions for which the *gf* values in the two gauges differ by more than 15 %.

Biémont and Zeippen [35] calculated the oscillator strengths for a large number of electric dipole transitions in In II using Cowan’s Hartree-Fock Relativistic method [30] modified to account for core-polarization effects by including a semi-empirically determined model potential. The radial parameters of the wave functions were determined in a least-squares fitting of the experimentally known energy levels. I have compared their results with an extended base of accurately determined *A*-values that included the accurate results from Jönsson and Andersson [43] in addition to other results discussed above. For strong transitions with *S* > 1.25, calculations of Biémont and Zeippen agree with other accurate results with an average deviation of 18 %. For transitions with *S* between 0.2 and 1.25, the average deviation is about 30 %. For weaker transitions, the agreement is much worse, often only to an order of magnitude.

Lavín and Martin [44] employed the relativistic quantum defect orbital (RQDO) approach with an account for core polarization to calculate the oscillator strengths of the 5s5p ^3^P – 5s*n*d ^3^D (*n* = 5–9) and 5s5p ^3^P – 5s*n*s ^3^S (*n* = 6–10) transitions in In II and similar transitions in a few neighboring isoelectronic spectra. Where the scope of their calculations intersects the scope of the other works considered above [38,35,43], their results agree to about 25 % with those references with no discernible correlation with the line strength.

The works discussed above provided an extensive database for comparison with my semi-empirical Cowan-code calculations. It is well known that Cowan-code calculations of transition rates suffer from cancellation effects. To check whether these effects are significant, the Cowan code provides the value of the cancellation factor in the output for each transition. The cancellation factor is computed as follows. The line strength is computed as an expansion over contributions from each basis state. The sum of positive contributions *S*^+^ and the sum of negative contributions *S*^−^ are computed separately, and the cancellation factor is calculated as CF = (*S*^+^ + *S*^−^)/(*S*^+^ + |*S*^−^|). Since each of *S*^+^ and *S*^−^ are calculated with a finite accuracy, the values of CF close to zero indicate a significant loss of accuracy in the final line strength. Of course, this is only a qualitative criterion. Different researchers adopt different threshold values of CF below which they consider the results to be adversely affected by cancellations. In the case of the In II calculations, my comparisons show that there is no correlation between the accuracy of line-strength calculations and CF for |CF| greater than 0.1. However, the values of |CF| < 0.1 generally indicate that the resulting line strength can be in error by an order of magnitude or more.

Considering only transitions unaffected by cancellations, I found that, similar to several other calculation methods discussed above, the accuracy of Cowan-code calculations strongly depends on the line strength. For strongest transitions with *S* > 80, the results of Cowan’s code agree with other, more accurate calculations or measurements within 9 % on average. For strong transitions with *S* between 2.5 and 80, the accuracy is about 23 % on average. For weaker transitions with *S* < 2.5, the average accuracy is about 30 %. I have discarded most of the *A*-values from the Cowan code calculations that were adversely affected by cancellation effects (i.e., those that have CF < 0.1). Only a few of such unreliable *A*-values are included in Table 1 for transitions that have observed intensities roughly agreeing with those calculated using these *A*-values in the assumption of LTE.

Comparisons of radiative lifetimes measured by different authors with calculations were given in many papers; see, for example, Biémont and Zeippen [35]. These comparisons show that the various measurements strongly disagree with each other for many energy levels. It can be seen that the lifetimes measured by Ansbacher *et al.* [38] with an account for multi-exponential decay are in much better agreement with theory than the other measurements. Some of the lifetimes measured by other authors may be significantly affected by cascades from higher levels. For example, there is a large disagreement between theory and measurements for the 5s5f ^3^F°_4_ level. For this level, Blagoev *et al.* [42] measured a lifetime of 19(1) ns, in agreement with an earlier beam-foil measurement by Andersen and Sørensen [45], 21(2) ns. Different calculations yield consistently lower values, 11.8 ns [43], 10.0 ns [35], or 9.5 ns (this work). As follows from my calculations, the measured lifetime probably corresponds to the cascade from 5s6g, for which my calculations yield a lifetime of 21.3 ns. Similarly, for the 5s5f ^3^F°_3_ level, Andersen and Sørensen [45] reported a lifetime of 21(2) ns, while three different calculations yield 11.7 ns [43], 9.9 ns [35], and 9.4 ns (this work). Similar to the 5s5f ^3^F°_4_ level, the measured 5s5f ^3^F°_3_ lifetime appears to correspond to the cascade from 5s6g.

The critically evaluated transition probabilities with assessed uncertainties are compiled in Table 1. The uncertainties are denoted by a letter code explained in Table 3.

It should be noted that the transition probability values resulting from theoretical calculations do not follow a normal statistical distribution. For example, for normal statistics the uncertainty of ±10 % (at the one standard deviation level) would imply that the true value should be within 20 % of the measured or calculated one with a probability of 95 %, and a deviation of 30 % has an extremely small probability. However, in quantum mechanics calculations it is often observed that the bulk of results for *A*-values lie within certain limits from true values, but there are some *A*-values (usually for weak transitions) that deviate grossly (by an order of magnitude or more) from the true values. This is the reason for using the above letter codes rather than the usual numerical specification of uncertainties. The numerical uncertainty values given in Table 3 should be used with caution, since there may be a significant number of outliers one would not expect to have in normal statistics. The logarithms of the *A*-values, as well as the values of log (*gf*), have statistical distributions much closer to normal.

## 8. Conclusions

In the present study, a comprehensive list of the best measured wavelengths of 650 observed lines has been compiled. Uncertainties of all available wavelength measurements in the In II spectrum have been analyzed, and the existing inconsistencies have been resolved. From this line list with assigned uncertainties, a set of 173 optimized energy levels that fits all observed wavelengths has been derived. An additional 21 levels have been determined semi-empirically from series formulas and from parametric fitting. Percentage compositions of the levels and radiative transition rates have been calculated in the parametric fitting procedure. An improved value of the ionization limit of In II has been determined by fitting quantum-defect and polarization formulas for several series of levels. The line intensities observed by different authors have been analyzed and converted to a uniform scale. A set of 528 recommended values of radiative transition rates has been critically compiled, and uncertainties of these rates have been estimated. Of these transition rates, 353 are associated with observed lines. From the observed separations of levels in the 5s*n*g and 5s*n*h series of In II, the hfs splitting of the ground state of ^115^In III has been found to be 3.56(3) cm^−1^.

## Figures and Tables

**Table 1:** Observed and predicted spectral lines of In II

*λ*_obs_^a^ (Å)	*σ*_obs_ (cm^−1^)	*λ*_Ritz_^b^ (Å)	Δ*λ*_obs-Ritz_^c^ (Å)	*I*_obs_^d^ (arb.u.)	*A*^e^ (s^−1^)	Type^f^		Transition				Notes^g^	Line Ref.^h^	TP Ref.^i^
680.28(3)(1)	146998	680.20(5)	0.08	170				5s^2^	^1^S_0_	5s12p	^1^P°_1_		B69	
686.54(3)(1)	145658	686.4185(7)	0.12	170				5s^2^	^1^S_0_	5s11p	^1^P°_1_		B69	
695.83(11)(24)	143710	695.5888(4)	0.24	180	6e+5	E		5s^2^	^1^S_0_	5s10p	^1^P°_1_	1	L31	TW
695.86(3)(0)	143707	695.8626(4)	0.00	70	1.0e+5	E		5s^2^	^1^S_0_	5s10p	3P°_1_	1	B69	TW
710.09(11)(24)	140830	710.00036(18)	0.09	95	1.5e+6	E		5s^2^	^1^S_0_	5s9p	^1^P°_1_	1	L31	TW
710.55(3)(0)	140736	710.55737(21)	−0.01	47	3.1e+5	E		5s^2^	^1^S_0_	5s9p	3P°_1_	1	B69	TW
734.78(3)(0)	136095	734.7704(5)	0.01	230	3.6e+7	E		5s^2^	^1^S_0_	5s8p	^1^P°_1_		B69	B99
736.04(3)(0)	135862	736.0430(3)	0.00	210	8e+5	E		5s^2^	^1^S_0_	5s8p	3P°_1_	1	B69	TW
783.98(3)(0)	127554	783.86296(6)	0.12	440				5s^2^	^1^S_0_	5s7p	^1^P°_1_		B69	
787.45(3)(0)	126992	787.43326(7)	0.02	1100	1.7e+6	E		5s^2^	^1^S_0_	5s7p	3P°_1_	1	B69	TW
910.90(3)(1)	109782	910.91090(7)	−0.01	2700	7e+6	E		5s^2^	^1^S_0_	5s6p	^1^P°_1_		B69	B99
927.27(3)(1)	107843	927.28257(5)	−0.01	2500	4.9e+6	D+		5s^2^	^1^S_0_	5s6p	3P°_1_		B69	TW
		933.7684(19)			1.5e+6	D+		5s5p	^3^P°_1_	5s19s	^3^S_1_			TW
		935.9123(19)			1.9e+6	D+		5s5p	^3^P°_1_	5s18s	^3^S_1_			TW
		936.5492(6)			1.5e+6	D+		5s5p	^3^P°_0_	5s15s	^3^S_1_			TW
937.87(3)(1)	106625	937.9023(23)	−0.03	610*				5s5p	^3^P°_1_	5s16d	^3^D_2_		B69	
937.87(3)(1)	106625	937.9269(21)	−0.06	610*				5s5p	^3^P°_1_	5s16d	^3^D_1_		B69	
		938.5571(14)			2.5e+6	D+		5s5p	^3^P°_1_	5s17s	^3^S_1_			TW
940.60(3)(1)	106315	940.6123(18)	−0.01	970				5s5p	^3^P°_0_	5s13d	^3^D_1_		B69	
941.87(3)(1)	106172	941.8556(15)	0.01	760*	3.2e+6	D+		5s5p	^3^P°_1_	5s16s	^3^S_1_		B69	TW
941.87(3)(1)	106172	941.9310(6)	−0.06	760*	2.0e+6	D+		5s5p	^3^P°_0_	5s14s	^3^S_1_		B69	TW
944.81(3)(1)	105841	944.7713(20)	0.04	1200				5s5p	^3^P°_1_	5s14d	^1^D_2_		B69	
		946.0705(6)			4.2e+6	D+		5s5p	^3^P°_1_	5s15s	^3^S_1_			TW
947.41(3)(1)	105551	947.3507(7)	0.06	390				5s5p	^3^P°_0_	5s12d	^3^D_1_		B69	
949.17(3)(1)	105355	949.1393(5)	0.03	910	2.9e+6	D+		5s5p	^3^P°_0_	5s13s	^3^S_1_		B69	TW
950.19(3)(1)	105242	950.1936(16)	0.00	660				5s5p	^3^P°_1_	5s13d	^3^D_2_		B69	
950.32(3)(1)	105228	950.2168(18)	0.10	670				5s5p	^3^P°_1_	5s13d	^3^D_1_		B69	
951.27(3)(1)	105123	951.2487(20)	0.02	1200				5s5p	^3^P°_1_	5s14s	^1^S_0_		B69	
951.59(3)(1)	105087	951.5626(6)	0.03	480	5.8e+6	D+		5s5p	^3^P°_1_	5s14s	^3^S_1_		B69	TW
		955.8926(20)			2.4e+6	D+		5s5p	^3^P°_2_	5s19s	^3^S_1_			TW
956.62(3)(1)	104535	956.5864(13)	0.03	750*				5s5p	^3^P°_1_	5s12d	^1^D_2_		B69	
956.62(3)(1)	104535	956.6036(7)	0.02	750*				5s5p	^3^P°_0_	5s11d	^3^D_1_		B69	
957.28(11)(16)	104463	957.0687(15)	0.21	780*				5s5p	^3^P°_1_	5s12d	^3^D_2_		L31	
957.28(11)(16)	104463	957.0941(7)	0.19	780*				5s5p	^3^P°_1_	5s12d	^3^D_1_		L31	
957.55(3)(1)	104433	957.5845(18)	−0.03	580*				5s5p	^3^P°_2_	5s17d	^3^D_3_		B69	
957.55(3)(1)	104433	957.591(4)	−0.04	580*				5s5p	^3^P°_2_	5s17d	^3^D_1_		B69	
957.55(3)(1)	104433	957.591(4)	−0.04	580*				5s5p	^3^P°_2_	5s17d	^3^D_2_		B69	
		958.1394(20)			3.1e+6	D+		5s5p	^3^P°_2_	5s18s	^3^S_1_			TW
959.06(11)(15)	104269	958.9196(6)	0.14	790	9e+6	D+		5s5p	^3^P°_1_	5s13s	^3^S_1_		L31	TW
		959.1299(3)			4.9e+6	D+		5s5p	^3^P°_0_	5s12s	^3^S_1_			TW
960.30(3)(1)	104134	960.2178(18)	0.08	780*				5s5p	^3^P°_2_	5s16d	^3^D_3_		B69	
960.30(3)(1)	104134	960.2251(24)	0.07	780*				5s5p	^3^P°_2_	5s16d	^3^D_2_		B69	
960.30(3)(1)	104134	960.2509(22)	0.05	780*				5s5p	^3^P°_2_	5s16d	^3^D_1_		B69	
960.98(3)(1)	104060	960.9115(14)	0.07	310	3.9e+6	D+		5s5p	^3^P°_2_	5s17s	^3^S_1_		B69	TW
964.33(3)(1)	103699	964.3693(16)	−0.04	1100	5.0e+6	D+		5s5p	^3^P°_2_	5s16s	^3^S_1_		B69	TW
966.64(3)(1)	103451	966.5136(8)	0.13	1700*				5s5p	^3^P°_1_	5s11d	^3^D_2_		B69	
966.64(3)(1)	103451	966.5392(7)	0.10	1700*				5s5p	^3^P°_1_	5s11d	^3^D_1_		B69	
		968.7886(7)			7e+6	D+		5s5p	^3^P°_2_	5s15s	^3^S_1_			TW
969.07(3)(1)	103192	969.1183(3)	−0.05	1100	1.4e+7	D+		5s5p	^3^P°_1_	5s12s	^3^S_1_		B69	TW
969.93(3)(1)	103100	969.8663(3)	0.06	1600	4.6e+7	D+		5s5p	^3^P°_0_	5s10d	^3^D_1_		B69	TW
972.93(3)(1)	102782	973.0797(19)	−0.15	850*				5s5p	^3^P°_2_	5s13d	^3^D_3_		B69	
972.93(3)(1)	102782	973.1126(17)	−0.18	850*				5s5p	^3^P°_2_	5s13d	^3^D_2_		B69	
972.93(3)(1)	102782	973.1369(19)	−0.21	850*				5s5p	^3^P°_2_	5s13d	^3^D_1_		B69	
973.48(6)(1)	102724	973.5772(3)	−0.10	1400*bl*	1.6e+7	D+		5s5p	^3^P°_0_	5s11s	^3^S_1_		B69	TW
974.54(3)(1)	102613	974.5485(7)	−0.01	390	9e+6	D+		5s5p	^3^P°_2_	5s14s	^3^S_1_		B69	TW
978.99(3)(1)	102146	978.9623(6)	0.03	810				5s5p	^3^P°_1_	5s10d	^1^D_2_		B69	
980.21(3)(1)	102019	980.0355(5)	0.17	2800*	6e+7	D+		5s5p	^3^P°_1_	5s10d	^3^D_2_		B69	TW
980.21(3)(1)	102019	980.0808(3)	0.13	2800*	3.3e+7	D+		5s5p	^3^P°_1_	5s10d	^3^D_1_		B69	TW
980.21(3)(1)	102019	980.2863(18)	−0.08	2800*				5s5p	^3^P°_2_	5s12d	^3^D_3_		B69	
980.43(3)(1)	101996	980.3246(16)	0.11	1900				5s5p	^3^P°_2_	5s12d	^3^D_2_		B69	
982.31(3)(1)	101801	982.2666(6)	0.04	800	1.3e+7	D+		5s5p	^3^P°_2_	5s13s	^3^S_1_		B69	TW
983.05(3)(1)	101724	982.9394(4)	0.11	790	8e+5	E		5s5p	^3^P°_1_	5s11s	^1^S_0_	1	B69	TW
984.05(3)(1)	101621	983.8703(3)	0.18	1200	4.5e+7	D+		5s5p	^3^P°_1_	5s11s	^3^S_1_		B69	TW
990.19(3)(1)	100991	989.87874(24)	0.31	2900*	2.0e+7	C		5s5p	^3^P°_0_	5s9d	^3^D_1_		B69	L94
990.19(3)(1)	100991	990.1839(13)	0.01	2900*				5s5p	^3^P°_2_	5s11d	^3^D_3_		B69	
990.19(3)(1)	100991	990.2364(9)	−0.05	2900*				5s5p	^3^P°_2_	5s11d	^3^D_2_		B69	
992.97(3)(1)	100708	992.9707(3)	0.00	770	2.2e+7	D+		5s5p	^3^P°_2_	5s12s	^3^S_1_		B69	TW
		995.69532(21)			4.5e+6	C		5s5p	^3^P°_0_	5s10s	^3^S_1_			L94
1000.45(3)(1)	99955	1000.4539(3)	0.00	2700	2.8e+7	C		5s5p	^3^P°_1_	5s9d	^3^D_2_		B69	L94
1000.54(3)(1)	99946	1000.52139(25)	0.02	2700	1.3e+7	C		5s5p	^3^P°_1_	5s9d	^3^D_1_		B69	L94
1004.35(3)(1)	99567	1004.3621(4)	−0.01	2000*	8e+7	D+		5s5p	^3^P°_2_	5s10d	^3^D_3_		B69	TW
1004.35(3)(1)	99567	1004.4351(5)	−0.09	2000*	1.9e+7	D+		5s5p	^3^P°_2_	5s10d	^3^D_2_		B69	TW
1004.95(3)(1)	99507	1004.9617(3)	−0.01	860				5s5p	^3^P°_1_	5s10s	^1^S_0_		B69	
1006.48(3)(1)	99356	1006.46409(21)	0.02	1200	1.3e+7	C		5s5p	^3^P°_1_	5s10s	^3^S_1_		B69	L94
1008.41(3)(1)	99166	1008.4637(3)	−0.05	1200	7e+7	D+		5s5p	^3^P°_2_	5s11s	^3^S_1_		B69	TW
1022.44(3)(1)	97805	1022.4297(3)	0.01	2300	3.4e+7	C		5s5p	^3^P°_0_	5s8d	^3^D_1_		B69	L94
		1025.7828(15)			3.2e+7	C		5s5p	^3^P°_2_	5s9d	^3^D_3_			L94
		1025.8940(3)			6.3e+6	C		5s5p	^3^P°_2_	5s9d	^3^D_2_			L94
1032.21(3)(1)	96880	1032.21472(22)	0.00	2100*	2.1e+7	C		5s5p	^3^P°_2_	5s10s	^3^S_1_		B69	L94
1032.21(3)(1)	96880	1032.40663(23)	−0.20	2100*	6.3e+6	C		5s5p	^3^P°_0_	5s9s	^3^S_1_		B69	L94
1033.69(3)(1)	96741	1033.6766(3)	0.01	3100	4.5e+7	C		5s5p	^3^P°_1_	5s8d	^3^D_2_		B69	L94
		1033.7878(3)			2.5e+7	C		5s5p	^3^P°_1_	5s8d	^3^D_1_			L94
1043.89(3)(1)	95796	1043.98874(23)	−0.10	3300	1.8e+7	C		5s5p	^3^P°_1_	5s9s	^3^S_1_		B69	L94
1060.68(3)(1)	94279	1060.6727(6)	0.01	3700	5.5e+7	C		5s5p	^3^P°_2_	5s8d	^3^D_3_		B69	L94
1060.92(3)(1)	94258	1060.8573(3)	0.06	1700	1.2e+7	C		5s5p	^3^P°_2_	5s8d	^3^D_2_		B69	L94
		1060.9745(3)			9.9e+5	C		5s5p	^3^P°_2_	5s8d	^3^D_1_			L94
1062.75(3)(1)	94096	1062.7839(8)	−0.03	990*i*				5s5p	^3^P°_2_	5s_1/2,_*_F_*_=4_6g			B69	
1071.93(11)(13)	93290	1071.72178(24)	0.21	3100	2.9e+7	C		5s5p	^3^P°_2_	5s9s	^3^S_1_		L31	L94
1081.66(11)(13)	92450	1081.66000(12)	0.00	5000	4.6e+7	C		5s5p	^3^P°_0_	5s7d	^3^D_1_		L31	J07
		1086.36913(11)			1.6e+7	E		5s5p	^3^P°_1_	5s7d	^1^D_2_			B99
1094.18(3)(1)	91393	1094.17047(11)	0.01	6200	6.0e+7	C		5s5p	^3^P°_1_	5s7d	^3^D_2_		B69	J07
1094.38(3)(1)	91376	1094.38039(12)	0.00	2500	3.3e+7	C		5s5p	^3^P°_1_	5s7d	^3^D_1_		B69	J07
1101.38(3)(1)	90795.2	1101.36385(9)	0.02	2200	1.3e+7	C		5s5p	^3^P°_0_	5s8s	^3^S_1_		B69	J07
1114.55(11)(12)	89722	1114.55472(9)	0.00	1900	3.8e+7	C		5s5p	^3^P°_1_	5s8s	^3^S_1_		L31	J07
1116.42(3)(1)	89572.0	1116.43191(11)	−0.01	2600	1.5e+5	D+		5s5p	^3^P°_2_	5s7d	^1^D_2_		B69	TW
1124.39(11)(12)	88937	1124.32011(10)	0.07	4000	7.3e+7	C		5s5p	^3^P°_2_	5s7d	^3^D_3_		L31	J07
1124.70(11)(12)	88913	1124.67263(10)	0.03	2000	1.8e+7	C		5s5p	^3^P°_2_	5s7d	^3^D_2_		L31	J07
		1124.89442(12)			2.0e+6	C		5s5p	^3^P°_2_	5s7d	^3^D_1_			J07
1146.14(11)(11)	87249	1146.22042(8)	−0.08	1100	6.4e+7	C		5s5p	^3^P°_2_	5s8s	^3^S_1_		L31	J07
1161.06(3)(1)	86128.2	1161.043(3)	0.02	1200*				5s5p	^1^P°_1_	5s14d	^3^D_2_		B69	
1161.06(3)(1)	86128.2	1161.0790(22)	−0.02	1200*				5s5p	^1^P°_1_	5s14d	^3^D_1_		B69	
1162.32(3)(1)	86034.8	1162.267(4)	0.05	1800				5s5p	^1^P°_1_	5s15s	^1^S_0_		B69	
1162.59(3)(1)	86014.8	1162.6230(10)	−0.03	1300				5s5p	^1^P°_1_	5s15s	^3^S_1_		B69	
1179.24(3)(1)	84800.4	1179.2767(23)	−0.04	1500				5s5p	^1^P°_1_	5s12d	^3^D_2_		B69	
1193.68(3)(1)	83774.5	1193.6494(12)	0.03	2100				5s5p	^1^P°_1_	5s11d	^3^D_2_		B69	
		1200.18680(11)			2.3e+6	D		5s5p	^3^P°_1_	5s6d	^1^D_2_			J07
1212.63(3)(1)	82465.4	1212.61017(9)	0.02	8100	1.3e+8	C		5s5p	^3^P°_0_	5s6d	^3^D_1_		B69	J07
1214.44(3)(1)	82342.5	1214.3416(8)	0.10	1900*	3.0e+5	D+		5s5p	^1^P°_1_	5s10d	^3^D_2_		B69	TW
1214.44(3)(1)	82342.5	1214.4110(5)	0.03	1900*	1.7e+5	D+		5s5p	^1^P°_1_	5s10d	^3^D_1_		B69	TW
1218.76(3)(1)	82050.6	1218.8031(5)	−0.04	1600	1.5e+8	D+		5s5p	^1^P°_1_	5s11s	^1^S_0_		B69	TW
1228.24(11)(10)	81417	1228.10697(9)	0.13	8300	1.7e+8	C		5s5p	^3^P°_1_	5s6d	^3^D_2_		L31	J07
1228.62(11)(10)	81392	1228.61975(9)	0.00	5500	9.4e+7	C		5s5p	^3^P°_1_	5s6d	^3^D_1_		L31	J07
1236.99(3)(1)	80841.4	1236.98554(10)	0.00	1700	5e+5	E		5s5p	^3^P°_2_	5s6d	^1^D_2_		B69	B99
1243.10(3)(1)	80444.1	1243.0800(10)	0.02	2600	1.3e+8	D+		5s5p	^1^P°_1_	5s9d	^1^D_2_		B69	TW
1245.91(3)(1)	80262.6	1245.9518(4)	−0.04	1400	8e+4	D+		5s5p	^1^P°_1_	5s9d	^3^D_1_		B69	TW
1249.67(3)(1)	80021.1	1249.6522(3)	0.02	2800	5e+6	E		5s5p	^3^P°_1_	5s7s	^1^S_0_		B69	B99
1263.16(3)(1)	79166.5	1263.15982(19)	0.00	3700	3.0e+7	C		5s5p	^3^P°_0_	5s7s	^3^S_1_		B69	J07
1265.96(11)(10)	78991	1265.80804(11)	0.15	5600	2.1e+8	C+		5s5p	^3^P°_2_	5s6d	^3^D_3_		L31	J07
1266.67(3)(1)	78947.2	1266.66524(7)	0.00	4900	5.2e+7	C		5s5p	^3^P°_2_	5s6d	^3^D_2_		B69	J07
1267.19(11)(9)	78915	1267.21073(8)	−0.02	1400	5.8e+6	C		5s5p	^3^P°_2_	5s6d	^3^D_1_		L31	J07
1280.49(11)(9)	78095	1280.54157(20)	−0.05	28000	8.9e+7	C		5s5p	^3^P°_1_	5s7s	^3^S_1_		L31	J07
		1283.0555(5)			8e+6	D		5s5p	^3^P°_1_	5p^2^	^1^S_0_			J07
1292.50(3)(1)	77369.4	1292.4937(7)	0.01	2000	1.7e+8	D+		5s5p	^1^P°_1_	5s8d	^1^D_2_		B69	TW
1297.78(3)(1)	77054.7	1297.7896(5)	−0.01	1000				5s5p	^1^P°_1_	5s8d	^3^D_2_		B69	
1309.82(3)(1)	76346.4	1309.7791(5)	0.04	2200	2.4e+7	E		5s5p	^1^P°_1_	5s9s	^1^S_0_	1	B69	TW
1314.12(3)(1)	76096.6	1314.0862(4)	0.03	3	1.1e+5	D+		5s5p	^1^P°_1_	5s9s	^3^S_1_		B69	TW
1322.51(3)(1)	75613.8	1322.51892(20)	−0.01	4200	1.5e+8	C		5s5p	^3^P°_2_	5s7s	^3^S_1_		B69	J07
		1381.94502(20)			1.8e+8	D		5s5p	^1^P°_1_	5s7d	^1^D_2_			J07
1394.92(3)(0)	71688.7	1394.93473(23)	−0.01	190	2.4e+5	D+		5s5p	^1^P°_1_	5s7d	^3^D_1_		B69	TW
1417.75(3)(0)	70534.3	1417.74920(22)	0.00	410	3.6e+6	E		5s5p	^3^P°_1_	5p^2^	^1^D_2_		B69	B99
1418.13(3)(0)	70515.4	1418.1212(3)	0.01	2000	1.2e+8	D		5s5p	^1^P°_1_	5s8s	^1^S_0_		B69	J07
1469.49(11)(8)	68051	1469.38544(22)	0.10	2300	2.5e+7	D		5s5p	^3^P°_2_	5p^2^	^1^D_2_		L31	J07
1571.53(3)(0)	63632.3	1571.52648(23)	0.00	260000	5.2e+8	B		5s5p	^1^P°_1_	5s6d	^1^D_2_		B69	J07
1586.37(11)(7)	63037	1586.33100(24)	0.04	850000	1.28e+9	B+		5s^2^	^1^S_0_	5s5p	^1^P°_1_		L31	CMEF00
1607.32(3)(1)	62215.4	1607.3371(3)	−0.02	260000	3.2e+8	B		5s5p	^3^P°1	5p^2^	^3^P_2_		B69	J07
1619.80(3)(1)	61736.0	1619.74357(23)	0.06	17	2.7e+5	E		5s5p	^1^P°_1_	5s6d	^3^D_2_	1	B69	TW
1620.60(3)(1)	61705.5	1620.63567(23)	−0.04	15	5.2e+5	D+		5s5p	^1^P°_1_	5s6d	^3^D_1_		B69	TW
1640.05(3)(1)	60973.8	1640.060(4)	−0.01	180000	3.7e+8	C		5s5p	^3^P°_0_	5p^2^	^3^P_1_		B69	J07
		1657.4320(5)		240000	1.36e+9	B		5s5p	^1^P°_1_	5s7s	^1^S_0_		W96	J07
1669.46(3)(1)	59899.6	1669.482(4)	−0.02	110000	3.5e+8	C		5s5p	^3^P°_1_	5p^2^	^3^P_1_		B69	J07
1671.87(3)(1)	59813.3	1671.8850(11)	−0.02	120000	7.3e+8	B		5s5p	^3^P°_0_	5s5d	^3^D_1_		B69	A86c
1674.02(3)(1)	59736.4	1674.0317(3)	−0.01	130000	9.5e+8	B		5s5p	^3^P°_2_	5p^2^	^3^P_2_		B69	A86c,J07
1699.97(3)(1)	58824.6	1699.9833(7)	−0.01	200000	8.6e+8	B		5s5p	^3^P°_1_	5s5d	^3^D_2_		B69	A86c
1702.45(3)(1)	58738.9	1702.4714(12)	−0.02	120000	4.1e+8	B		5s5p	^3^P°_1_	5s5d	^3^D_1_		B69	J07
1716.53(3)(1)	58257.1	1716.518(6)	0.01	110000	1.1e+9	C		5s5p	^3^P°_1_	5p^2^	^3^P_0_		B69	J07
1716.70(3)(1)	58251.3	1716.7092(9)	−0.01	19000	1.08e+9	C+		5s5p	^1^P°_1_	5p^2^	^1^S_0_		B69	A86c
1741.53(3)(1)	57420.8	1741.549(4)	−0.02	95000	4.9e+8	B		5s5p	^3^P°_2_	5p^2^	^3^P_1_		B69	J07
1770.56(3)(1)	56479.3	1770.5652(4)	−0.01	140000	1.06e+9	A		5s5p	^3^P°_2_	5s5d	^3^D_3_		B69	A86
1774.75(3)(1)	56346.0	1774.7667(8)	−0.02	58000	2.34e+8	B		5s5p	^3^P°_2_	5s5d	^3^D_2_		B69	J07
1777.46(3)(1)	56260.1	1777.4787(13)	−0.02	27000	1.6e+7	C		5s5p	^3^P°_2_	5s5d	^3^D_1_		B69	J07
1842.36(3)(1)	54278.2	1842.3720(3)	−0.01	26000	3.7e+6	D		5s5p	^3^P°_1_	5s5d	^1^D_2_		B69	J07
1862.89(3)(1)	53680.0	1862.9040(4)	−0.01	4900	4.7e+6	D		5s5p	^3^P°_1_	5s6s	^1^S_0_		B69	J07
1930.5331(4)	51799.164	1930.5325(3)	0.0006	25000	1.1e+7	D+		5s5p	^3^P°_2_	5s5d	^1^D_2_		K01c	J07
1932.14(9)(1)	51756.1	1932.000(5)	0.14	590				5s6s	^3^S_1_	5s11p	^1^P°_1_		B69	
1936.1876(3)	51647.888	1936.18758(18)	0.0000	20000	9.6e+7	C		5s5p	^3^P°_0_	5s6s	^3^S_1_		K01c	J07
1953.34(9)(1)	51194.4	1953.434(5)	−0.09	300				5s6s	^3^S_1_	5s8f	^3^F°_2_		B69	
1965.98(9)(1)	50865.2	1966.063(5)	−0.08	720				5s5d	^1^D_2_	5s11f	^1^F°_3_	2	B69	
1966.7087(3)	50846.372	1966.70862(24)	0.0001	92000	1.27e+9	B+		5s5p	^1^P°_1_	5p^2^	^1^D_2_		K01c	A86c
1977.32788(23)	50573.302	1977.32789(19)	−0.00001	32000	2.7e+8	B		5s5p	^3^P°_1_	5s6s	^3^S_1_		K01c	J07
1997.03(9)(1)	50074.4	1996.968(5)	0.06	240	1.5e+7	D+		5s5d	^1^D_2_	5s10f	^1^F°_3_		B69	TW
1998.04(9)(1)	50049.0	1997.921(5)	0.12	140				5s5d	^1^D_2_	5s10f	^3^F°_3_		B69	
2008.12(9)(1)	49781.7	2008.082(3)	0.04	140				5s6s	^3^S_1_	5s10p	^3^P°_1_		B69	
2039.79(9)(1)	49008.9	2039.748(5)	0.04	330	4.0e+7	D+		5s5d	^1^D_2_	5s9f	^1^F°_3_		B69	TW
2040.81(9)(1)	48984.4	2040.7532(16)	0.06	210*				5s5d	^1^D_2_	5s9f	^3^F°_2_		B69	
2040.81(9)(1)	48984.4	2040.834(5)	−0.02	210*				5s5d	^1^D_2_	5s9f	^3^F°_3_		B69	
2054.76(9)(1)	48651.9	2054.692(6)	0.07	210				5s6s	^1^S_0_	5s11p	^1^P°_1_		B69	
2055.72(9)(1)	48629.2	2055.872(7)	−0.15	400				5s6s	^1^S_0_	5s11p	^3^P°_1_	2	B69	
2078.57182(13)	48094.629	2078.57186(13)	−0.00004	76000	4.2e+8	B		5s5p	^3^P°_2_	5s6s	^3^S_1_		K01c	A86c,J07
2080.30(9)(1)	48054.7	2080.274(6)	0.03	330				5s5d	^1^D_2_	5s11p	^1^P°_1_		B69	
2081.51(9)(1)	48026.7	2081.483(7)	0.03	300				5s5d	^1^D_2_	5s11p	^3^P°_1_		B69	
2103.891(18)	47515.9	2103.886(5)	0.005	1700	1.0e+7	E		5s5d	^1^D_2_	5s8f	^1^F°_3_	1	P38P	TW
2105.10(9)(1)	47488.6	2105.076(4)	0.02	190*				5s5d	^1^D_2_	5s8f	^3^F°_3_		B69	
2105.10(9)(1)	47488.6	2105.156(6)	−0.06	190*				5s5d	^1^D_2_	5s8f	^3^F°_2_		B69	
2135.64(9)(1)	46809.6	2135.5898(19)	0.05	160	3.5e+5	E		5s6s	^3^S_1_	5s9p	^3^P°_1_	1	B69	TW
2139.150(18)	46732.8	2139.146(4)	0.004	540				5s6s	^1^S_0_	5s10p	^1^P°_1_		P38P	
2141.68(9)(1)	46677.6	2141.739(4)	−0.06	170				5s6s	^1^S_0_	5s10p	^3^P°_1_		B69	
2166.876(19)	46134.9	2166.888(4)	−0.012	990	1.2e+7	D+		5s5d	^1^D_2_	5s10p	^1^P°_1_		P38P	TW
2169.548(19)	46078.1	2169.548(4)	0.000	560	1.7e+6	D+		5s5d	^1^D_2_	5s10p	^3^P°_1_		P38P	TW
2192.65(9)(0)	45592.7	2192.591(7)	0.06	70	3.8e+6	D+		5s5d	^3^D_1_	5s10f	^3^F°_2_		B69	TW
2195.668(19)	45530.0	2195.684(6)	−0.016	450				5s5d	^3^D_2_	5s10f	^1^F°_3_		P38P	
		2196.734(7)			7e+5	D+		5s5d	^3^D_2_	5s10f	^3^F°_2_			TW
		2196.837(7)			4.0e+6	D+		5s5d	^3^D_2_	5s10f	^3^F°_3_			TW
2197.27(9)(0)	45496.8	2197.441(10)	−0.17	97*i*				5s5d	^3^D_3_	5s_1/2,_*_F_*_=4_10h			B69	
2202.19(9)(0)	45395.2	2202.152(6)	0.04	130				5s5d	^3^D_3_	5s10f	^1^F°_3_		B69	
2202.67(9)(0)	45385.3	2202.756(7)	−0.09	74	4.4e+6	D+		5s5d	^3^D_3_	5s10f	^3^F°_4_		B69	TW
2203.27(9)(0)	45372.9	2203.311(7)	−0.04	80	4.9e+5	D+		5s5d	^3^D_3_	5s10f	^3^F°_3_		B69	TW
2205.279(19)	45331.6	2205.292(4)	−0.013	1000	1.8e+7	D+		5s5d	^1^D_2_	5s7f	^1^F°_3_		P38P	TW
2206.720(19)	45302.0	2206.7303(23)	−0.010	440				5s5d	^1^D_2_	5s7f	^3^F°_3_		P38P	
2245.31(9)(0)	44523.5	2245.208(3)	0.10	170	8e+6	D+		5s5d	^3^D_1_	5s9f	^3^F°_2_		B69	TW
2248.3180(15)	44463.9	2248.330(7)	−0.012	110				5s5d	^3^D_2_	5s9f	^1^F°_3_		P38P	
2249.61(7)	44438.3	2249.5515(23)	0.06	150bl*	1.5e+6	D+		5s5d	^3^D_2_	5s9f	^3^F°_2_		P38P	TW
2249.61(7)	44438.3	2249.650(6)	−0.04	150bl*	8e+6	D+		5s5d	^3^D_2_	5s9f	^3^F°_3_		P38P	TW
2255.787(15)	44316.7	2255.805(7)	−0.018	300	9e+6	D+		5s5d	^3^D_3_	5s9f	^3^F°_4_	2	P38P	TW
2256.14(9)(0)	44309.8	2256.440(6)	−0.30	43	1.0e+6	D+		5s5d	^3^D_3_	5s9f	^3^F°_3_		B69	TW
2268.29(9)(0)	44072.4	2268.125(13)	0.16	48				5p^2^	^3^P_0_	5s11p	^1^P°_1_		B69	
2269.68(9)(0)	44045.5	2269.562(14)	0.12	100				5p^2^	^3^P_0_	5s11p	^3^P°_1_		B69	
2281.64(16)	43814.6	2281.6301(19)	0.010	330				5s6s	^1^S_0_	5s9p	^1^P°_1_		P38P	
2287.395(10)	43704.37	2287.3947(22)	0.000	130				5s6s	^1^S_0_	5s9p	^3^P°_1_		P38P	
2293.15(9)(1)	43594.7	2293.134(8)	0.02	35				5s5d	^3^D_1_	5s11p	^1^P°_1_		B69	
2294.85(9)(1)	43562.4	2294.603(9)	0.25	25				5s5d	^3^D_1_	5s11p	^3^P°_1_		B69	
2297.72(9)(1)	43508.0	2297.666(8)	0.05	48				5s5d	^3^D_2_	5s11p	^1^P°_1_		B69	
2305.29(9)(1)	43365.1	2305.307(8)	−0.02	360				5p^2^	^3^P_1_	5s9f	^3^F°_2_		B69	
2306.06448(8)	43350.5817	2306.06448(8)	0.00000	19000	2.10e+6	B+		5s^2^	^1^S_0_	5s5p	^3^P°_1_		L93c	CMEF00
2313.217(11)	43216.55	2313.2164(19)	0.001	540	8e+6	E		5s5d	^1^D_2_	5s9p	^1^P°_1_	1	P38P	TW
2314.42(9)(1)	43194.1	2314.422(9)	0.00	65				5s5d	^1^D_2_	5s9p	^3^P°_2_		B69	
2319.154(11)	43105.93	2319.1418(22)	0.012	290	1.5e+6	E		5s5d	^1^D_2_	5s9p	^3^P°_1_	1	P38P	TW
2323.306(16)	43028.9	2323.308(5)	−0.002	83			E2	5s5d	^3^D_1_	5s8f	^3^F°_3_		P38P	
2323.3870(16)	43027.4	2323.405(7)	−0.018	150	5.8e+6	D+		5s5d	^3^D_1_	5s8f	^3^F°_2_		P38P	TW
2326.4910(16)	42970.0	2326.504(6)	−0.013	67				5s5d	^3^D_2_	5s8f	^1^F°_3_		P38P	
2327.949(11)	42943.08	2327.959(5)	−0.010	290	6e+6	D+		5s5d	^3^D_2_	5s8f	^3^F°_3_		P38P	TW
		2328.057(7)			1.1e+6	D+		5s5d	^3^D_2_	5s8f	^3^F°_2_			TW
2334.570(16)	42821.3	2334.584(9)	−0.014	320	7e+6	D+		5s5d	^3^D_3_	5s8f	^3^F°_4_		P38P	TW
		2335.231(5)			8e+5	D+		5s5d	^3^D_3_	5s8f	^3^F°_3_			TW
2343.49(9)(1)	42658.3	2343.298(12)	0.19	310				5s6p	^3^P°_1_	5s18d	^1^D_2_		B69	
2343.87(3)	42651.5	2343.865(16)	0.01	89*			E2	5s6p	^3^P°_1_	5s18d	^3^D_3_		P38P	
2343.87(3)	42651.5	2343.865(23)		89*				5s6p	^3^P°_1_	5s18d	^3^D_2_		P38P	
2343.997(16)	42649.1	2343.997(19)		28				5s6p	^3^P°_1_	5s18d	^3^D_1_	3	P38P	
2350.7432(22)	42526.72	2350.7423(4)	0.0009	2800	1.7e+7	D		5s5p	^1^P°_1_	5p^2^	^3^P_2_		K01c	J07
2355.67(9)(1)	42437.8	2355.862(11)	−0.19	110				5p^2^	^3^P_1_	5s11p	^1^P°_1_		B69	
2356.108(17)	42429.9	2356.107(19)		230				5s6p	^3^P°_1_	5s17d	^1^D_2_	3	P38P	
2356.88(3)	42416.0	2356.88(3)		150*				5s6p	^3^P°_1_	5s17d	^3^D_1_		P38P	
2356.88(3)	42416.0	2356.88(3)		150*				5s6p	^3^P°_1_	5s17d	^3^D_2_		P38P	
2362.863(17)	42308.6	2362.853(14)	0.010	37			E2	5s6p	^3^P°_0_	5s16d	^3^D_2_		P38P	
2363.037(17)	42305.5	2363.009(13)	0.028	34				5s6p	^3^P°_0_	5s16d	^3^D_1_		P38P	
2364.6858699778(4)	42275.995245348	2364.6858699778(4)	0e–10	220	5.1e+0	B+	HF	5s^2^	^1^S_0_	5s5p	^3^P°_0_		Z01	B01
2365.721(17)	42257.5	2365.723(19)		54				5s6p	^3^P°_2_	5s19d	^3^D_3_		P38P	
2367.009(17)	42234.5	2367.015(9)	−0.006	31	1.9e+5	D+		5s6p	^3^P°_0_	5s17s	^3^S_1_		P38P	TW
2370.59(9)(2)	42170.7	2370.459(5)	0.13	580	1.5e+5	E		5s6s	^3^S_1_	5s8p	^1^P°_1_	1	B69	TW
2372.63(9)(2)	42134.5	2372.370(7)	0.26	43	8e+4	E		5p^2^	^3^P_2_	5s10f	^1^F°_3_	1	B69	TW
2372.904(17)	42129.6	2372.909(15)	−0.005	76				5s6p	^3^P°_1_	5s16d	^3^D_2_		P38P	
2373.039(17)	42127.2	2373.066(14)	−0.027	76				5s6p	^3^P°_1_	5s16d	^3^D_1_		P38P	
2373.73(9)(2)	42114.9	2373.716(8)	0.01	220				5p^2^	^3^P_2_	5s10f	^3^F°_3_		B69	
2374.80(9)(2)	42096.0	2374.658(12)	0.14	100				5p^2^	^3^P_0_	5s10p	^3^P°_1_		B69	
2375.960(17)	42075.4	2375.956(13)	0.004	130	2.3e+6	E		5s6s	^3^S_1_	5s8p	^3^P°_2_		P38P	B99
2376.655(17)	42063.1	2376.652(17)	0.003	100				5s6p	^3^P°_2_	5s18d	^3^D_3_		P38P	
2377.085(17)	42055.5	2377.106(9)	−0.021	64	5.4e+5	D+		5s6p	^3^P°_1_	5s17s	^3^S_1_		P38P	TW
2382.630(11)	41957.63	2382.626(5)	0.004	4800	4.6e+7	C+		5s5d	^1^D_2_	5s6f	^1^F°_3_	2	P38G4	B99
2383.76(9)(2)	41937.7	2383.760(3)	0.00	300	2.0e+6	E		5s6s	^3^S_1_	5s8p	^3^P°_1_		B69	B99
2384.526(11)	41924.27	2384.524(4)	0.002	1500				5s5d	^1^D_2_	5s6f	^3^F°_3_		P38G4	
		2386.0(6)			2.3e+6	E		5s6s	^3^S_1_	5s8p	^3^P°_0_			B99
2387.68(9)(2)	41868.9	2387.822(11)	−0.14	500				5p^2^	^3^P_1_	5s8f	^3^F°_2_	2	B69	
		2388.115(10)			2.5e+5	D+		5s6p	^3^P°_0_	5s16s	^3^S_1_			TW
2389.989(11)	41828.45	2389.995(11)	−0.006	*m*				5s6p	^3^P°_2_	5s17d	^3^D_3_		P38	
2393.017(11)	41775.52	2393.019(10)	−0.002	91			E2	5s6p	^3^P°_1_	5s15d	^3^D_3_		P38P	
2393.179(11)	41772.69	2393.180(11)		110				5s6p	^3^P°_1_	5s15d	^3^D_2_		P38P	
		2393.456(13)			7e+5	D+		5s6p	^3^P°_2_	5s18s	^3^S_1_			TW
2397.12(9)(2)	41704.0	2397.225(12)	−0.11	76				5s6p	^3^P°_1_	5s16s	^1^S_0_		B69	
2398.381(17)	41682.1	2398.388(10)	−0.007	22	7e+5	D+		5s6p	^3^P°_1_	5s16s	^3^S_1_		P38P	TW
		2402.086(5)			2.2e+5	D+		5s5d	^3^D_1_	5s10p	^3^P°_1_			TW
		2403.8(10)			1.0e+6	D+		5s5d	^3^D_1_	5s10p	^3^P°_0_			TW
		2405.069(17)			1.5e+5	D+		5s5d	^3^D_2_	5s10p	^3^P°_2_			TW
2405.63(9)(2)	41556.5	2405.551(13)	0.08	120				5s6p	^3^P°_2_	5s16d	^1^D_2_		B69	
2406.473(12)	41541.96	2406.472(12)		220				5s6p	^3^P°_2_	5s16d	^3^D_3_		P38P	
		2407.058(5)			6e+5	D+		5s5d	^3^D_2_	5s10p	^3^P°_1_			TW
2408.566(12)	41505.86	2408.597(14)	−0.031	96			E2	5s6p	^3^P°_0_	5s14d	^3^D_2_		P38P	
2408.758(12)	41502.55	2408.754(10)	0.004	110				5s6p	^3^P°_0_	5s14d	^3^D_1_		P38P	
2410.846(12)	41466.60	2410.836(9)	0.010	93	9e+5	D+		5s6p	^3^P°_2_	5s17s	^3^S_1_		P38P	TW
2412.831(17)	41432.5	2412.831(17)		83	8e+5	D+		5s5d	^3^D_3_	5s10p	^3^P°_2_	3	P38P	TW
2415.409(5)	41388.27	2415.411(4)	−0.002	110	3.2e+5	D+		5s6p	^3^P°_0_	5s15s	^3^S_1_		P38P	TW
2417.21(9)(2)	41357.4	2417.393(13)	−0.18	84				5s6p	^3^P°_1_	5s14d	^1^D_2_		B69	
2418.932(18)	41328.0	2418.923(10)	0.009	69			E2	5s6p	^3^P°_1_	5s14d	^3^D_3_		P38P	
2419.067(18)	41325.7	2419.047(14)	0.020	100				5s6p	^3^P°_1_	5s14d	^3^D_2_		P38P	
2419.196(18)	41323.5	2419.205(10)	−0.009	78				5s6p	^3^P°_1_	5s14d	^3^D_1_		P38P	
2424.42(9)(2)	41234.5	2424.370(18)	0.05	120				5s6p	^3^P°_1_	5s15s	^1^S_0_		B69	
2425.924(18)	41208.9	2425.920(4)	0.004	110	9e+5	D+		5s6p	^3^P°_1_	5s15s	^3^S_1_		P38P	TW
2426.05(9)(2)	41206.8	2425.991(18)	0.06	54				5s6p	^3^P°_2_	5s15d	^1^D_2_		B69	
2427.206(12)	41187.13	2427.205(11)	0.001	98				5s6p	^3^P°_2_	5s15d	^3^D_3_		P38P	
2428.53(9)(2)	41164.7	2428.778(7)	−0.25	370*i*				5s6p	^3^P°_2_	5s_1/2,_*_F_*_=4_13g			B69	
2432.731(12)	41093.59	2432.729(10)	0.002	50	1.2e+6	D+		5s6p	^3^P°_2_	5s16s	^3^S_1_		P38P	TW
		2433.946(8)			7e+5	D+		5p^2^	^3^P_2_	5s9f	^1^F°_3_			TW
2442.471(12)	40929.74	2442.480(11)	−0.009	67			E2	5s6p	^3^P°_0_	5s13d	^3^D_2_		P38P	
2442.634(12)	40927.01	2442.634(12)		110				5s6p	^3^P°_0_	5s13d	^3^D_1_		P38P	
2447.886(12)	40839.21	2447.893(3)	−0.007	220	1.0e+7	D+		5s5d	^3^D_1_	5s7f	^3^F°_2_		P38P	TW
2451.121(12)	40785.30	2451.135(5)	−0.014	2000				5s5d	^3^D_2_	5s7f	^1^F°_3_		P38G4	
2451.549(5)	40778.19	2451.550(4)	−0.001	36	4.4e+5	D+		5s6p	^3^P°_0_	5s14s	^3^S_1_		P38P	TW
2452.910(12)	40755.57	2452.912(3)	−0.002	1400	1.1e+7	D+		5s5d	^3^D_2_	5s7f	^3^F°_3_		P38G4	TW
		2453.0571(23)			1.9e+6	D+		5s5d	^3^D_2_	5s7f	^3^F°_2_			TW
2453.229(12)	40750.26	2453.227(11)	0.002	89				5s6p	^3^P°_1_	5s13d	^3^D_2_		P38P	
2453.856(12)	40739.85	2453.858(11)	−0.002	92				5s6p	^3^P°_2_	5s14d	^3^D_3_		P38P	
2460.188(18)	40635.0	2460.198(9)	−0.010	2500	1.2e+7	D+		5s5d	^3^D_3_	5s7f	^3^F°_4_		P38G4	TW
		2460.986(3)			1.3e+6	D+		5s5d	^3^D_3_	5s7f	^3^F°_3_			TW
2461.070(12)	40620.44	2461.060(4)	0.010	33	1.5e+6	D+		5s6p	^3^P°_2_	5s15s	^3^S_1_		P38P	TW
2462.378(12)	40598.87	2462.377(4)	0.001	17	1.2e+6	D+		5s6p	^3^P°_1_	5s14s	^3^S_1_		P38G4	TW
2486.152(12)	40210.66	2486.141(9)	0.011	160	5e+5	D		5s5p	^1^P°_1_	5p^2^	^3^P_1_		P38P	J07
2488.618(5)	40170.82	2488.619(5)	−0.001	120				5s6p	^3^P°_0_	5s12d	^3^D_1_		P38P	
2488.953(12)	40165.42	2488.953(12)		110				5s6p	^3^P°_2_	5s13d	^3^D_3_		P38P	
2496.318(12)	40046.92	2496.315(9)	0.003	81				5s6p	^3^P°_1_	5s12d	^1^D_2_		P38P	
2498.591(12)	40010.49	2498.588(4)	0.003	93	2.1e+6	D+		5s6p	^3^P°_2_	5s14s	^3^S_1_		P38P	TW
2499.353(12)	39998.29	2499.354(12)	−0.001	32			E2	5s6p	^3^P°_1_	5s12d	^3^D_3_		P38P	
2499.600(12)	39994.34	2499.603(10)	−0.003	110				5s6p	^3^P°_1_	5s12d	^3^D_2_		P38P	
2501.002(5)	39971.93	2501.003(4)	−0.001	67	6e+5	D+		5s6p	^3^P°_0_	5s13s	^3^S_1_		P38P	TW
2508.157(19)	39857.9	2508.159(19)		53				5s6p	^1^P°_1_	5s15d	^1^D_2_		P38P	
2509.320(5)	39839.44	2509.320(4)	0.000	13				5s6p	^3^P°_1_	5s13s	^1^S_0_		P38P	
2512.274(9)	39792.60	2512.272(4)	0.002	96	1.7e+6	D+		5s6p	^3^P°_1_	5s13s	^3^S_1_		P38P	TW
2514.082(13)	39763.98	2514.082(13)		28	8e+5	E		5s6p	^1^P°_1_	5s16s	^1^S_0_	1	P38P	TW
2536.6710(19)	39409.9	2536.669(12)	0.002	400				5s6p	^3^P°_2_	5s12d	^3^D_3_		P38G2	
2543.953(19)	39297.1	2543.955(19)		100	9e+5	E		5s6p	^1^P°_1_	5s15s	^1^S_0_	1	P38G2	TW
2545.747(13)	39269.41	2545.7185(10)	0.028	490			E2	5s5p	^1^P°_1_	5s5d	^3^D_3_		P38G2	
2549.978(13)	39204.26	2549.977(4)	0.001	300	2.9e+6	D+		5s6p	^3^P°_2_	5s13s	^3^S_1_		P38G2	TW
2553.345(8)	39152.57	2553.347(6)	−0.002	15			E2	5s6p	^3^P°_0_	5s11d	^3^D_2_		P38P	
2553.525(20)	39149.8	2553.526(5)	−0.001	51				5s6p	^3^P°_0_	5s11d	^3^D_1_		P38P	
2554.436(13)	39135.85	2554.4164(16)	0.020	1100	1.5e+6	D+		5s5p	^1^P°_1_	5s5d	^3^D_2_		P38G2	J07
		2558.956(6)		*m*	3.6e+6	E		5s6s	^1^S_0_	5s8p	^1^P°_1_		P38	B99
		2560.040(3)		*m*	1.2e+6	D+		5s5p	^1^P°_1_	5s5d	^3^D_1_		L31	J07
2564.738(13)	38978.66	2564.741(9)	−0.003	19			E2	5s6p	^3^P°_1_	5s11d	^3^D_3_		P38P	
2565.098(13)	38973.19	2565.094(6)	0.004	47				5s6p	^3^P°_1_	5s11d	^3^D_2_		P38P	
2565.265(13)	38970.65	2565.274(5)	−0.009	14				5s6p	^3^P°_1_	5s11d	^3^D_1_		P38P	
2571.603(8)	38874.60	2571.6131(23)	−0.010	150	9e+5	D+		5s6p	^3^P°_0_	5s12s	^3^S_1_		P38G2	TW
2573.200(13)	38850.48	2573.191(9)	0.009	95				5s6p	^1^P°_1_	5s13d	^1^D_2_		P38G2	
2574.464(5)	38831.40	2574.464(3)	0.000	28	4.0e+5	E		5s6s	^1^S_0_	5s8p	^3^P°_1_		P38P	B99
2583.523(13)	38695.26	2583.5288(23)	−0.006	220	2.7e+6	D+		5s6p	^3^P°_1_	5s12s	^3^S_1_		P38G2	TW
		2586.696(12)			1.3e+5	D+		5s5d	^3^D_2_	5s9p	^3^P°_2_			TW
		2586.825(4)			1.9e+5	D+		5s5d	^3^D_1_	5s9p	^3^P°_1_			TW
		2588.1(11)			9e+5	D+		5s5d	^3^D_1_	5s9p	^3^P°_0_			TW
2591.946(13)	38569.51	2591.946(13)		220	1.4e+6	D+		5s5p	^1^P°_1_	5p^2^	^3^P_0_		P38G2	J07
		2595.677(12)			7e+5	D+		5s5d	^3^D_3_	5s9p	^3^P°_2_			TW
2598.754(14)	38468.48	2598.753(6)	0.001	700	1.8e+7	D		5s5d	^1^D_2_	5s8p	^1^P°_1_		P38G2	B99
2604.046(14)	38390.31	2604.049(9)	−0.003	540				5s6p	^3^P°_2_	5s11d	^3^D_3_		P38G2	
2604.413(14)	38384.90	2604.413(6)	0.000	160				5s6p	^3^P°_2_	5s11d	^3^D_2_		P38G2	
2604.596(14)	38382.21	2604.599(5)	−0.003	45				5s6p	^3^P°_2_	5s11d	^3^D_1_		P38G2	
2614.755(14)	38233.09	2614.748(3)	0.007	160	2.2e+6	E		5s5d	^1^D_2_	5s8p	^3^P°_1_		P38G2	B99
2623.282(14)	38108.82	2623.287(10)	−0.005	430				5s6p	^1^P°_1_	5s12d	^1^D_2_		P38G2	
2623.435(14)	38106.60	2623.4193(24)	0.016	440	4.5e+6	D+		5s6p	^3^P°_2_	5s12s	^3^S_1_		P38G2	TW
2637.647(14)	37901.29	2637.652(5)	−0.005	9	8e+5	E		5s6p	^1^P°_1_	5s13s	^1^S_0_	1	P38G2,P	TW
2640.920(14)	37854.32	2640.915(4)	0.005	4	7e+4	D+		5s6p	^1^P°_1_	5s13s	^3^S_1_		P38P	TW
2649.960(14)	37725.19	2649.974(4)	−0.014	42			E2	5s6p	^3^P°_0_	5s10d	^3^D_2_		P38G2	
2650.296(11)	37720.41	2650.304(3)	−0.008	500	8e+6	D+		5s6p	^3^P°_0_	5s10d	^3^D_1_		P38G2	TW
2654.708(14)	37657.72	2654.719(5)	−0.011	36				5s6p	^3^P°_1_	5s10d	^1^D_2_		P38P	
2662.636(14)	37545.60	2662.628(4)	0.008	1100	1.0e+7	D+		5s6p	^3^P°_1_	5s10d	^3^D_2_		P38G2	TW
		2662.962(3)			5.4e+6	D+		5s6p	^3^P°_1_	5s10d	^3^D_1_			TW
2668.421(14)	37464.20	2668.434(5)	−0.013	150			E2	5s5d	^3^D_1_	5s6f	^3^F°_3_		P38G2	
2668.658(14)	37460.88	2668.671(6)	−0.013	690	1.8e+7	D		5s5d	^3^D_1_	5s6f	^3^F°_2_		P38G2	B99
2672.175(14)	37411.58	2672.184(6)	−0.009	360				5s5d	^3^D_2_	5s6f	^1^F°_3_		P38G2	
2673.508(14)	37392.93	2673.526(10)	−0.018	85			E2	5s5d	^3^D_2_	5s6f	^3^F°_4_		P38G2	
2674.565(14)	37378.15	2674.571(5)	−0.006	770	1.9e+7	C+		5s5d	^3^D_2_	5s6f	^3^F°_3_		P38G2	B99
2674.797(14)	37374.91	2674.810(6)	−0.013	85	3.4e+6	D+		5s5d	^3^D_2_	5s6f	^3^F°_2_		P38G2	B99
2678.201(5)	37327.40	2678.2098(20)	−0.009	190	2.2e+6	D+		5s6p	^3^P°_0_	5s11s	^3^S_1_		P38G2	TW
2681.744(22)	37278.1	2681.769(6)	−0.025	4				5s5d	^3^D_3_	5s6f	^1^F°_3_		P38P	
2683.118(14)	37259.01	2683.120(10)	−0.002	3400	2.2e+7	C+		5s5d	^3^D_3_	5s6f	^3^F°_4_	2	P38G2	B99
2684.158(14)	37244.57	2684.174(5)	−0.016	200	2.4e+6	D+		5s5d	^3^D_3_	5s6f	^3^F°_3_		P38G2	B99
		2684.414(6)			1.1e+5	D+		5s5d	^3^D_3_	5s6f	^3^F°_2_			TW
2691.132(14)	37148.05	2691.1362(20)	−0.004	580	6e+6	D+		5s6p	^3^P°_1_	5s11s	^3^S_1_		P38G2	TW
2693.883(15)	37110.12	2693.898(7)	−0.015	570				5s6p	^1^P°_1_	5s11d	^1^D_2_		P38G2	
2699.336(15)	37035.16	2699.346(6)	−0.010	4				5s6p	^1^P°_1_	5s11d	^3^D_2_		P38P	
2704.484(15)	36964.67	2704.489(3)	−0.005	570	1.3e+7	D+		5s6p	^3^P°_2_	5s10d	^3^D_3_		P38G2	TW
2705.011(15)	36957.47	2705.019(4)	−0.008	140	3.3e+6	D+		5s6p	^3^P°_2_	5s10d	^3^D_2_		P38G2	TW
		2705.363(3)			3.6e+5	D+		5s6p	^3^P°_2_	5s10d	^3^D_1_			TW
2714.900(15)	36822.85	2714.908(4)	−0.008	130				5s6p	^1^P°_1_	5s12s	^1^S_0_		P38G2	
2734.450(15)	36559.60	2734.4464(20)	0.004	130	1.1e+7	D+		5s6p	^3^P°_2_	5s11s	^3^S_1_		P38G2	TW
2749.746(15)	36356.24	2749.733(4)	0.013	6300	5.9e+7	B		5s5d	^1^D_2_	5s5f	^1^F°_3_		P38G3	J07
2752.793(15)	36316.01	2752.772(4)	0.021	340	3.1e+6	D		5s5d	^1^D_2_	5s5f	^3^F°_3_		P38G6-2	J07
2798.780(16)	35719.32	2798.779(5)	0.001	500				5s6p	^1^P°_1_	5s10d	^1^D_2_		P38G2	
2804.813(24)	35642.5	2804.813(3)	0.000	61			E2	5s6p	^3^P°_0_	5s9d	^3^D_2_		P38P	
2805.337(3)	35635.84	2805.3432(19)	−0.006	500	7e+6	D+		5s6p	^3^P°_0_	5s9d	^3^D_1_		P38G3,2	TW
		2807.572(4)			3.9e+5	D+		5s6p	^1^P°_1_	5s10d	^3^D_2_			TW
		2807.943(3)			2.3e+5	D+		5s6p	^1^P°_1_	5s10d	^3^D_1_			TW
2818.991(16)	35463.24	2818.993(3)	−0.002	860	9e+6	D+		5s6p	^3^P°_1_	5s9d	^3^D_2_		P38G2	TW
2819.527(5)	35456.50	2819.5291(19)	−0.002	350	4.9e+6	D+		5s6p	^3^P°_1_	5s9d	^3^D_1_	2	P38G2	TW
2831.539(16)	35306.10	2831.544(3)	−0.005	330	5e+6	E		5s6p	^1^P°_1_	5s11s	^1^S_0_	1	P38G3,2	TW
2839.53(13)(5)	35206.7	2839.2860(22)	0.24	190	2.7e+5	D+		5s6p	^1^P°_1_	5s11s	^3^S_1_		B69	TW
2852.579(3)	35045.70	2852.5857(17)	−0.007	190	1.6e+6	D+		5s6p	^3^P°_0_	5s10s	^3^S_1_		P38G3,2	TW
2865.682(16)	34885.46	2865.684(12)	−0.002	450	1.2e+7	D+		5s6p	^3^P°_2_	5s9d	^3^D_3_	2	P38G3,2	TW
2866.543(16)	34874.99	2866.552(3)	−0.009	380	2.9e+6	D+		5s6p	^3^P°_2_	5s9d	^3^D_2_		P38G3,2	TW
		2867.1065(20)			3.3e+5	D+		5s6p	^3^P°_2_	5s9d	^3^D_1_			TW
2867.251(3)	34866.38	2867.2546(17)	−0.004	740	4.6e+6	D+		5s6p	^3^P°_1_	5s10s	^3^S_1_		P38G3,2	TW
2890.1708(3)	34589.889	2890.17075(24)	0.0000	10000	2.0e+7	D+		5s5p	^1^P°_1_	5s5d	^1^D_2_		K01c	A86c
2916.466(17)	34278.04	2916.4703(18)	−0.004	1100	8e+6	D+		5s6p	^3^P°_2_	5s10s	^3^S_1_		P38G3	TW
		2938.514(7)			4.7e+5	E		5p^2^	^3^P_2_	5s6f	^1^F°_3_			B99
2941.0373(10)	33991.669	2941.0376(8)	−0.0003	9600	3.4e+8	B		5s5p	^1^P°_1_	5s6s	^1^S_0_		K01c	A86c
		2955.558(20)			1.5e+5	D+		5s5d	^3^D_2_	5s8p	^3^P°_2_			TW
		2956.016(12)			1.7e+7	D+		5p^2^	^1^D_2_	5s10f	^1^F°_3_			TW
		2960.089(5)			2.1e+5	D+		5s5d	^3^D_1_	5s8p	^3^P°_1_			TW
		2963.5(10)			1.0e+6	D+		5s5d	^3^D_1_	5s8p	^3^P°_0_			B99
2966.175(18)	33703.61	2966.170(6)	0.005	4300	1.9e+7	D+		5s6p	^1^P°_1_	5s9d	^1^D_2_		P38G4	TW
		2967.288(20)			8e+5	D+		5s5d	^3^D_3_	5s8p	^3^P°_2_			B99
		2967.644(5)			6e+5	D+		5s5d	^3^D_2_	5s8p	^3^P°_1_			B99
		2970.9507(7)			2.7e+5	D		5s6s	^3^S_1_	5s7p	^1^P°_1_			J07
		2981.979(3)			2.4e+5	D+		5s6p	^1^P°_1_	5s9d	^3^D_2_			TW
2999.4092(4)	33330.182	2999.4090(4)	0.0002	1400	2.1e+6	D		5s6s	^3^S_1_	5s7p	^3^P°_2_		K01c	J07
3022.403(5)	33076.62	3022.402(3)	0.001	170	1.2e+7	E		5s6p	^1^P°_1_	5s10s	^1^S_0_	1	P38G2	TW
3022.904(18)	33071.14	3022.9163(9)	−0.012	84	8e+5	D		5s6s	^3^S_1_	5s7p	^3^P°_1_		P38G2	J07
3028.595(6)	33009.00	3028.5958(19)	−0.001	67	3.1e+6	D+		5s6s	^3^S_1_	5s7p	^3^P°_0_		P38G2	B99
3052.228(19)	32753.43	3052.226(12)	0.002	240	7.2e+7	C		5p^2^	^1^D_2_	5s9f	^1^F°_3_	2	P38G3,2	TW
3069.757(19)	32566.40	3069.760(4)	−0.003	170	1.2e+6	D+		5s6p	^3^P°_1_	5s8d	^1^D_2_		P38P	TW
3083.663(4)	32419.55	3083.6656(24)	−0.003	1000	1.2e+7	D+		5s6p	^3^P°_0_	5s8d	^3^D_1_		P38G3	TW
3099.809(19)	32250.69	3099.813(3)	−0.004	3800	1.6e+7	D+		5s6p	^3^P°_1_	5s8d	^3^D_2_	2	P38G4	TW
3100.814(6)	32240.24	3100.8142(24)	0.000	700	9e+6	D+		5s6p	^3^P°_1_	5s8d	^3^D_1_		P38G2	TW
3138.212(20)	31856.05	3138.217(6)	−0.005	51			E2	5s5d	^3^D_1_	5s5f	^3^F°_3_		P38G4,2	
3138.610(5)	31852.01	3138.609(4)	0.001	1300	3.5e+7	C		5s5d	^3^D_1_	5s5f	^3^F°_2_		K01c	J07
3142.733(15)	31810.22	3142.739(5)	−0.006	560	2.0e+6	D		5s5d	^3^D_2_	5s5f	^1^F°_3_		P38G3,2	J07
3143.862(20)	31798.80	3143.862(14)	0.000	69				5p^2^	^1^D_2_	5s11p	^1^P°_1_		P38G2	
3145.137(20)	31785.91	3145.110(4)	0.027	66			E2	5s5d	^3^D_2_	5s5f	^3^F°_4_		P38G2	
3146.712(20)	31770.00	3146.709(6)	0.003	1600	3.5e+7	B		5s5d	^3^D_2_	5s5f	^3^F°_3_		P38G3,2	J07
3147.099(10)	31766.09	3147.103(4)	−0.004	210	6e+6	D		5s5d	^3^D_2_	5s5f	^3^F°_2_		P38G2	J07
3155.769(20)	31678.83	3155.780(5)	−0.011	650	2.0e+7	D+		5s6p	^3^P°_2_	5s8d	^3^D_3_		P38G2	TW
		3156.005(5)			2.6e+5	D		5s5d	^3^D_3_	5s5f	^1^F°_3_			J07
3157.411(20)	31662.35	3157.416(3)	−0.005	120	5.1e+6	D+		5s6p	^3^P°_2_	5s8d	^3^D_2_		P38G2	TW
3158.396(3)	31652.48	3158.396(3)	0.000	1800	4.2e+7	B		5s5d	^3^D_3_	5s5f	^3^F°_4_		K01c	J07
		3158.454(3)			5.7e+5	D+		5s6p	^3^P°_2_	5s8d	^3^D_1_			TW
3159.997(20)	31636.44	3160.009(5)	−0.012	100	4.2e+6	D		5s5d	^3^D_3_	5s5f	^3^F°_3_		P38G2	J07
		3160.406(4)			1.7e+5	D		5s5d	^3^D_3_	5s5f	^3^F°_2_			J07
3176.272(4)	31474.34	3176.2717(21)	0.000	1400	2.6e+6	D+		5s6p	^3^P°_0_	5s9s	^3^S_1_		P38G3	TW
3194.470(4)	31295.05	3194.4688(21)	0.001	1400	7e+6	D+		5s6p	^3^P°_1_	5s9s	^3^S_1_		P38G3,2	TW
3198.101(20)	31259.52	3198.100(11)	0.001	1300				5p^2^	^1^D_2_	5s8f	^1^F°_3_		P38G3	
3200.851(20)	31232.67	3200.850(10)	0.001	96				5p^2^	^1^D_2_	5s8f	^3^F°_3_		P38G2	
3236.8463(22)	30885.355	3236.8482(9)	−0.0019	450	9e+5	D		5s5p	^1^P°_1_	5s6s	^3^S_1_		K01c	J07
		3255.6774(22)		*m*	1.2e+7	D+		5s6p	^3^P°_2_	5s9s	^3^S_1_		P38	TW
3264.033(5)	30628.11	3264.030(4)	0.003	1700	2.6e+7	D+		5s6p	^1^P°_1_	5s8d	^1^D_2_	2	P38G4	TW
3273.13(9)(14)	30543.0	3273.1178(10)	0.01	170	1.6e+6	E		5s6s	^1^S_0_	5s7p	^1^P°_1_		L31	B99
3298.019(5)	30312.50	3298.028(3)	−0.009	130	4.3e+5	D+		5s6p	^1^P°_1_	5s8d	^3^D_2_		P38G2	TW
		3299.161(3)			3.4e+5	D+		5s6p	^1^P°_1_	5s8d	^3^D_1_			TW
3336.3042(23)	29964.669	3336.3032(12)	0.0010	66	2.5e+5	E		5s6s	^1^S_0_	5s7p	^3^P°_1_	1	K01c	TW
3338.5087(3)	29944.883	3338.5087(3)	0.0000	2300	1.2e+7	D		5s5d	^1^D_2_	5s7p	^1^P°_1_	2	K01c	J07
3345.963(9)	29878.17	3345.962(9)	0.001	520	2.2e+7	E		5p^2^	^1^D_2_	5s10p	^1^P°_1_	1	P38G3	TW
3352.308(9)	29821.62	3352.308(9)	0.000	350	3.2e+6	E		5p^2^	^1^D_2_	5s10p	^3^P°_1_	1	P38G3	TW
3376.593(5)	29607.15	3376.601(3)	−0.008	310	2.1e+7	E		5s6p	^1^P°_1_	5s9s	^1^S_0_	1,2	P38G3	TW
3404.2701(12)	29366.446	3404.2691(9)	0.0010	600	6e+5	D		5s5d	^1^D_2_	5s7p	^3^P°_1_		K01c	J07
		3405.3848(25)			2.8e+5	D+		5s6p	^1^P°_1_	5s9s	^3^S_1_			TW
3438.417(24)	29074.82	3438.413(9)	0.004	800	1.6e+6	E		5p^2^	^1^D_2_	5s7f	1F°3	1	P38G4,3	TW
3441.911(24)	29045.30	3441.911(6)	0.000	230				5p^2^	^1^D_2_	5s7f	^3^F°_3_		P38G4,3	
3517.686(25)	28419.65	3517.697(6)	−0.011	210	6e+5	D		5p^2^	^3^P_2_	5s5f	^1^F°_3_		P38G3	J07
3522.671(25)	28379.44	3522.671(7)	0.000	340				5p^2^	^3^P_2_	5s5f	^3^F°_3_		P38G1	
3627.612(11)	27558.49	3627.6323(10)	−0.020	140	1.3e+6	D		5s6p	^3^P°_1_	5s7d	^1^D_2_		P38G3,2	J07
3693.9239(14)	27063.781	3693.9239(11)	0.0000	430	2.5e+7	C+		5s6p	^3^P°_0_	5s7d	^3^D_1_		K01c	J07
3708.133(11)	26960.08	3708.133(5)	0.000	290	1.0e+7	E		5p^2^	^1^D_2_	5s9p	^1^P°_1_	1,2	P38G3	TW
3716.1350(11)	26902.026	3716.1349(10)	0.0001	760	3.3e+7	C+		5s6p	^3^P°_1_	5s7d	^3^D_2_		K01c	J07
3718.5578(21)	26884.499	3718.5582(12)	−0.0004	370	1.8e+7	C+		5s6p	^3^P°_1_	5s7d	^3^D_1_		K01c	J07
3723.389(11)	26849.62	3723.381(6)	0.008	540	1.8e+6	E		5p^2^	^1^D_2_	5s9p	^3^P°_1_	1	P38G3,2	TW
3795.2048(10)	26341.560	3795.2048(10)	0.0000	1100	4.2e+7	C+		5s6p	^3^P°_2_	5s7d	^3^D_3_		K01c	J07
3799.2255(22)	26313.683	3799.2257(11)	−0.0002	300	1.04e+7	C+		5s6p	^3^P°_2_	5s7d	^3^D_2_		K01c	J07
3801.760(6)	26296.14	3801.7586(13)	0.001	390	1.2e+6	D+		5s6p	^3^P°_2_	5s7d	^3^D_1_		P38G3	J07
3802.757(12)	26289.25	3802.758(9)	−0.001	19bl*				5s4f	^3^F°_4_	5s_1/2,_*_F_*_=5_14g			P38TP	
3803.260(12)	26285.77	3803.261(11)	−0.001	19bl*				5s4f	^3^F°_4_	5s_1/2,_*_F_*_=4_14g			P38TP	
3807.731(15)	26254.91	3807.729(9)	0.002	9				5s4f	^1^F°_3_	5s_1/2,_*_F_*_=5_14g			P38TP	
3808.24(3)	26251.40	3808.234(11)	0.01	9				5s4f	^1^F°_3_	5s_1/2,_*_F_*_=4_14g			P38TP	
3834.6306(4)	26070.735	3834.6307(4)	−0.0001	32000	2.20e+8	B		5s5d	^1^D_2_	5s4f	^1^F°_3_	2	K01c	M96
3842.189(15)	26019.45	3842.158(3)	0.031	900	2.1e+7	C		5s5d	^1^D_2_	5s4f	^3^F°_3_	2	P38G4	M96
		3842.918(4)		5600				5s5d	^1^D_2_	5s4f	^3^F°_2_		W96	
3853.04(3)	25946.18	3853.034(17)	0.01	15				5s4f	^3^F°_3_	5s_1/2,_*_F_*_=5_13g			P38TP	
3853.55(3)	25942.71	3853.553(17)	0.00	44				5s4f	^3^F°_3_	5s_1/2,_*_F_*_=4_13g			P38TP	
3855.52(3)	25929.49	3855.524(17)	0.00	69				5s4f	^3^F°_4_	5s_1/2,_*_F_*_=5_13g			P38TP	
3856.04(3)	25926.02	3856.043(17)	0.00	7				5s4f	^3^F°_4_	5s_1/2,_*_F_*_=4_13g			P38TP	
3860.63(3)	25895.16	3860.634(17)	0.00	46				5s4f	^1^F°_3_	5s_1/2,_*_F_*_=5_13g			P38TP	
3861.16(3)	25891.61	3861.154(17)	0.01	21				5s4f	^1^F°_3_	5s_1/2,_*_F_*_=4_13g			P38TP	
		3888.0738(19)			1.8e+6	D+		5s6p	^3^P°_1_	5s8s	^1^S_0_			J07
3889.78(3)	25701.13	3889.764(12)	0.02	470	3.4e+6	D		5p^2^	^1^D_2_	5s6f	^1^F°_3_		P38G2,1	B99
3894.82(3)	25667.86	3894.824(10)	0.00	65				5p^2^	^1^D_2_	5s6f	^3^F°_3_		P38TP	
3902.0795(8)	25620.103	3902.0794(8)	0.0001	910	4.0e+7	B		5s6p	^1^P°_1_	5s7d	^1^D_2_	2	K01c	J07
3921.32(3)	25494.38	3921.318(16)	0.00	19				5s4f	^3^F°_2_	5s_1/2,_*_F_*_=4_12g			P38TP	
3921.57(3)	25492.76	3921.567(18)	0.00	19				5s4f	^3^F°_3_	5s_1/2,_*_F_*_=5_12g			P38TP	
3922.12(3)	25489.21	3922.110(16)	0.01	80				5s4f	^3^F°_3_	5s_1/2,_*_F_*_=4_12g			P38TP	
3924.14(3)	25476.07	3924.146(18)	−0.01	45				5s4f	^3^F°_4_	5s_1/2,_*_F_*_=5_12g			P38TP	
3924.68(3)	25472.57	3924.689(16)	−0.01	11				5s4f	^3^F°_4_	5s_1/2,_*_F_*_=4_12g			P38TP	
3929.44(3)	25441.73	3929.439(18)	0.00	52				5s4f	^1^F°_3_	5s_1/2,_*_F_*_=5_12g			P38TP	
3929.98(3)	25438.24	3929.985(16)	−0.01	36				5s4f	^1^F°_3_	5s_1/2,_*_F_*_=4_12g			P38TP	
3934.3742(19)	25409.809	3934.3750(9)	−0.0008	240	5.6e+6	D+		5s6p	^3^P°_0_	5s8s	^3^S_1_		K01c	J07
		3936.106(4)			1.2e+5	D+		5s5d	^3^D_2_	5s7p	^1^P°_1_			TW
3962.3330(14)	25230.518	3962.3328(8)	0.0002	490	1.6e+7	D+		5s6p	^3^P°_1_	5s8s	^3^S_1_		K01c	J07
		3986.213(4)			1.7e+5	D+		5s5d	^3^D_2_	5s7p	^3^P°_2_			B99
4004.668(13)	24963.80	4004.6685(15)	−0.001	79	6e+5	D+		5s6p	^1^P°_1_	5s7d	^3^D_2_		P38G2,TP	J07
		4007.4829(17)			5.2e+5	D+		5s6p	^1^P°_1_	5s7d	^3^D_1_			J07
4007.5830(24)	24945.644	4007.5788(21)	0.0042	110	2.4e+5	D		5s5d	^3^D_3_	5s7p	^3^P°_2_		K01c	J07
4012.52(3)	24914.97	4012.521(13)	0.00	12				5s4f	^3^F°_2_	5s_1/2,_*_F_*_=5_11g			P38TP	
4013.10(3)	24911.36	4013.090(12)	0.01	28				5s4f	^3^F°_2_	5s_1/2,_*_F_*_=4_11g			P38TP	
4013.35(3)	24909.77	4013.351(13)	0.00	42				5s4f	^3^F°_3_	5s_1/2,_*_F_*_=5_11g			P38TP	
4013.92(3)	24906.26	4013.920(12)	0.00	96				5s4f	^3^F°_3_	5s_1/2,_*_F_*_=4_11g			P38TP	
		4013.935(7)			2.5e+5	D+		5s5d	^3^D_1_	5s7p	^3^P°_1_			B99
4016.04(3)	24893.14	4016.052(13)	−0.01	61				5s4f	^3^F°_4_	5s_1/2,_*_F_*_=5_11g			P38TP	
4016.61(3)	24889.58	4016.622(12)	−0.01	7				5s4f	^3^F°_4_	5s_1/2,_*_F_*_=4_11g			P38TP	
4021.58(3)	24858.84	4021.597(12)	−0.02	67				5s4f	^1^F°_3_	5s_1/2,_*_F_*_=5_11g			P38TP	
4022.17(3)	24855.17	4022.168(12)	0.00	34				5s4f	^1^F°_3_	5s_1/2,_*_F_*_=4_11g			P38TP	
4023.88(5)	24844.6	4023.954(7)	−0.07	38*bl*	1.1e+6	D+		5s5d	^3^D_1_	5s7p	^3^P°_0_		P38TP	B99
4027.79(3)	24820.51	4027.838(4)	−0.05	54	7e+5	D+		5s5d	^3^D_2_	5s7p	^3^P°_1_		P38TP	B99
4056.9378(12)	24642.173	4056.9377(9)	0.0001	1300	2.6e+7	C		5s6p	^3^P°_2_	5s8s	^3^S_1_		K01c	J07
4109.34(3)	24327.96	4109.34(3)		13				5s6d	^3^D_1_	5s12f	^3^F°_2_		P38TP	
4109.87(3)	24324.78	4109.86(4)	0.01	8			E2	5s6d	^3^D_1_	5s12f	^3^F°_3_		P38TP	
4112.01(3)	24312.17	4112.01(3)		5				5s6d	^3^D_2_	5s12f	^1^F°_3_		P38TP	
4115.61(5)	24290.9	4115.61(4)	0.00	10				5s6d	^3^D_2_	5s12f	^3^F°_3_		P38TP	
4122.79(3)	24248.58	4122.79(3)		25				5s6d	^3^D_3_	5s12f	^3^F°_4_		P38TP	
4139.97(3)	24147.93	4139.974(11)	0.00	38				5s4f	^3^F°_2_	5s_1/2,_*_F_*_=5_10g			P38TP	
4140.588(14)	24144.35	4140.579(9)	0.009	110				5s4f	^3^F°_2_	5s_1/2,_*_F_*_=4_10g			P38G1	
4140.854(17)	24142.80	4140.857(11)	−0.003	110				5s4f	^3^F°_3_	5s_1/2,_*_F_*_=5_10g			P38G1	
4141.458(17)	24139.28	4141.462(9)	−0.004	190				5s4f	^3^F°_3_	5s_1/2,_*_F_*_=4_10g			P38G1	
4143.73(3)	24126.03	4143.732(11)	0.00	38				5s4f	^3^F°_4_	5s_1/2,_*_F_*_=5_10g			P38G1	
4144.33(3)	24122.55	4144.339(10)	−0.01	19				5s4f	^3^F°_4_	5s_1/2,_*_F_*_=4_10g			P38G1	
4149.63(3)	24091.72	4149.635(11)	−0.01	550				5s4f	^1^F°_3_	5s_1/2,_*_F_*_=5_10g			P38G4	
4150.24(3)	24088.22	4150.243(9)	0.00	100				5s4f	^1^F°_3_	5s_1/2,_*_F_*_=4_10g			P38G1	
4205.0626(19)	23774.161	4205.0626(19)		290	3.9e+7	C+		5s6p	^1^P°_1_	5s8s	^1^S_0_		K01c	J07
4213.01(5)	23729.3	4213.007(23)	0.00	450				5s6d	^3^D_1_	5s11f	^3^F°_2_		P38G1,TP	
4213.66(4)	23725.65	4213.659(24)	0.00	330			E2	5s6d	^3^D_1_	5s11f	^3^F°_3_		P38G1,TP	
4215.59(4)	23714.78	4215.59(3)	0.00	57				5s6d	^3^D_2_	5s11f	^1^F°_3_		P38G1,TP	
4219.08(5)	23695.2	4219.050(23)	0.03	170				5s6d	^3^D_2_	5s11f	^3^F°_2_		P38G1,TP	
4219.70(5)	23691.7	4219.703(24)	0.00	450				5s6d	^3^D_2_	5s11f	^3^F°_3_		P38G1,TP	
4227.16(4)	23649.89	4227.17(3)	−0.01	240				5s6d	^3^D_3_	5s11f	^3^F°_4_		P38G1,TP	
4228.55(5)	23642.1	4228.590(23)	−0.04	240				5s6d	^3^D_3_	5s11f	^3^F°_2_		P38G1,TP	
4229.23(5)	23638.3	4229.247(24)	−0.02	190				5s6d	^3^D_3_	5s11f	^3^F°_3_		P38G1,TP	
4292.064(11)	23292.26	4292.0586(15)	0.005	100	4.8e+5	D+		5s6p	^1^P°_1_	5s8s	^3^S_1_		P38G3	J07
4325.684(7)	23111.23	4325.688(6)	−0.004	440				5s4f	^3^F°_2_	5s_1/2,_*_F_*_=5_9g			P38G3	
4326.375(15)	23107.54	4326.367(9)	0.008	330				5s4f	^3^F°_2_	5s_1/2,_*_F_*_=4_9g			P38G3	
4326.645(15)	23106.10	4326.652(7)	−0.007	330				5s4f	^3^F°_3_	5s_1/2,_*_F_*_=5_9g			P38G3	
4327.332(15)	23102.43	4327.331(9)	0.001	390				5s4f	^3^F°_3_	5s_1/2,_*_F_*_=4_9g			P38G3	
4329.80(4)	23089.24	4329.791(8)	0.01	290				5s4f	^3^F°_4_	5s_1/2,_*_F_*_=5_9g			P38G3	
4330.46(4)	23085.77	4330.472(9)	−0.01	290				5s4f	^3^F°_4_	5s_1/2,_*_F_*_=4_9g			P38G3	
4336.26(4)	23054.89	4336.237(7)	0.02	200				5s4f	^1^F°_3_	5s_1/2,_*_F_*_=5_9g			P38G3	
4336.93(4)	23051.33	4336.920(9)	0.01	58				5s4f	^1^F°_3_	5s_1/2,_*_F_*_=4_9g			P38G3	
4358.00(4)	22939.85	4358.00(3)	0.00	110	2.2e+6	D+		5s6d	^3^D_1_	5s10f	^3^F°_2_		P38G2	TW
4358.39(4)	22937.78	4358.40(3)	−0.01	48			E2	5s6d	^3^D_1_	5s10f	^3^F°_3_		P38G2	
4360.31(4)	22927.69	4360.32(3)	−0.01	110				5s6d	^3^D_2_	5s10f	^1^F°_3_		P38G2,1	
4364.46(4)	22905.92	4364.46(3)	0.00	170	4.0e+5	D+		5s6d	^3^D_2_	5s10f	^3^F°_2_		P38G2	TW
4364.87(4)	22903.74	4364.87(3)	0.00	230	2.3e+6	D+		5s6d	^3^D_2_	5s10f	^3^F°_3_		P38G2	TW
4372.90(4)	22861.69	4372.89(3)	0.01	360	2.6e+6	D+		5s6d	^3^D_3_	5s10f	^3^F°_4_		P38G3,2,1	TW
4375.08(4)	22850.30	4375.08(3)	0.00	27	2.9e+5	D+		5s6d	^3^D_3_	5s10f	^3^F°_3_		P38G1	TW
		4448.423(16)			2.9e+5	D+		5p^2^	^1^S_0_	5s10p	^1^P°_1_			TW
		4489.11(4)			5.3e+5	D+		5s7p	^3^P°_2_	5s16s	^3^S_1_			TW
4500.804(20)	22212.02	4500.806(18)	−0.002	360	9.7e+6	C+		5p^2^	^1^D_2_	5s8p	^1^P°_1_		P38G3,2	B99
4549.007(21)	21976.66	4548.998(10)	0.009	540	1.1e+6	E		5p^2^	^1^D_2_	5s8p	^3^P°_1_		P38G3,2	B99
4570.877(8)	21871.51	4570.881(8)	−0.004	520	4.0e+6	D+		5s6d	^3^D_1_	5s9f	^3^F°_2_		P38G3	TW
4571.320(8)	21869.39	4571.286(23)	0.034	510			E2	5s6d	^3^D_1_	5s9f	^3^F°_3_		P38G3	
4572.95(4)	21861.61	4572.94(3)	0.01	170				5s6d	^3^D_2_	5s9f	^1^F°_3_		P38G3	
4578.02(4)	21837.40	4577.995(8)	0.03	530bl*	7e+5	D+		5s6d	^3^D_2_	5s9f	^3^F°_2_		P38G3	TW
4578.40(4)	21835.58	4578.401(23)	0.00	530bl*	4.2e+6	D+		5s6d	^3^D_2_	5s9f	^3^F°_3_		P38G3	TW
		4586.527(15)			7e+5	D+		5s7p	^3^P°_2_	5s15s	^3^S_1_			TW
4587.03(4)	21794.51	4587.01(3)	0.02	520	4.7e+6	D+		5s6d	^3^D_3_	5s9f	^3^F°_4_		P38G3	TW
4589.28(6)	21783.8	4589.230(8)	0.05	57*bl*				5s6d	^3^D_3_	5s9f	^3^F°_2_		P38G1	
4589.64(4)	21782.09	4589.639(23)	0.00	54*bl*	5.2e+5	D+		5s6d	^3^D_3_	5s9f	^3^F°_3_		P38G1	TW
4615.310(21)	21660.95	4615.307(9)	0.003	200				5s4f	^3^F°_2_	5s_1/2,_*_F_*_=5_8g			P38G3	
4616.083(17)	21657.32	4616.068(9)	0.015	540				5s4f	^3^F°_2_	5s_1/2,_*_F_*_=4_8g			P38G3	
4616.405(17)	21655.81	4616.405(9)	0.000	140				5s4f	^3^F°_3_	5s_1/2,_*_F_*_=5_8g			P38G3	
4617.175(21)	21652.20	4617.166(9)	0.009	350				5s4f	^3^F°_3_	5s_1/2,_*_F_*_=4_8g			P38G3	
4619.987(21)	21639.02	4619.979(10)	0.008	410				5s4f	^3^F°_4_	5s_1/2,_*_F_*_=5_8g			P38G3	
4620.728(13)	21635.55	4620.741(8)	−0.013	350				5s4f	^3^F°_4_	5s_1/2,_*_F_*_=4_8g			P38G3	
4627.312(17)	21604.77	4627.318(9)	−0.006	320				5s4f	^1^F°_3_	5s_1/2,_*_F_*_=5_8g			P38G3	
4628.074(17)	21601.21	4628.083(8)	−0.009	510				5s4f	^1^F°_3_	5s_1/2,_*_F_*_=4_8g			P38G3	
4637.047(9)	21559.41	4637.055(9)	−0.008	610			E2	5s5d	^3^D_1_	5s4f	^3^F°_3_		P38G3	
4638.168(9)	21554.20	4638.162(7)	0.006	8800	1.77e+8	B		5s5d	^3^D_1_	5s4f	^3^F°_2_		P38G3	J07,M96
4644.581(9)	21524.44	4644.572(5)	0.009	5900	1.4e+7	C		5s5d	^3^D_2_	5s4f	^1^F°_3_	2	P38G3	M96
4652.00(4)	21490.13	4651.990(7)	0.01	240			E2	5s5d	^3^D_2_	5s4f	^3^F°_4_		P38G3	
4655.621(9)	21473.40	4655.620(6)	0.001	7800	1.84e+8	B		5s5d	^3^D_2_	5s4f	^3^F°_3_		P38G3	M96
4656.740(9)	21468.24	4656.736(6)	0.004	1700	3.3e+7	B		5s5d	^3^D_2_	5s4f	^3^F°_2_		P38G3	J07,M96
		4661.571(15)			5.3e+5	D+		5s7p	^3^P°_1_	5s14s	^3^S_1_			TW
4673.599(9)	21390.80	4673.605(3)	−0.006	390	1.7e+6	D+		5s5d	^3^D_3_	5s4f	^1^F°_3_		P38G3	M96
4681.105(9)	21356.50	4681.115(6)	−0.010	16000	2.00e+8	B		5s5d	^3^D_3_	5s4f	^3^F°_4_	2	P38G3	A86,M96
4684.779(9)	21339.75	4684.791(5)	−0.012	2500	1.80e+7	B		5s5d	^3^D_3_	5s4f	^3^F°_3_		P38G3	M96
		4685.921(6)			9e+5	D+		5s5d	^3^D_3_	5s4f	^3^F°_2_			J07,M96
		4718.597(16)			9e+5	D+		5s7p	^3^P°_2_	5s14s	^3^S_1_			TW
4747.54(5)	21057.64	4747.57(3)	−0.03	83				5s7p	^1^P°_1_	5s13d	^1^D_2_		P38G1	
4783.24(7)	20900.5	4783.204(18)	0.04	27				5s7p	^3^P°_0_	5s12d	^3^D_1_		P38G1	
4796.82(7)	20841.3	4796.80(4)	0.02	79				5s7p	^3^P°_1_	5s12d	^3^D_2_		P38G1	
		4843.674(14)			7e+5	D+		5s7p	^3^P°_1_	5s13s	^3^S_1_			TW
4856.27(7)	20586.2	4856.27(4)	0.00	88				5s7p	^3^P°_2_	5s12d	^3^D_3_		P38G1	
4902.18(5)	20393.38	4902.14(3)	0.04	56	6e+5	D		5p^2^	^3^P_1_	5s4f	^3^F°_2_		P38G1	J07
4905.29(5)	20380.47	4905.271(15)	0.02	14	1.3e+6	D+		5s7p	^3^P°_2_	5s13s	^3^S_1_		P38G1	TW
4906.64(5)	20374.85	4906.639(23)	0.00	42			E2	5s6d	^3^D_1_	5s8f	^3^F°_3_		P38G1	
4907.07(5)	20373.08	4907.07(3)	0.00	180	4.2e+6	D+		5s6d	^3^D_1_	5s8f	^3^F°_2_		P38G1	TW
4908.36(5)	20367.70	4908.36(3)	0.00	85				5s6d	^3^D_2_	5s8f	^1^F°_3_		P38G1	
4914.85(5)	20340.83	4914.837(23)	0.01	83	4.5e+6	D+		5s6d	^3^D_2_	5s8f	^3^F°_3_		P38G1	TW
4915.30(5)	20338.96	4915.27(3)	0.03	14	8e+5	D+		5s6d	^3^D_2_	5s8f	^3^F°_2_		P38G1	TW
4924.93(5)	20299.17	4924.91(4)	0.02	140	5.0e+6	C		5s6d	^3^D_3_	5s8f	^3^F°_4_		P38G1	TW
4927.82(5)	20287.30	4927.789(23)	0.03	57	5.5e+5	D+		5s6d	^3^D_3_	5s8f	^3^F°_3_		P38G1	TW
4971.75(5)	20108.05	4971.730(17)	0.02	47	1.0e+6	E		5s7p	^1^P°_1_	5s13s	^1^S_0_	1	P38G1	TW
4973.776(20)	20099.84	4973.754(12)	0.022	460	2.8e+6	D		5p^2^	^1^D_2_	5s5f	^1^F°_3_		P38G3	J07
4983.76(5)	20059.56	4983.704(13)	0.06	74	1.9e+5	D		5p^2^	^1^D_2_	5s5f	^3^F°_3_		P38G3	J07
		5006.76(3)			3.8e+6	D+		5s6d	^1^D_2_	5s9f	^1^F°_3_			TW
		5028.874(21)		*m*				5s7p	^3^P°_0_	5s11d	^3^D_1_		P38	
5043.93(5)	19820.27	5043.913(22)	0.02	170*bl*				5s7p	^3^P°_1_	5s11d	^3^D_2_		P38G1	
5044.623(25)	19817.56	5044.611(20)	0.012	100*bl*				5s7p	^3^P°_1_	5s11d	^3^D_1_		P38G1	
5099.500(13)	19604.30	5099.504(9)	−0.004	33	4.1e+5	D+		5s7p	^3^P°_0_	5s12s	^3^S_1_		P38G1	TW
5109.36(5)	19566.47	5109.34(4)	0.02	140				5s7p	^3^P°_2_	5s11d	^3^D_3_		P38G1	
5110.75(5)	19561.14	5110.744(23)	0.01	30				5s7p	^3^P°_2_	5s11d	^3^D_2_		P38G1	
5112.234(13)	19555.47	5112.234(10)	0.000	31	4.6e+5	D+		5p^2^	^1^S_0_	5s9p	^1^P°_1_		P38G1	TW
5115.10(3)	19544.51	5115.108(14)	−0.01	800				5s4f	^3^F°_2_	5s_1/2,_*_F_*_=5_7g			P38G3,1	
		5115.687(9)			1.1e+6	D+		5s7p	^3^P°_1_	5s12s	^3^S_1_			TW
5116.06(3)	19540.85	5116.040(14)	0.02	590				5s4f	^3^F°_2_	5s_1/2,_*_F_*_=4_7g			P38G3	
5116.47(3)	19539.28	5116.456(14)	0.01	350				5s4f	^3^F°_3_	5s_1/2,_*_F_*_=5_7g			P38G3	
5117.392(21)	19535.76	5117.388(13)	0.004	810				5s4f	^3^F°_3_	5s_1/2,_*_F_*_=4_7g			P38G3	
5120.839(21)	19522.61	5120.847(14)	−0.008	960				5s4f	^3^F°_4_	5s_1/2,_*_F_*_=5_7g			P38G3	
5121.768(21)	19519.07	5121.781(13)	−0.013	880				5s4f	^3^F°_4_	5s_1/2,_*_F_*_=4_7g			P38G3,1	
5129.86(5)	19488.28	5129.865(14)	−0.01	710				5s4f	^1^F°_3_	5s_1/2,_*_F_*_=5_7g			P38G3	
5130.78(5)	19484.78	5130.802(13)	−0.02	240				5s4f	^1^F°_3_	5s_1/2,_*_F_*_=4_7g			P38G3,1	
		5158.54(5)			3.0e+5	D+		5s7s	^3^S_1_	5s9p	^3^P°_2_			TW
5175.41(3)	19316.77	5175.394(24)	0.02	200				5s7p	^1^P°_1_	5s11d	^1^D_2_		P38G1	
5184.455(13)	19283.06	5184.446(9)	0.009	160	2.0e+6	D+		5s7p	^3^P°_2_	5s12s	^3^S_1_		P38G1	TW
5253.498(14)	19029.64	5253.495(13)	0.003	48	9e+5	E		5s7p	^1^P°_1_	5s12s	^1^S_0_	1	P38G1	TW
5258.16(6)	19012.78	5258.16(4)	0.00	26				5s6d	^1^D_2_	5s11p	^1^P°_1_		P38G1	
5309.4926(17)	18828.953	5309.4926(16)	0.0000	550	2.6e+6	D		5s6p	^3^P°_1_	5s6d	^1^D_2_		K01c	J07
5402.54(3)	18504.68	5402.539(19)	0.00	110				5s7p	^3^P°_1_	5s10d	^1^D_2_		P38G1	
5411.65(6)	18473.51	5411.65(3)	0.00	130	2.4e+6	E		5s6d	^1^D_2_	5s8f	^1^F°_3_	1	P38G1	TW
5417.09(6)	18454.97	5417.131(17)	−0.04	18			E2	5s7p	^3^P°_0_	5s10d	^3^D_2_		P38G1	
5418.513(15)	18450.12	5418.514(11)	−0.001	140	3.2e+6	D+		5s7p	^3^P°_0_	5s10d	^3^D_1_		P38G1	TW
5433.29(6)	18399.95	5433.258(12)	0.03	18			E2	5s7p	^3^P°_1_	5s10d	^3^D_3_		P38G1	
5435.38(3)	18392.88	5435.396(16)	−0.02	440	3.9e+6	D+		5s7p	^3^P°_1_	5s10d	^3^D_2_		P38G3	TW
5436.792(15)	18388.09	5436.789(11)	0.003	160	2.2e+6	D+		5s7p	^3^P°_1_	5s10d	^3^D_1_		P38G1	TW
5496.74(3)	18187.55	5496.757(14)	−0.02	110			E2	5s6d	^3^D_1_	5s7f	^3^F°_3_		P38G1	
5497.492(12)	18185.06	5497.486(8)	0.006	1000	7.5e+6	C		5s6d	^3^D_1_	5s7f	^3^F°_2_		P38G3	TW
5498.11(3)	18183.01	5498.098(22)	0.01	390				5s6d	^3^D_2_	5s7f	^1^F°_3_		P38G3	
5503.11(6)	18166.49	5503.10(5)	0.01	64			E2	5s6d	^3^D_2_	5s7f	^3^F°_4_		P38G1	
5507.053(18)	18153.49	5507.048(14)	0.005	1000	7.8e+6	C		5s6d	^3^D_2_	5s7f	^3^F°_3_		P38G3	TW
5507.772(12)	18151.12	5507.779(9)	−0.007	570	1.4e+6	D+		5s6d	^3^D_2_	5s7f	^3^F°_2_		P38G3	TW
5510.884(12)	18140.87	5510.883(12)	0.001	1000	5.5e+6	C		5s7p	^3^P°_2_	5s10d	^3^D_3_		P38G3	TW
5513.0057(21)	18133.888	5513.0060(21)	−0.0003	1500	4.9e+6	C		5p^2^	^3^P_2_	5s4f	^1^F°_3_		K01c	J07
5513.10(3)	18133.58	5513.083(16)	0.02	350	1.4e+6	D+		5s7p	^3^P°_2_	5s10d	^3^D_2_		P38G3	TW
5514.53(6)	18128.88	5514.516(11)	0.01	23	1.5e+5	D+		5s7p	^3^P°_2_	5s10d	^3^D_1_		P38G1	TW
5519.36(6)	18113.01	5519.35(5)	0.01	1100	8.8e+6	C		5s6d	^3^D_3_	5s7f	^3^F°_4_		P38G3	TW
5523.31(3)	18100.07	5523.314(15)	0.00	180	1.0e+6	D+		5s6d	^3^D_3_	5s7f	^3^F°_3_		P38G1	TW
5525.965(15)	18091.36	5525.965(11)	0.000	43				5s7p	^3^P°_1_	5s11s	^1^S_0_		P38G1	
5528.600(21)	18082.74	5528.577(7)	0.023	290	7e+5	D		5p^2^	^3^P_2_	5s4f	^3^F°_3_		K01c	J07
5530.16(5)	18077.65	5530.151(9)	0.01	33				5p^2^	^3^P_2_	5s4f	^3^F°_2_		P38G1	
5536.438(15)	18057.14	5536.445(9)	−0.007	130	8e+5	D+		5s7p	^3^P°_0_	5s11s	^3^S_1_		P38G1	TW
5555.529(15)	17995.09	5555.525(9)	0.004	140	2.3e+6	D+		5s7p	^3^P°_1_	5s11s	^3^S_1_		P38G1	TW
5576.88(3)	17926.19	5576.866(20)	0.01	1200				5s7p	^1^P°_1_	5s10d	^1^D_2_		P38G3	
5611.89(3)	17814.36	5611.884(17)	0.01	28	3.2e+5	D+		5s7p	^1^P°_1_	5s10d	^3^D_2_		P38G1	TW
5636.733(16)	17735.85	5636.710(9)	0.023	140	4.0e+6	D+		5s7p	^3^P°_2_	5s11s	^3^S_1_		P38G1	TW
5708.486(16)	17512.92	5708.483(11)	0.003	270				5s7p	^1^P°_1_	5s11s	^1^S_0_		P38G1	
5721.807(16)	17472.15	5721.806(12)	0.001	130				5s7s	^1^S_0_	5s9p	^1^P°_1_		P38G1	
5758.184(17)	17361.77	5758.194(13)	−0.010	32				5s7s	^1^S_0_	5s9p	^3^P°_1_		P38G1	
5853.1713(10)	17080.021	5853.1709(9)	0.0004	3400	6.3e+7	B		5s6p	^3^P°_0_	5s6d	^3^D_1_		K01c	J07
5903.3920(10)	16934.721	5903.3916(9)	0.0004	9000	8.1e+7	B		5s6p	^3^P°_1_	5s6d	^3^D_2_		K01c	J07
5915.2623(14)	16900.738	5915.2627(12)	−0.0004	1300	4.5e+7	B		5s6p	^3^P°_1_	5s6d	^3^D_1_		K01c	J07
5918.7693(11)	16890.724	5918.7693(10)	0.0000	5100	8.4e+7	B		5s6p	^1^P°_1_	5s6d	^1^D_2_		K01c	J07
6062.96(4)	16489.02	6062.970(25)	−0.01	130	8e+5	D+		5s7p	^3^P°_1_	5s9d	^1^D_2_		P38G1	TW
6095.9333(19)	16399.839	6095.9334(19)	−0.0001	3300	1.09e+8	B		5s6p	^3^P°_2_	5s6d	^3^D_3_		K01c	J07
6108.706(19)	16365.55	6108.689(11)	0.017	140	3.7e+6	D+		5s7p	^3^P°_0_	5s9d	^3^D_1_		P38G1	TW
6115.8707(15)	16346.377	6115.8707(12)	0.0000	830	2.7e+7	B		5s6p	^3^P°_2_	5s6d	^3^D_2_		K01c	J07
6128.62(4)	16312.38	6128.6126(17)	0.01	130*bl*	3.0e+6	D+		5s6p	^3^P°_2_	5s6d	^3^D_1_		P38G1	J07
6129.391(19)	16310.32	6129.391(12)	0.000	160	4.6e+6	C		5s7p	^3^P°_1_	5s9d	^3^D_2_		P38G1	TW
6131.959(19)	16303.49	6131.925(9)	0.034	280	2.5e+6	D+		5s7p	^3^P°_1_	5s9d	^3^D_1_		P38G1	TW
6137.51(8)	16288.75	6137.48(3)	0.03	110	3.7e+6	D		5s6d	^1^D_2_	5s7f	^1^F°_3_	1	P38G1	TW
6139.88(4)	16282.46	6139.86(3)	0.02	2100				5s4f	^3^F°_2_	5s_1/2,_*_F_*_=5_6g			P38G2,1	
6141.27(4)	16278.76	6141.25(3)	0.02	1500				5s4f	^3^F°_2_	5s_1/2,_*_F_*_=4_6g			P38G2,1	
6141.78(6)	16277.41	6141.80(3)	−0.02	160				5s4f	^3^F°_3_	5s_1/2,_*_F_*_=5_6g			P38G2,1	
6143.20(6)	16273.67	6143.20(3)	0.00	2100				5s4f	^3^F°_3_	5s_1/2,_*_F_*_=4_6g			P38G2,1	
6148.10(6)	16260.68	6148.13(3)	−0.03	2100				5s4f	^3^F°_4_	5s_1/2,_*_F_*_=5_6g			P38G2,1	
6149.49(6)	16257.00	6149.53(3)	−0.04	2200				5s4f	^3^F°_4_	5s_1/2,_*_F_*_=4_6g			P38G2,1	
6161.09(8)	16226.41	6161.13(3)	−0.04	2000				5s4f	^1^F°_3_	5s_1/2,_*_F_*_=5_6g			P38G2,1	
6162.52(6)	16222.63	6162.54(3)	−0.02	4100				5s4f	^1^F°_3_	5s_1/2,_*_F_*_=4_6g		2	P38G2,1	
6224.27(8)	16061.69	6224.26(5)	0.01	120	6.3e+6	C		5s7p	^3^P°_2_	5s9d	^3^D_3_		P38G1	TW
6228.368(19)	16051.13	6228.363(13)	0.005	200	1.6e+6	D+		5s7p	^3^P°_2_	5s9d	^3^D_2_		P38G1	TW
6231.00(4)	16044.34	6230.979(10)	0.02	130*bl*	1.8e+5	D+		5s7p	^3^P°_2_	5s9d	^3^D_1_		P38G1	TW
6283.40(4)	15910.56	6283.39(3)	0.01	69	8.5e+6	C		5s7p	^1^P°_1_	5s9d	^1^D_2_		P38G1	TW
6302.636(20)	15861.99	6302.644(12)	−0.008	57	7e+5	E		5s7p	^3^P°_1_	5s10s	^1^S_0_	1	P38G1	TW
6304.874(16)	15856.36	6304.883(3)	−0.009	230	2.9e+6	D+		5s6s	^3^S_1_	5s6p	^1^P°_1_		P38G2,1	J07
6337.215(20)	15775.44	6337.212(11)	0.003	120	8e+5	D+		5s7p	^3^P°_0_	5s10s	^3^S_1_		P38G1	TW
6354.74(4)	15731.93	6354.758(13)	−0.02	110	3.2e+5	D+		5s7p	^1^P°_1_	5s9d	^3^D_2_		P38G1	TW
6362.252(20)	15713.36	6362.222(9)	0.030	230	2.2e+6	D+		5s7p	^3^P°_1_	5s10s	^3^S_1_		P38G1	TW
6437.006(21)	15530.88	6437.016(7)	−0.010	95	1.6e+6	D		5s6p	^3^P°_1_	5s7s	^1^S_0_		P38G1	J07
6468.975(21)	15454.13	6468.921(9)	0.054	270	3.9e+6	D+		5s7p	^3^P°_2_	5s10s	^3^S_1_		P38G1	TW
6473.23(4)	15443.98	6473.23(4)		24bl*				5s_1/2,_*_F_*_=4_5g		5s_1/2,_*_F_*_=4_14h			P38P	
6473.35(4)	15443.69	6473.35(4)		24bl*				5s_1/2,_*_F_*_=5_5g		5s_1/2,_*_F_*_=5_14h			P38P	
6541.180(21)	15283.54	6541.179(13)	0.001	150	7e+6	E		5s7p	^1^P°_1_	5s10s	^1^S_0_	1	P38G1	TW
6627.03(4)	15085.54	6627.03(4)		25bl*				5s_1/2,_*_F_*_=4_5g		5s_1/2,_*_F_*_=4_13h			P38P	
6627.16(4)	15085.25	6627.16(4)		25bl*				5s_1/2,_*_F_*_=5_5g		5s_1/2,_*_F_*_=5_13h			P38P	
6666.43(13)	14996.4	6666.385(3)	0.05	35	2.1e+6	C		5s6p	^1^P°_1_	5s6d	^3^D_2_		P38G1	J07
		6681.527(3)			1.5e+6	D+		5s6p	^1^P°_1_	5s6d	^3^D_1_			J07
6750.63(9)	14809.35	6750.59(4)	0.04	67				5s6d	^3^D_2_	5s6f	^1^F°_3_		P38G1	
		6751.52(4)			2.5e+5	E		5p^2^	^1^S_0_	5s8p	^1^P°_1_			B99
6751.88(5)	14806.60	6751.84(3)	0.04	160	1.5e+7	C+		5s6d	^3^D_1_	5s6f	^3^F°_2_		P38G1	B99
6765.91(9)	14775.89	6765.85(3)	0.06	160	1.5e+7	C+		5s6d	^3^D_2_	5s6f	^3^F°_3_		P38G1	B99
6767.36(9)	14772.74	6767.37(3)	−0.01	66	2.7e+6	C+		5s6d	^3^D_2_	5s6f	^3^F°_2_		P38G1	B99
6783.72(9)	14737.11	6783.68(6)	0.04	150	1.7e+7	C+		5s6d	^3^D_3_	5s6f	^3^F°_4_		P38G1	B99
6790.42(5)	14722.56	6790.42(3)	0.00	45	1.9e+6	C+		5s6d	^3^D_3_	5s6f	^3^F°3		P38G1	B99
6831.65(7)	14633.72	6831.65(7)		19bl*				5s_1/2,_*_F_*_=4_5g		5s_1/2,_*_F_*_=4_12h			P38G1	
6831.78(7)	14633.43	6831.78(7)		19bl*				5s_1/2,_*_F_*_=5_5g		5s_1/2,_*_F_*_=5_12h			P38G1	
6841.03(7)	14613.64	6841.04(4)	−0.01	6				5s5f	^3^F°_2_	5s_1/2,_*_F_*_=4_11g			P38G1	
6841.28(7)	14613.11	6841.25(4)	0.03	8				5s5f	^3^F°_3_	5s_1/2,_*_F_*_=5_11g			P38G1	
6842.90(9)	14609.65	6842.91(4)	−0.01	11				5s5f	^3^F°_3_	5s_1/2,_*_F_*_=4_11g			P38G1	
6848.79(14)	14597.1	6848.82(4)	−0.03	33				5s5f	^3^F°_4_	5s_1/2,_*_F_*_=5_11g			P38G1	
6850.45(9)	14593.56	6850.48(4)	−0.03	33				5s5f	^3^F°_4_	5s_1/2,_*_F_*_=4_11g			P38G1	
6860.16(9)	14572.90	6860.09(4)	0.07	15				5s5f	^1^F°_3_	5s_1/2,_*_F_*_=5_11g			P38G1	
6861.85(14)	14569.3	6861.76(4)	0.09	12				5s5f	^1^F°_3_	5s_1/2,_*_F_*_=4_11g			P38G1	
6867.70(14)	14556.9	6867.73(11)	−0.03	43	1.5e+6	D		5s7s	^3^S_1_	5s8p	^3^P°_2_		P38G1	B99
6891.5821(19)	14506.454	6891.5826(15)	−0.0005	3900	6.5e+7	B		5s6s	^3^S_1_	5s6p	^3^P°_2_		K01c	J07
6933.32(4)	14419.13	6933.343(23)	−0.02	34	1.3e+6	D		5s7s	^3^S_1_	5s8p	^3^P°_1_		P38G1	B99
		6952(5)			1.4e+6	D		5s7s	^3^S_1_	5s8p	^3^P°_0_			B99
7108.57(8)	14063.64	7108.572(21)	0.00	29				5s6d	^1^D_2_	5s9p	^3^P°_1_		P38G1	
7113.99(10)	14052.93	7113.99(10)		66bl*				5s_1/2,_*_F_*_=4_5g		5s_1/2,_*_F_*_=4_11h			P38G1	
7114.14(10)	14052.64	7114.14(10)		66bl*				5s_1/2,_*_F_*_=5_5g		5s_1/2,_*_F_*_=5_11h			P38G1	
7182.9038(21)	13918.110	7182.9048(15)	−0.0010	5800	5.6e+7	B		5s6s	^3^S_1_	5s6p	^3^P°_1_	2	K01c	J07
7218.22(10)	13850.02	7218.15(4)	0.07	14				5s5f	^3^F°_2_	5s_1/2,_*_F_*_=5_10g			P38G1	
7219.96(10)	13846.67	7219.99(3)	−0.03	14				5s5f	^3^F°_2_	5s_1/2,_*_F_*_=4_10g			P38G1	
7220.20(10)	13846.22	7220.23(4)	−0.03	25				5s5f	^3^F°_3_	5s_1/2,_*_F_*_=5_10g			P38G1	
7222.05(10)	13842.67	7222.07(4)	−0.02	71				5s5f	^3^F°_3_	5s_1/2,_*_F_*_=4_10g			P38G1	
7228.63(10)	13830.07	7228.66(4)	−0.03	74				5s5f	^3^F°_4_	5s_1/2,_*_F_*_=5_10g			P38G1	
7230.44(10)	13826.61	7230.51(3)	−0.07	61				5s5f	^3^F°_4_	5s_1/2,_*_F_*_=4_10g			P38G1	
7241.23(10)	13806.00	7241.22(4)	0.01	50				5s5f	^1^F°_3_	5s_1/2,_*_F_*_=5_10g			P38G1	
7243.06(10)	13802.51	7243.07(4)	−0.01	34				5s5f	^1^F°_3_	5s_1/2,_*_F_*_=4_10g			P38G1	
7254.981(11)	13779.836	7254.982(6)	−0.001	170	1.17e+7	B		5s6p	^3^P°_0_	5s7s	^3^S_1_		K01c	J07
7272.04(11)	13747.51	7272.05(7)	−0.01	15				5s7d	^3^D_2_	5s11f	^1^F°_3_		P38G1	
7273.07(11)	13745.56	7273.07(7)	0.00	15				5s7d	^3^D_1_	5s11f	^3^F°_2_		P38G1	
7276.637(3)	13738.827	7276.6388(21)	−0.002	2400	5.6e+7	B		5s6s	^3^S_1_	5s6p	^3^P°_0_		K01c	J07
		7282.35(7)		*m*				5s7d	^3^D_2_	5s11f	^3^F°_2_		P38	
		7284.30(7)		*m*				5s7d	^3^D_2_	5s11f	^3^F°_3_		P38	
7293.00(11)	13708.00	7292.96(8)	0.04	60				5s7d	^3^D_3_	5s11f	^3^F°_4_		P38G1	
7299.15(11)	13696.45	7299.13(7)	0.02	75*bl*				5s7d	^3^D_3_	5s11f	^3^F°_3_		P38G1	
7303.442(8)	13688.402	7303.443(4)	−0.001	330	1.09e+7	B		5p^2^	^1^D_2_	5s7p	^1^P°_1_		K01c	J07
7350.619(8)	13600.550	7350.619(6)	0.000	490	3.4e+7	B		5s6p	^3^P°_1_	5s7s	^3^S_1_		K01c	J07
7354.903(10)	13592.628	7354.903(9)	0.000	270	4.0e+7	B		5s6p	^1^P°_1_	5s7s	^1^S_0_		K01c	J07
7434.27(3)	13447.51	7434.258(17)	0.01	47	1.9e+6	D		5s6p	^3^P°_1_	5p^2^	^1^S_0_		P38G1	J07
7453.12(6)	13413.51	7453.140(23)	−0.02	230	1.2e+6	D+		5s7p	^3^P°_1_	5s8d	^1^D_2_		P38G1	TW
7520.79(11)	13292.82	7520.82(11)	−0.03	18				5s_1/2,_*_F_*_=4_5g		5s_1/2,_*_F_*_=5_10h			P38G1	
7522.4(3)	13290.0	7522.70(11)	−0.3	27bl*				5s_1/2,_*_F_*_=4_5g		5s_1/2,_*_F_*_=4_10h			P38G1	
7523.3(3)	13288.3	7523.03(11)	0.3	27bl*				5s_1/2,_*_F_*_=5_5g		5s_1/2,_*_F_*_=5_10h			P38G1	
7524.95(11)	13285.46	7524.91(11)	0.04	18				5s_1/2,_*_F_*_=5_5g		5s_1/2,_*_F_*_=4_10h			P38G1	
7602.86(3)	13149.33	7602.843(17)	0.02	220	6.8e+6	C		5s7p	^3^P°_0_	5s8d	^3^D_1_		P38G1	TW
7625.65(3)	13110.03	7625.683(6)	−0.03	51	9e+5	D		5p^2^	^1^D_2_	5s7p	^3^P°_1_		P38G1	J07
7632.80(3)	13097.74	7632.798(16)	0.00	270	8.5e+6	C		5s7p	^3^P°_1_	5s8d	^3^D_2_		P38G1	TW
7638.88(3)	13087.32	7638.869(15)	0.01	120	4.6e+6	C		5s7p	^3^P°_1_	5s8d	^3^D_1_		P38G1	TW
7682.978(18)	13012.21	7682.979(7)	−0.001	1500	5.6e+7	B		5s6p	^3^P°_2_	5s7s	^3^S_1_		K01c	J07
		7716.24(8)			1.4e+6	D+		5s7d	^3^D_1_	5s10f	^3^F°_2_			TW
		7726.69(8)			2.6e+5	D+		5s7d	^3^D_2_	5s10f	^3^F°_2_			TW
		7727.97(8)			1.5e+6	D+		5s7d	^3^D_2_	5s10f	^3^F°_3_			TW
7737.77(12)	12920.06	7737.80(8)	−0.03	37	1.7e+6	C		5s7d	^3^D_3_	5s10f	^3^F°_4_		P38G1,2	TW
7740.70(9)	12915.17	7740.69(5)	0.01	310	8.5e+6	C+		5s6d	^1^D_2_	5s6f	^1^F°_3_		P38G1,2	B99
		7744.66(8)			1.8e+5	D+		5s7d	^3^D_3_	5s10f	^3^F°_3_			TW
7760.81(12)	12881.71	7760.75(4)	0.06	42				5s6d	^1^D_2_	5s6f	^3^F°_3_		P38G1	
7776.95(3)	12854.98	7776.94(3)	0.01	320	1.2e+7	C		5s7p	^3^P°_2_	5s8d	^3^D_3_		P38G1	TW
7786.93(3)	12838.50	7786.885(17)	0.05	100	2.9e+6	C		5s7p	^3^P°_2_	5s8d	^3^D_2_		P38G1	TW
7788.99(6)	12835.10	7789.028(25)	−0.04	320	1.3e+7	C		5s7p	^1^P°_1_	5s8d	^1^D_2_		P38G1	TW
		7793.204(15)			3.2e+5	D+		5s7p	^3^P°_2_	5s8d	^3^D_1_			TW
7802.22(6)	12813.34	7802.17(3)	0.05	64				5s5f	^3^F°_2_	5s_1/2,_*_F_*_=5_9g			P38G1	
7804.31(6)	12809.91	7804.38(3)	−0.07	62				5s5f	^3^F°_2_	5s_1/2,_*_F_*_=4_9g			P38G1	
7804.63(12)	12809.38	7804.60(4)	0.03	39				5s5f	^3^F°_3_	5s_1/2,_*_F_*_=5_9g			P38G1	
7806.87(9)	12805.71	7806.81(4)	0.06	230				5s5f	^3^F°_3_	5s_1/2,_*_F_*_=4_9g			P38G1	
7814.49(9)	12793.22	7814.45(3)	0.04	230				5s5f	^3^F°_4_	5s_1/2,_*_F_*_=5_9g			P38G1	
7816.62(12)	12789.74	7816.67(3)	−0.05	120				5s5f	^3^F°_4_	5s_1/2,_*_F_*_=4_9g			P38G1	
7829.18(12)	12769.21	7829.12(4)	0.06	93				5s5f	^1^F°_3_	5s_1/2,_*_F_*_=5_9g			P38G1	
7831.36(12)	12765.66	7831.35(4)	0.01	40				5s5f	^1^F°_3_	5s_1/2,_*_F_*_=4_9g			P38G1	
7840.961(13)	12750.031	7840.974(7)	−0.013	2900	4.4e+7	B		5s6s	^1^S_0_	5s6p	^1^P°_1_		K01c	J07
7985.46(6)	12519.31	7985.457(18)	0.00	75	5.8e+5	D+		5s7p	^1^P°_1_	5s8d	^3^D_2_		P38G1	TW
		7992.103(16)			3.9e+5	D+		5s7p	^1^P°_1_	5s8d	^3^D_1_			TW
		8035.40(15)			5.5e+5	D+		5s8p	^3^P°_2_	5s14s	^3^S_1_			TW
8067.26(3)	12392.38	8067.237(18)	0.02	75	1.3e+6	D+		5s7p	^3^P°_1_	5s9s	^1^S_0_		P38G1	TW
8125.29(13)	12303.87	8125.19(9)	0.10	40	2.2e+5	E		5s7d	^1^D_2_	5s10f	^1^F°_3_	1	P38G1	TW
8153.74(13)	12260.94	8153.70(10)	0.04	32				5s_1/2,_*_F_*_=4_5g		5s_1/2,_*_F_*_=5_9h			P38G1	
8156.07(13)	12257.43	8156.05(10)	0.02	50bl*				5s_1/2,_*_F_*_=4_5g		5s_1/2,_*_F_*_=4_9h			P38G1	
8156.27(13)	12257.14	8156.30(10)	−0.03	50bl*				5s_1/2,_*_F_*_=5_5g		5s_1/2,_*_F_*_=5_9h			P38G1	
8158.64(13)	12253.58	8158.66(10)	−0.02	32				5s_1/2,_*_F_*_=5_5g		5s_1/2,_*_F_*_=4_9h			P38G1	
8191.63(3)	12204.23	8191.662(18)	−0.03	190	1.4e+6	D+		5s7p	^3^P°_0_	5s9s	^3^S_1_		P38G1	TW
8227.01(3)	12151.75	8226.982(6)	0.03	2100	2.4e+7	B		5s5d	^1^D_2_	5s6p	^1^P°_1_		P38G2	J07
8233.48(3)	12142.19	8233.500(15)	−0.02	480	3.9e+6	C		5s7p	^3^P°_1_	5s9s	^3^S_1_		P38G1	TW
		8409.72(3)			2.4e+6	C		5s7d	^3^D_1_	5s9f	^3^F°_2_			TW
8413.11(4)	11882.95	8413.080(15)	0.03	520	6.6e+6	C		5s7p	^3^P°_2_	5s9s	^3^S_1_		P38G1	TW
8422.23(14)	11870.08	8422.14(3)	0.09	71	4.5e+5	D+		5s7d	^3^D_2_	5s9f	^3^F°_2_		P38G1	TW
8423.51(14)	11868.28	8423.52(8)	−0.01	92	2.6e+6	C		5s7d	^3^D_2_	5s9f	^3^F°_3_		P38G1	TW
8434.53(14)	11852.77	8434.47(9)	0.06	250	2.9e+6	C		5s7d	^3^D_3_	5s9f	^3^F°_4_		P38G1	TW
		8443.35(8)			3.2e+5	D+		5s7d	^3^D_3_	5s9f	^3^F°_3_			TW
8462.27(4)	11813.91	8462.222(19)	0.05	240	1.2e+7	C		5s7p	^1^P°_1_	5s9s	^1^S_0_		P38G1	TW
8572.25(15)	11662.35	8572.268(9)	−0.02	72	1.1e+6	D+		5s6p	^1^P°_1_	5s7s	^3^S_1_		P38G1	J07
		8592.22(17)			8e+5	D+		5s8p	^3^P°_2_	5s13s	^3^S_1_			TW
8686.21(4)	11509.34	8686.233(24)	−0.02	340	4.4e+7	B		5s6p	^1^P°_1_	5p^2^	^1^S_0_		P38G1	J07
8797.96(12)	11363.15	8797.95(4)	0.01	110				5s5f	^3^F°_2_	5s_1/2,_*_F_*_=5_8g			P38G1	
8800.69(15)	11359.63	8800.71(4)	−0.02	110				5s5f	^3^F°_2_	5s_1/2,_*_F_*_=4_8g			P38G1	
8801.04(15)	11359.18	8801.03(5)	0.01	89				5s5f	^3^F°_3_	5s_1/2,_*_F_*_=5_8g			P38G1	
8803.86(12)	11355.53	8803.79(5)	0.07	360				5s5f	^3^F°_3_	5s_1/2,_*_F_*_=4_8g			P38G1	
8813.51(12)	11343.10	8813.56(4)	−0.05	820				5s5f	^3^F°_4_	5s_1/2,_*_F_*_=5_8g			P38G1	
8816.29(16)	11339.52	8816.33(4)	−0.04	82				5s5f	^3^F°_4_	5s_1/2,_*_F_*_=4_8g			P38G1	
8832.25(12)	11319.03	8832.23(5)	0.02	790				5s5f	^1^F°_3_	5s_1/2,_*_F_*_=5_8g			P38G1	
8835.12(16)	11315.36	8835.02(5)	0.10	110				5s5f	^1^F°_3_	5s_1/2,_*_F_*_=4_8g			P38G1	
9194.38(8)	10873.23	9194.41(5)	−0.03	340				5s4f	^3^F°_2_	5s_1/2,_*_F_*_=5_5g			P38G1	
9197.73(13)	10869.26	9197.71(5)	0.02	1300				5s4f	^3^F°_2_	5s_1/2,_*_F_*_=4_5g			P38G1	
9198.87(17)	10867.92	9198.76(5)	0.11	100				5s4f	^3^F°_3_	5s_1/2,_*_F_*_=5_5g			P38G1	
9202.06(17)	10864.15	9202.07(5)	−0.01	1400				5s4f	^3^F°_3_	5s_1/2,_*_F_*_=4_5g			P38G1	
9212.98(8)	10851.27	9212.97(5)	0.01	1500				5s4f	^3^F°_4_	5s_1/2,_*_F_*_=5_5g			P38G1	
9216.31(8)	10847.35	9216.29(5)	0.02	530				5s4f	^3^F°_4_	5s_1/2,_*_F_*_=4_5g			P38G1	
9242.13(17)	10817.05	9242.20(5)	−0.07	1400				5s4f	^1^F°_3_	5s_1/2,_*_F_*_=5_5g			P38G1	
9244.46(17)	10814.32	9244.46(17)		45				5s_1/2,_*_F_*_=4_5g		5s_1/2,_*_F_*_=4_8h			P38G1	
9244.70(17)	10814.04	9244.70(17)		45				5s_1/2,_*_F_*_=5_5g		5s_1/2,_*_F_*_=5_8h			P38G1	
9245.49(9)	10813.12	9245.54(5)	−0.05	450				5s4f	^1^F°_3_	5s_1/2,_*_F_*_=4_5g			P38G1	
		9246.633(10)			1.0e+6	D+		5s6s	^1^S_0_	5s6p	^3^P°_1_			J07
		9247.81(18)						5s_1/2,_*_F_*_=5_5g		5s_1/2,_*_F_*_=4_8h				
		9487.05(21)			1.1e+6	D+		5s8p	^3^P°_2_	5s12s	^3^S_1_			TW
		9622.65(12)			3.0e+6	C		5s7d	^3^D_1_	5s8f	^3^F°_2_			TW
		9637.24(9)			3.2e+6	C		5s7d	^3^D_2_	5s8f	^3^F°_3_			TW
		9638.91(12)			5.6e+5	D+		5s7d	^3^D_2_	5s8f	^3^F°_2_			TW
		9652.15(16)			3.6e+6	C		5s7d	^3^D_3_	5s8f	^3^F°_4_			TW
		9663.21(9)			3.9e+5	D+		5s7d	^3^D_3_	5s8f	^3^F°_3_			TW
		9788.225(8)			6e+5	D+		5s5d	^1^D_2_	5s6p	^3^P°_1_			J07
		10186.473(8)			1.92e+7	B		5p^2^	^1^D_2_	5s4f	^1^F°_3_			J07
		10239.76(3)			8.6e+5	B		5p^2^	^1^D_2_	5s4f	^3^F°_3_			J07
		10259.78(11)			2.7e+6	C		5s7d	^1^D_2_	5s8f	^1^F°_3_			TW
		10457(12)			1.8e+6	C		5s8p	^3^P°_0_	5s10d	^3^D_1_			TW
		10494.62(8)			2.1e+6	C		5s8p	^3^P°_1_	5s10d	^3^D_2_			TW
		10499.81(7)			1.2e+6	D+		5s8p	^3^P°_1_	5s10d	^3^D_1_			TW
		10632.41(12)			1.3e+6	D		5s8p	^1^P°_1_	5s10d	^1^D_2_	1		TW
		10640.4(3)			3.1e+6	C		5s8p	^3^P°_2_	5s10d	^3^D_3_			TW
		10648.6(3)			8e+5	D+		5s8p	^3^P°_2_	5s10d	^3^D_2_			TW
		10815.89(7)						5s5f	^3^F°_2_	5s_1/2,_*_F_*_=4_7g				
		10820.54(8)						5s5f	^3^F°_3_	5s_1/2,_*_F_*_=4_7g				
		10835.30(7)						5s5f	^3^F°_4_	5s_1/2,_*_F_*_=5_7g				
		10839.49(7)						5s5f	^3^F°_4_	5s_1/2,_*_F_*_=4_7g				
		10856.99(6)			2.27e+6	B		5s6d	^3^D_2_	5s5f	^1^F°_3_			J07
		10863.54(8)						5s5f	^1^F°_3_	5s_1/2,_*_F_*_=5_7g				TW
		10868.94(5)			3.7e+7	B		5s6d	^3^D_1_	5s5f	^3^F°_2_			J07
		10904.51(6)			3.6e+7	B		5s6d	^3^D_2_	5s5f	^3^F°_3_			J07
		10909.25(5)			6.8e+6	B		5s6d	^3^D_2_	5s5f	^3^F°_2_			J07
		10949.07(4)			4.3e+7	B		5s6d	^3^D_3_	5s5f	^3^F°_4_			J07
		10968.47(7)			4.6e+6	B		5s6d	^3^D_3_	5s5f	^3^F°_3_			J07
		11477.2(13)						5s_1/2,_*_F_*_=4_5g		5s_1/2,_*_F_*_=4_7h				
		11477.7(13)						5s_1/2,_*_F_*_=5_5g		5s_1/2,_*_F_*_=5_7h				
		11893.809(17)			1.39e+6	B		5s7p	^3^P°_1_	5s7d	^1^D_2_			J07
		12135.65(14)						5s6f	^3^F°_2_	5s_1/2,_*_F_*_=4_10g				
		12140.56(12)						5s6f	^3^F°_3_	5s_1/2,_*_F_*_=4_10g				
		12156.93(22)						5s6f	^3^F°_4_	5s_1/2,_*_F_*_=5_10g				
		12184.74(15)						5s6f	^1^F°_3_	5s_1/2,_*_F_*_=5_10g				
		12189.82(4)			5.4e+6	C		5s7d	^3^D_1_	5s7f	^3^F°_2_			TW
		12212.34(7)			5.7e+6	C		5s7d	^3^D_2_	5s7f	^3^F°_3_			TW
		12215.93(4)			9.9e+5	C		5s7d	^3^D_2_	5s7f	^3^F°_2_			TW
		12234.56(23)			6.4e+6	C		5s7d	^3^D_3_	5s7f	^3^F°_4_			TW
		12254.07(7)			7.0e+5	C		5s7d	^3^D_3_	5s7f	^3^F°_3_			TW
		12687.0(3)						5s_1/2,_*_F_*_=4_6g		5s_1/2,_*_F_*_=4_10h				
		12687.7(3)						5s_1/2,_*_F_*_=5_6g		5s_1/2,_*_F_*_=5_10h				
		12693.0(3)						5s_1/2,_*_F_*_=5_6g		5s_1/2,_*_F_*_=4_10h				
		12772.783(18)			2.38e+7	B		5s7p	^1^P°_1_	5s7d	^1^D_2_			J07
		12827.51(4)			1.6e+7	C+		5s7p	^3^P°_0_	5s7d	^3^D_1_			J07
		12901.147(21)			2.11e+7	B		5s7p	^3^P°_1_	5s7d	^3^D_2_			J07
		12930.400(22)			1.15e+7	C+		5s7p	^3^P°_1_	5s7d	^3^D_1_			J07
		13115.86(14)			7.8e+5	C		5s8p	^3^P°_1_	5s9d	^1^D_2_			TW
		13144.73(4)			2.9e+5	D		5s5d	^3^D_2_	5s6p	^1^P°_1_			J07
		13153.64(25)			9.8e+5	C		5s8d	^3^D_1_	5s10f	^3^F°_2_			TW
		13175.40(24)			1.0e+6	C		5s8d	^3^D_2_	5s10f	^3^F°_3_			TW
		13184.0(3)			1.2e+6	C		5s8d	^3^D_3_	5s10f	^3^F°_4_			TW
		13224.87(13)			4.9e+6	C		5s7d	^1^D_2_	5s7f	^1^F°_3_			TW
		13298.074(16)			2.9e+7	B		5s7p	^3^P°_2_	5s7d	^3^D_3_			J07
		13347.570(16)			7.3e+6	C+		5s7p	^3^P°_2_	5s7d	^3^D_2_			J07
		13373(20)			2.5e+6	C		5s8p	^3^P°_0_	5s9d	^3^D_1_			TW
		13378.885(18)			8.1e+5	C		5s7p	^3^P°_2_	5s7d	^3^D_1_			J07
		13430.71(10)			3.0e+6	C		5s8p	^3^P°_1_	5s9d	^3^D_2_			TW
		13442.88(10)			1.6e+6	C		5s8p	^3^P°_1_	5s9d	^3^D_1_			TW
		13533.67(20)			4.8e+6	C		5s8p	^1^P°_1_	5s9d	^1^D_2_			TW
		13664.2(5)			4.3e+6	C		5s8p	^3^P°_2_	5s9d	^3^D_3_			TW
		13668.85(9)			3.0e+7	B		5s6d	^1^D_2_	5s5f	^1^F°_3_			J07
		13683.9(4)			1.1e+6	C		5s8p	^3^P°_2_	5s9d	^3^D_2_			TW
		13744.26(10)			1.81e+6	B		5s6d	^1^D_2_	5s5f	^3^F°_3_			J07
		13882.94(17)						5s6f	^3^F°_2_	5s_1/2,_*_F_*_=4_9g				
		13889.37(15)						5s6f	^3^F°_3_	5s_1/2,_*_F_*_=4_9g				
		13910.6(3)						5s6f	^3^F°_4_	5s_1/2,_*_F_*_=5_9g				
		13941.828(22)			1.15e+6	C+		5s7p	^1^P°_1_	5s7d	^3^D_2_			J07
		13947.05(17)						5s6f	^1^F°_3_	5s_1/2,_*_F_*_=5_9g				
		13975.997(23)			8.3e+5	C+		5s7p	^1^P°_1_	5s7d	^3^D_1_			J07
		14599.0(4)						5s_1/2,_*_F_*_=4_6g		5s_1/2,_*_F_*_=4_9h				
		14599.3(4)						5s_1/2,_*_F_*_=5_6g		5s_1/2,_*_F_*_=5_9h				
		14601.58(11)			1.5e+6	C		5s8p	^3^P°_1_	5s10s	^3^S_1_			TW
		14789.01(21)			4.5e+6	C		5s8p	^1^P°_1_	5s10s	^1^S_0_			TW
		14901.4(5)			2.7e+6	C		5s8p	^3^P°_2_	5s10s	^3^S_1_			TW
		15240.92(4)			2.24e+6	B		5s7p	^3^P°_1_	5s8s	^1^S_0_			J07
		15305.09(11)			1.6e+6	C		5s8d	^3^D_1_	5s9f	^3^F°_2_			TW
		15334.1(3)			1.7e+6	C		5s8d	^3^D_2_	5s9f	^3^F°_3_			TW
		15343.4(3)			1.9e+6	C		5s8d	^3^D_3_	5s9f	^3^F°_4_			TW
		15909.62(8)			9.8e+6	B		5p^2^	^1^S_0_	5s7p	^1^P°_1_			J07
		15981.19(7)			1.03e+6	B		5s5d	^3^D_2_	5s6p	^3^P°_2_			J07
		16283.16(6)			3.8e+6	C		5s7p	^3^P°_0_	5s8s	^3^S_1_			J07
		16306.69(4)			1.12e+6	B		5s7s	^3^S_1_	5s7p	^1^P°_1_			J07
		16330.23(4)			5.5e+6	B		5s5d	^3^D_3_	5s6p	^3^P°_2_			J07
		16449.31(3)			1.0e+7	C		5s7p	^3^P°_1_	5s8s	^3^S_1_			J07
		16714.87(4)			2.8e+7	B		5s7p	^1^P°_1_	5s8s	^1^S_0_			J07
		16714.99(22)						5s5f	^3^F°_2_	5s_1/2,_*_F_*_=4_6g				
		16726.12(24)						5s5f	^3^F°_3_	5s_1/2,_*_F_*_=4_6g				
		16761.01(22)						5s5f	^3^F°_4_	5s_1/2,_*_F_*_=5_6g				
		16771.44(21)						5s5f	^3^F°_4_	5s_1/2,_*_F_*_=4_6g				
		16828.66(24)						5s5f	^1^F°_3_	5s_1/2,_*_F_*_=5_6g				
		17182.033(22)			1.8e+7	C		5s7p	^3^P°_2_	5s8s	^3^S_1_			J07
		17202.49(4)			1.44e+7	B		5s7s	^3^S_1_	5s7p	^3^P°_2_			J07
		17376.67(12)			1.31e+6	B		5s5d	^3^D_1_	5s6p	^3^P°_1_			J07
		17383.8(3)						5s6f	^3^F°_2_	5s_1/2,_*_F_*_=4_8g				
		17393.84(22)						5s6f	^3^F°_3_	5s_1/2,_*_F_*_=4_8g				
		17427.4(4)						5s6f	^3^F°_4_	5s_1/2,_*_F_*_=5_8g				
		17484.6(3)						5s6f	^1^F°_3_	5s_1/2,_*_F_*_=5_8g				
		17640.26(8)			3.8e+6	B		5s5d	^3^D_2_	5s6p	^3^P°_1_			J07
		17935.58(13)			5.0e+6	B		5s5d	^3^D_1_	5s6p	^3^P°_0_			J07
		18005.48(5)			1.19e+7	B		5s7s	^3^S_1_	5s7p	^3^P°_1_			J07
		18208.86(8)			1.25e+7	B		5s7s	^3^S_1_	5s7p	^3^P°_0_			J07
		18271(10)						5s_1/2,_*_F_*_=4_5g		5s_1/2,_*_F_*_=5_6h				
		18284(10)						5s_1/2,_*_F_*_=5_5g		5s_1/2,_*_F_*_=5_6h				
		18284(10)						5s_1/2,_*_F_*_=4_5g		5s_1/2,_*_F_*_=4_6h				
		18297(10)						5s_1/2,_*_F_*_=5_5g		5s_1/2,_*_F_*_=4_6h				
		18497.1(8)						5s_1/2,_*_F_*_=4_6g		5s_1/2,_*_F_*_=4_8h				
		18497.3(8)						5s_1/2,_*_F_*_=5_6g		5s_1/2,_*_F_*_=5_8h				
		19861.2(5)			2.3e+6	C		5s8d	^3^D_1_	5s8f	^3^F°_2_			TW
		19895.3(4)			2.4e+6	C		5s8d	^3^D_2_	5s8f	^3^F°_3_			TW
		19913.3(7)			2.7e+6	B		5s8d	^3^D_3_	5s8f	^3^F°_4_			TW
		20578.1(3)						5s7f	^3^F°_2_	5s_1/2,_*_F_*_=4_10g				
		20588.2(3)						5s7f	^3^F°_3_	5s_1/2,_*_F_*_=4_10g				
		20628.6(7)						5s7f	^3^F°_4_	5s_1/2,_*_F_*_=5_10g				
		20699.2(4)						5s7f	^1^F°_3_	5s_1/2,_*_F_*_=5_10g				
		20734.3(3)			1.16e+7	B		5s7d	^3^D_1_	5s6f	^3^F°_2_			TW
		20795.5(3)			1.21e+7	B		5s7d	^3^D_2_	5s6f	^3^F°_3_			TW
		20809.9(3)			2.1e+6	C		5s7d	^3^D_2_	5s6f	^3^F°_2_			TW
		20853.0(6)			1.35e+7	B		5s7d	^3^D_3_	5s6f	^3^F°_4_			TW
		20916.8(3)			1.5e+6	C		5s7d	^3^D_3_	5s6f	^3^F°_3_			TW
		21114.5(5)			2.4e+6	C		5s8d	^1^D_2_	5s8f	^1^F°_3_			TW
		23194.2(5)			8.7e+6	B		5s8p	^1^P°_1_	5s8d	^1^D_2_			TW
		23635.0(3)			6.2e+6	B		5s8p	^3^P°_1_	5s8d	^3^D_2_			TW
		23890.2(5)			1.27e+7	B		5s7d	^1^D_2_	5s6f	^1^F°_3_			TW
		24332.8(14)			8.7e+6	B		5s8p	^3^P°_2_	5s8d	^3^D_3_			TW
		27514.5(7)						5s6f	^3^F°_2_	5s_1/2,_*_F_*_=4_7g				
		27539.7(6)						5s6f	^3^F°_3_	5s_1/2,_*_F_*_=4_7g				
		27623.8(11)						5s6f	^3^F°_4_	5s_1/2,_*_F_*_=5_7g				
		27767.8(7)						5s6f	^1^F°_3_	5s_1/2,_*_F_*_=5_7g				
		30293(9)						5s_1/2,_*_F_*_=4_6g		5s_1/2,_*_F_*_=4_7h				
		30295(9)						5s_1/2,_*_F_*_=5_6g		5s1/2,*F*=57h				

a Observed wavelengths below 2000 Å are given in vacuum, above that in standard air. Conversion from vacuum to air wavelengths was made using the five-parameter formula from Peck and Reeder [28]. Estimated one-standard-deviation uncertainties are given in parentheses after the value in units of the least significant figure. If two uncertainties are given, the first is a statistical uncertainty, and the second is systematic. Uncertainties given for the air wavelengths do not include uncertainties of the air-vacuum conversion formula.

b Ritz wavelengths and their uncertainties were determined in the level optimization procedure using the LOPT code [23].

c Deviation of the observed wavelength from the Ritz value. This column is blank for lines that alone determine one of the two energy levels involved in the transition.

d Observed relative intensities have been converted to a uniform scale corresponding to emission from a plasma with an effective excitation temperature of about 3 eV (see Sec. 6.6).

e Transition probability values are followed by a letter code denoting the estimated uncertainty (see Table 3).

f Designations of transition types are as follows: E2 – electric quadrupole transition (enabled by an external electric field; see Sec. 2.3); HF – transition induced by hyperfine interaction.

g Notes: 1 – Transition probability value determined in the present work is uncertain because of strong cancellation effects see Sec. 7); 2 – Observed intensity value is unreliable (see Sec. 6.6); 3 – Observed intensity was marked as questionable in Paschen and Campbell [2].

h Codes for references to observed wavelengths and line identifications: B69 – Bhatia [5]; K01c – Karlsson and Litzén [11] (observed wavelengths have been corrected to account for a minor systematic error in the calibration of the wave number scale; see Sec. 2.1); L31 – Lang and Sawyer [3]; L93c – Larkins and Hannaford [8] (re-calibrated using the measurement of Wang *et al.* [27]; see Sec. 2.2); P38 – Paschen and Campbell [2]; this code followed by “P”, “TP”, “G1”, “G2”, etc. means the measurements made with a one-prism spectrograph, three-prism spectrograph, and a grating spectrograph in various orders of diffraction (see Sec. 2.3); W96 – lines identified by Wagatsuma [15] (no wavelength measurement was given, but the intensities were measured; see Sec. 2.6); Z01 – von Zanthier *et al.* [10].

i Codes for references to transition probability values: A86 – Ansbacher *et al.* [38]; A86c – The *A*-value was derived from the radiative lifetime measured by Ansbacher *et al.* [38] using transition rates for weaker decay branches calculated by Jönsson and Andersson [43] (see Sec. 7); B99 – Biémont and Zeippen [35]; B01 – Becker *et al.* [37]; CMEF00 – Curtis *et al.* [39]; J07 – Jönsson and Andersson [43]; L94 – Lavín and Martin [44]; M96 – Martinez *et al.* [41]; TW – This work.

**Table 2:** Optimized energy levels of In II

Configuration	Term	*J*	Level^a^ (cm^−1^)	Leading percentages^b^				Comments^c^
5s^2^	^1^S	0	0.000000000	98				
5s5p	^3^P°	0	42275.995245348(8)	100				
5s5p	^3^P°	1	43350.5817(15)	99				
5s5p	^3^P°	2	45829.256(6)	100				
5s5p	^1^P°	1	63038.546(10)	98				
5s6s	^3^S	1	93923.884(5)	100				
5s6s	^1^S	0	97030.212(12)	99				
5s5d	^1^D	2	97628.436(9)	67	31	5p^2^	^1^D	
5p^2^	^3^P	0	101608.06(20)	97				
5s5d	^3^D	1	102088.72(4)	100				
5s5d	^3^D	2	102174.69(3)	100				
5s5d	^3^D	3	102308.397(20)	100				
5p^2^	^3^P	1	103249.39(19)	100				
5p^2^	^3^P	2	105565.283(12)	96				
5s6p	^3^P°	0	107662.707(6)	100				
5s6p	^3^P°	1	107841.992(6)	96	4		^1^P°	
5s6p	^3^P°	2	108430.337(6)	100				
5s6p	^1^P°	1	109780.221(8)	94	4		^3^P°	
5p^2^	^1^D	2	113884.919(11)	58	27	5s5d	^1^D	
5p^2^	^1^S	0	121289.53(3)	56	42	5s7s	^1^S	
5s7s	^3^S	1	121442.541(12)	100				
5s7s	^1^S	0	123372.848(18)	57	39	5p^2^	^1^S	
5s4f	^3^F°	2	123642.95(3)	100				
5s4f	^3^F°	3	123648.096(24)	100				
5s4f	^3^F°	4	123664.85(3)	100				
5s4f	^1^F°	3	123699.170(10)	100				
5s6d	^3^D	1	124742.729(6)	100				
5s6d	^3^D	2	124776.714(6)	100				
5s6d	^3^D	3	124830.176(8)	100				
5s6d	^1^D	2	126670.945(8)	88	7	5p^2^	^1^D	
5s7p	^3^P°	0	126932.875(22)	100				
5s7p	^3^P°	1	126994.890(11)	91	9		^1^P°	
5s7p	^3^P°	2	127254.067(7)	100				
5s7p	^1^P°	1	127573.319(10)	90	9		^3^P°	
5s8s	^3^S	1	133072.511(7)	100				
5s8s	^1^S	0	133554.382(14)	98				
5s5f	^3^F°	2	133940.74(4)	100				
5s5f	^3^F°	3	133944.72(6)	100				
5s5f	^3^F°	4	133960.87(3)	100				
5s5f	^1^F°	3	133984.85(6)	100				
5s_1/2_,*_F_*_=4_5g			134512.23(6)					
5s_1/2,_*_F_*_=5_5g			134516.14(6)					
5s7d	^3^D	1	134726.488(10)	100				
5s7d	^3^D	2	134744.019(9)	100				
5s7d	^3^D	3	134771.897(9)	100				
5s7d	^1^D	2	135400.325(10)	95				
5s8p	^3^P°	0	[135823(11)]	100				LSF
5s8p	^3^P°	1	135861.62(5)	86	14		^1^P°	
5s8p	^3^P°	2	135999.37(23)	100				
5s8p	^1^P°	1	136096.93(9)	85	14		^3^P°	
5s9s	^3^S	1	139137.055(23)	100				
5s9s	^1^S	0	139387.30(5)	99				
5s6f	^3^F°	2	139549.42(10)	100				
5s6f	^3^F°	3	139552.75(7)	100				
5s6f	^3^F°	4	139567.37(14)	100				
5s6f	^1^F°	3	139586.14(8)	100				
5s_1/2,_*_F_*_=4_6g			139921.76(7)					
5s_1/2,_*_F_*_=5_6g			139925.47(7)					
5s_1/2,_*_F_*_=4_6h			[139980(3)]					POLAR
5s_1/2,_*_F_*_=5_6h			[139984(3)]					POLAR
5s8d	^3^D	1	140082.23(3)	100				
5s8d	^3^D	2	140092.64(4)	100				
5s8d	^3^D	3	140109.05(5)	100				
5s8d	^1^D	2	140408.36(5)	97				
5s9p	^3^P°	0	[140716(17)]	100				LSF
5s9p	^3^P°	1	140734.59(5)	85	15		^1^P°	
5s9p	^3^P°	2	140822.48(18)	100				
5s9p	^1^P°	1	140845.00(4)	84	15		^3^P°	
5s10s	^3^S	1	142708.324(25)	100				
5s10s	^1^S	0	142856.86(3)	99				
5s7f	^3^F°	2	142927.81(3)	100				
5s7f	^3^F°	3	142930.22(5)	100				
5s7f	^3^F°	4	142943.23(16)	100				
5s7f	^1^F°	3	142959.77(9)	100				
5s_1/2,_*_F_*_=4_7g			143183.87(5)					
5s_1/2,_*_F_*_=5_7g			143187.43(5)					
5s_1/2,_*_F_*_=4_7h			[143222.8(10)]					POLAR
5s_1/2,_*_F_*_=5_7h			[143226.3(10)]					POLAR
5s9d	^3^D	1	143298.47(4)	100				
5s9d	^3^D	2	143305.21(3)	100				
5s9d	^3^D	3	143315.78(14)	100				
5s9d	^1^D	2	143483.89(7)	97				
5s10p	^3^P°	0	[143676(17)]	100				LSF
5s10p	^3^P°	1	143706.54(8)	88			^1^P°	
5s10p	^3^P°	2	143740.9(3)	100				
5s10p	^1^P°	1	143763.10(8)	87			^3^P°	
5s11s	^3^S	1	144989.99(4)	100				
5s11s	^1^S	0	145086.25(4)	99				
5s8f	F°	2	145115.79(15)	100				
5s8f	^3^F°	3	145117.59(11)	100				
5s8f	^3^F°	4	145129.44(20)	100				
5s8f	^1^F°	3	145144.45(11)	100				
5s_1/2,_*_F_*_=4_8g			145300.34(4)					
5s_1/2,_*_F_*_=5_8g			145303.91(4)					
5s_1/2,_*_F_*_=4_8h			145326.55(21)					
5s_1/2,_*_F_*_=5_8h			145330.18(21)					
5s10d	^3^D	1	145382.99(4)	100				
5s10d	^3^D	2	145387.70(6)	100				
5s10d	^3^D	3	145394.94(4)	100				
5s10d	^1^D	2	145499.56(7)	98				
5s11p	^3^P°	1	145655.81(18)					
5s11p	^1^P°	1	145683.72(14)					
5s12s	^3^S	1	146537.16(4)	100				
5s12s	^1^S	0	146602.97(5)	99				
5s9f	^3^F°	3	146612.28(11)	100				
5s9f	^3^F°	2	146614.22(4)	100				
5s9f	^3^F°	4	146624.75(15)	100				
5s9f	^1^F°	3	146638.37(13)	100				
5s_1/2,_*_F_*_=4_9g			146750.53(5)					
5s_1/2,_*_F_*_=5_9g			146754.16(4)					
5s_1/2,_*_F_*_=4_9h			146769.69(15)					
5s_1/2,_*_F_*_=5_9h			146773.23(15)					
5s11d	^3^D	1	146812.50(9)					
5s11d	^3^D	2	146815.24(9)					
5s11d	^3^D	3	146820.60(14)					
5s11d	^1^D	2	146890.14(11)					
5s12p	^1^P°	1	[147016(10)]					RITZPL
5s13s	^3^S	1	147634.61(6)	100				
5s10f	^3^F°	3	147680.47(13)	100				
5s13s	^1^S	0	147681.43(7)	99				
5s10f	^3^F°	2	147682.61(14)	100				
5s10f	^3^F°	4	147691.91(15)	100				
5s10f	^1^F°	3	147704.35(16)	100				
5s_1/2,_*_F_*_=4_10g			147787.35(5)					
5s_1/2,_*_F_*_=5_10g			147790.88(6)					
5s_1/2,_*_F_*_=4_10h			147801.67(20)					
5s_1/2,_*_F_*_=5_10h			147804.99(20)					
5s12d	^3^D	1	147833.52(8)					
5s12d	^3^D	2	147836.29(17)					
5s12d	^3^D	3	147840.27(19)					
5s12d	^1^D	2	147888.97(15)					
5s14s	^3^S	1	148440.88(7)	100				
5s11f	^3^F°	3	148468.39(14)					
5s11f	^3^F°	2	148472.06(14)					
5s14s	^1^S	0	[148475.56(22)]	99				RITZPL
5s11f	^3^F°	4	148479.98(15)					
5s11f	^1^F°	3	148491.51(14)					
5s_1/2,_*_F_*_=4_11g			148554.36(7)					
5s_1/2,_*_F_*_=5_11g			148557.89(8)					
5s_1/2,_*_F_*_=4_11h			148565.16(21)					
5s_1/2,_*_F_*_=5_11h			148568.78(21)					
5s13d	^3^D	1	148589.72(20)					
5s13d	^3^D	2	148592.29(18)					
5s13d	^3^D	3	148595.76(20)					
5s13d	^1^D	2	148630.83(17)					
5s15s	^3^S	1	149050.95(8)	100				
5s12f	^3^F°	3	149067.58(24)					
5s12f	^3^F°	2	149070.69(20)					
5s15s	^1^S	0	149077.3(3)	99				
5s12f	^3^F°	4	149078.76(20)					
5s12f	^1^F°	3	149088.88(20)					
5s_1/2,_*_F_*_=4_12g			149137.36(10)					
5s_1/2,_*_F_*_=5_12g			149140.89(12)					
5s_1/2,_*_F_*_=4_12h			149145.95(16)					
5s_1/2,_*_F_*_=5_12h			149149.57(16)					
5s14d	^3^D	1	149165.33(18)					
5s14d	^3^D	2	149168.03(25)					
5s14d	^3^D	3	149170.15(18)					
5s14d	^1^D	2	[149196.30(22)]					RITZPL
5s16s	^3^S	1	149523.97(18)	100				
5s16s	^1^S	0	149544.20(20)	99				
5s_1/2,_*_F_*_=4_13g			149590.82(12)					
5s_1/2,_*_F_*_=5_13g			149594.31(12)					
5s_1/2,_*_F_*_=4_13h			149597.77(12)					
5s_1/2,_*_F_*_=5_13h			149601.39(12)					
5s15d	^3^D	1	[149614.22(22)]					RITZPL
5s15d	^3^D	2	149614.68(20)					
5s15d	^3^D	3	149617.48(18)					
5s15d	^1^D	2	149638.1(3)					
5s17s	^3^S	1	149897.11(20)	100				
5s17s	^1^S	0	[149913.51(22)]	99				RITZPL
5s_1/2,_*_F_*_=4_14g			149950.61(8)					
5s_1/2,_*_F_*_=5_14g			149954.09(7)					
5s_1/2,_*_F_*_=4_14h			149956.21(12)					
5s_1/2,_*_F_*_=5_14h			149959.83(12)					
5s16d	^3^D	1	149968.70(25)					
5s16d	^3^D	2	149971.5(3)					
5s16d	^3^D	3	149972.30(20)					
5s16d	^1^D	2	[149988.21(22)]					RITZPL
5s18s	^3^S	1	[150198.20(22)]	100				RITZPL
5s18s	^1^S	0	[150210.90(22)]	99				RITZPL
5s17d	^3^D	1	150258.0(5)					
5s17d	^3^D	2	150258.0(5)					
5s17d	^3^D	3	[150258.68(22)]					RITZPL
5s17d	^1^D	2	150271.9(3)					
5s19s	^3^S	1	[150443.52(22)]	100				RITZPL
5s19s	^1^S	0	[150453.83(22)]	99				RITZPL
5s18d	^3^D	1	150491.1(3)					
5s18d	^3^D	2	150493.5(4)					
5s18d	^3^D	3	150493.5(3)					
5s18d	^1^D	2	[150503.81(22)]					RITZPL
5s19d	^3^D	1	[150686.56(22)]					RITZPL
5s19d	^3^D	2	[150687.03(22)]					RITZPL
5s19d	^3^D	3	150687.8(3)					
In III (5s ^2^S_1/2_)	Limit		152200.10(22)					

a Excitation energies and their uncertainties have been determined from observed wavelengths using the least-squares level optimization code LOPT [23]. Uncertainties in terms of one standard deviation are given in parentheses after the value in the units of the least significant figure of the value. Values enclosed in square brackets correspond to unobserved or poorly measured levels. They were determined semi-empirically using series formulas or a parametric fitting; the method used is specified in the last column.

b The leading percentages have been determined in the present work by means of a parametric least-squares fitting using Cowan’s codes [30] (see Sec. 4).

c Methods used in semi-empirical determination of unobserved or poorly measured levels: RITZPL and POLAR – Ritz-type quantum-defect and polarization formulas fitted with Sansonetti’s computer codes [29]; LSF – Parametric least-squares fitting with Cowan’s codes [30].

**Table 3:** Transition probability uncertainty code

Letter	Uncertainty in *A*-value	Uncertainty in log(*gf*)
A	≤ 3 %	≤ 0.013
B+	≤ 7 %	≤ 0.03
B	≤ 10 %	≤ 0.04
C+	≤ 18 %	≤ 0.08
C	≤ 25 %	≤ 0.11
D+	≤ 40 %	≤ 0.18
D	≤ 50 %	≤ 0.24
E	> 50 %	> 0.24
